# CellMap: precision mapping of cellular landscape in spatial transcriptomics

**DOI:** 10.1093/nar/gkaf1484

**Published:** 2026-01-08

**Authors:** Hongjia Liu, Huamei Li, Amit Sharma, Guoyan Tang, Zongyu Xie, Yunyao Shen, Qiong Li, Chen Gong, Xiao Sun, Kun Luo, Hongde Liu

**Affiliations:** State Key Laboratory of Digital Medical Engineering, School of Biological Science and Medical Engineering, Southeast University, Nanjing 211189, PR China; Department of General Surgery, Nanjing Drum Tower Hospital, the Affiliated Hospital of Nanjing University Medical School, Nanjing 210008, PR China; Department of Integrated Oncology, Center for Integrated Oncology, University Hospital of Bonn, Bonn 53127, Germany; State Key Laboratory of Digital Medical Engineering, School of Biological Science and Medical Engineering, Southeast University, Nanjing 211189, PR China; State Key Laboratory of Digital Medical Engineering, School of Biological Science and Medical Engineering, Southeast University, Nanjing 211189, PR China; State Key Laboratory of Digital Medical Engineering, School of Biological Science and Medical Engineering, Southeast University, Nanjing 211189, PR China; State Key Laboratory of Digital Medical Engineering, School of Biological Science and Medical Engineering, Southeast University, Nanjing 211189, PR China; State Key Laboratory of Digital Medical Engineering, School of Biological Science and Medical Engineering, Southeast University, Nanjing 211189, PR China; State Key Laboratory of Digital Medical Engineering, School of Biological Science and Medical Engineering, Southeast University, Nanjing 211189, PR China; Department of Neurosurgery, the First affiliated hospital of Xinjiang Medical University, Urumqi 830054, PR China; State Key Laboratory of Digital Medical Engineering, School of Biological Science and Medical Engineering, Southeast University, Nanjing 211189, PR China

## Abstract

Integrating single-cell RNA sequencing and spatial transcriptomics is the current imperative to manually explore the landscape of cellular mixtures. Herein, we developed CellMap (https://github.com/liuhong-jia/CellMap), a computational tool that allows spatial transcriptomic spots to be resolved at single-cell resolution. CellMap combines strategies that incorporate the co-linearity of seed genes, the random forest model, and the linear assignment algorithm to achieve optimal assignment of single cells to spatial spots. Using comprehensive benchmarking across various platforms and tissue types, we demonstrated that CellMap outperforms existing methods.

## Graphical Abstract

**Graphical Abstract:**
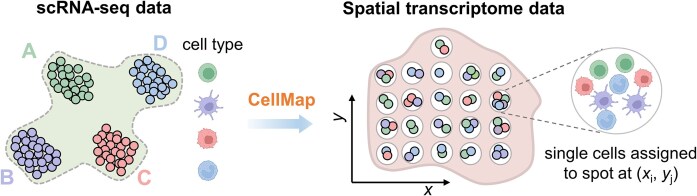

## Introduction

Both single-cell (SC) RNA sequencing (scRNA-seq) and spatial transcriptomics (ST) have emerged as pivotal methods to gain biological insights relevant for defining the cellular microenvironment [1, 2]. Strikingly, both approaches entail pros and cons that limit their complete success. For instance, scRNA-seq enables the characterization of gene expression and functional features of individual cells in tissues, thus provide crucial insights into deciphering cellular development, organizational function, and disease pathogenesis [3–5]. However, the essential tissue dissociation steps in scRNA-seq result in the loss of spatial information that is equally important for understanding the cellular microenvironment and cell–cell interactions [6]. On the other side, ST approaches based on next-generation sequencing (seq-based), such as 10X Visium and Slide-seq [7, 8], preserve the spatial positional information of cells within tissue sections, but are limited to measure primarily the small regions of mixed cell populations [9–11], which limits their application in resolving detailed tissue architecture and characterizing cell communication. Although ST approaches based on *in situ* hybridization and fluorescence microscopy (image-based), such as MERFISH [12], seqFISH [13], and STARmap [14], can detect the spatial distribution of transcripts with high resolution, they are limited by the total number of RNA transcripts. Nevertheless, it cannot be denied that both scRNA-seq and ST can complement each other well to make the utmost sense of biological datasets [15, 16]. This integration may contribute to the understanding of the structural distribution of cell types and provide new insights into tissue composition and functionality [17–19]. Several spatial deconvolution methods have already been proposed that aim to analyze the cellular composition within each spatial spot/patch based on SC data, e.g. SPOTlight [20], SpatialDWLS [21], RCTD [22], CARD [23], Cell2location [24], DestVI [25] Redeconve [26], SpatialDecon [27], and Stereoscope [28]. However, these methods are limited in dissecting cell types into “cell states” that may reflect different biological functions in more detail. Therefore, more sophisticated computational methods are required to effectively integrate ST and SC data, achieving spatial distribution characterization of the entire transcriptome at SC resolution [29–31].

Other notable approaches for spatial mapping of single cells include Seurat [32], Harmony [33], CellTrek [34], Tangram [35], CytoSPACE [36], NovoSpaRc [37], SpaOTsc [38], each offering their own unique capabilities. Seurat maps ST locations to individual single cells in the SC data by identifying “anchors” between the datasets. Harmony, on the other hand, typically maps individual cells to the nearest neighbor spot in the ST data to assign them. CellTrek uses ST data to train a multivariate random forest (RF) model that utilizes common dimensionality reduction features to predict spatial coordinates. In particular, a spatial nonlinear interpolation of ST data is introduced to increase the spatial resolution. Tangram uses a deep learning framework to learn spatial gene expression profiles at SC resolution and reveal complex spatial structures. CytoSPACE generates robust mappings from single cells to tissues by constrained global optimization, in addition, it can process the entire transcriptome without reducing preselected marker genes or shared embedding space, preserving sensitivity to mild cellular states. These methods can achieve SC resolution in ST data; however, the accuracy of deconvolving the cellular composition of spatial spots still needs to be improved. Therefore, a novel computational method is needed to achieve both accurate SC localization and robust inference of spot cellular composition simultaneously.

Herein, we have developed CellMap, a computational toolkit for mapping single cells from scRNA-seq profiles to precise spatial locations in ST data, suitable for a variety of ST sequencing platforms. As a unique approach, its input consists of paired SC data with well-annotated information and ST data, while the output comprises ST data with SC resolution and the cellular composition at spatial spots designed for downstream analysis. The presented method combines strategies that incorporate the co-linearity of seed genes, the RF model and the linear assignment algorithm to achieve optimal assignment of single cells to spatial spots. Most importantly, rigorous benchmarking analyses confirmed that CellMap can accurately reconstruct the spatial structures of different cell types within tissues, outperforming existing methods. CellMap thus serves as a valuable resource for spatial biological research.

## Materials and methods

CellMap uses two datasets as input: ST data, which serves as a template, and SC data, where the cell types are known but the information about cell positions remain unclear. The objective of CellMap is the precise mapping of individual cells to their respective positions within the template, i.e. the restoration of the spatial 2D position.

### Data preprocessing

Assuming the SC expression profile ($X$) was a matrix $m*n$, where $m$ was the number of genes and $n$ was the number of cells. The SC data were well annotated for $k$ different cell types. The ST expression profile (${\mathrm{S}}$) was a matrix $p*q,\ $where $p$ represented the number of genes and $q$ represented the number of spots. Data standardization was performed using the *SCTransform* function in Seurat for both SC ($X:m{\mathrm{*}}n$) and ST (${\mathrm{S}}:p{\mathrm{*}}q$) data, followed by dimensionality reduction and clustering. For the ST data, the spot cluster was determined on the basis of the clustering results (function *FindClusters* in Seurat), which were used as cluster labels for the subsequent analysis.

### Cell-type-specific genes

Cell-type-specific genes refer to genes that can be effectively differentiated between different cell types. We use a strategy of combining co-expression genes with seed genes as the core to identify specific genes that correspond to different cell types. The strategy encompasses the following steps:

1. Definition of seed genes: for the SC data, gene expression was averaged by cell type, yielding an $m*k$ matrix Y, where $m$ represented the number of genes, $k$ represented the number of cell types, and each value in the matrix denoted the average expression value of the gene in the corresponding cell type. We employed fold change (FC) to quantify the gene expression difference of a specific cell type compared to the average expression of other cell types. The FC value was defined by Eq. 1:

\begin{eqnarray*}
\mathrm{ FC }= \left\{ {\mathrm{ F}{{\mathrm{ C}}_{i{\mathrm{\ }}}}{\mathrm{\ }}|{\mathrm{\ }}i{\mathrm{\ }}{\mathrm{ from}}{\mathrm{\ }}1{\mathrm{\ }}\mathrm{ to}{\mathrm{\ }}m} \right\},{\mathrm{\ }}{\mathrm{ where}}
\end{eqnarray*}

(1) \begin{eqnarray*}
\ F{{C}_i} = \frac{{{{Y}_i}}}{{(\mathop \sum \nolimits_{j = 1}^k {{Y}_{ij\ }}) - {{Y}_i}}}{\mathrm{\ }} \cdot \left( {k - 1} \right){\mathrm{\ }}.
\end{eqnarray*}

In this equation, $FC$ is the FC value, ${\mathrm{\ }}{{{\mathrm{Y}}}_i}$ represents the expression of the $i$th gene in a specific cell type, and ${{Y}_{ij{\mathrm{\ }}}}$ represents the expression of the $i$th gene in the $j$th cell type. To ensure that highly expressed genes are more likely to be selected as seed genes, we added ${\mathrm{tanh\ }}$(Eq. 2) conversion weights to the $F{{C}_i}$.


\begin{eqnarray*}
F{{C}^{\mathrm{\text{'}}}}_i = \tanh \left( {\lambda {{W}_i}} \right) \cdot F{{C}_i},{\mathrm{\ where}}
\end{eqnarray*}

(2) \begin{eqnarray*}
{{W}_i} = \frac{{{\mathrm{median}}\left( {{{Y}_{i.}}} \right)}}{{{\mathrm{median}}\{ {\mathrm{median}}\left( {{{Y}_{t.}}} \right)|{\mathrm{t}} = 1,2,\ldots,{\mathrm{m}}\} }}{\mathrm{\ }},
\end{eqnarray*}

here $F{{C}^{\mathrm{\text{'}}}}_i$ is the final fold-change value, λ is an adjustment factor used for scaling weights, with a default value of 0.1, ${\mathrm{\ }}{{W}_i}$ is the weight for the $i$th gene, which contributes to higher specificity scores for highly expressed genes. The $\tanh ( {\lambda {{W}_i}} )$ suppresses linear growth when gene expression is extremely high, thereby balancing gene expression levels and $FC$. For a lowly expressed gene, $\tanh ( {\lambda {{W}_i}} )$ yields a low factor (the limit is 0), reducing the possibility that the gene is selected as a seed gene. In contrast, for a highly expressed gene, the value approaches 1. Therefore, if a gene demonstrates elevated expression in a specific cell type, $F{{C}^{\mathrm{\text{'}}}}_i$ will yield a high value; otherwise, it will approach 0. Subsequently, the $F{{C}^{\mathrm{\text{'}}}}_i$ values arranged in descending order, with the top 10 genes chosen as seed genes for each cell type by default.

The linear dimensionality reduction of the SC expression matrix was conducted based on principal component analysis (PCA): for the SC expression matrix *X*, we utilized a *z*-score normalization strategy (Eq. 3):
  (3) \begin{eqnarray*}
z{\mathrm{\ }} = \frac{{X - \bar{X}}}{{\mathrm{\sigma }}}{\mathrm{\ }}
\end{eqnarray*}
   
  (4) \begin{eqnarray*}
\sigma = \sqrt {\frac{1}{n}{\mathrm{\ }}\mathop \sum \limits_{i = 1}^n \left( {{{X}_i} - \bar{X}} \right)} {\mathrm{\ }},
\end{eqnarray*}

where $X$ and $z$ are the reference and normalized expression, and $\bar{X}$ and $\sigma {\mathrm{\ }}$(Eq. 4) are the mean and standard deviation of a gene from the SC expression matrix. Furthermore, PCA was employed to reduce the dimensionality of the standardized expression matrix for filtering out noise expression signals [39] to obtain the signal matrix ${\mathrm{Q}}$ (see Supplementary methods).

Calculation of genes co-expressed with seed genes.

Given that marker genes associated with the same cell type exhibit analogous expression profiles, they are expected to demonstrate a high level of correlation. Therefore, we proposed a co-expression-based method to identify genes with similar expression patterns to seed genes. Specifically, based on the signal matrix ${\mathrm{Q}}$ [${\mathrm{Q}}\epsilon {{{\mathrm{R}}}^{t \times m}},{\mathrm{\ }}$a dimensionality-reduced representation of the SC expression matrix, where $t$ is the number of principal components (PCs) and $m$ is the number of genes] obtained above, for each cell type, the Pearson correlation coefficients (PCCs) were used to measure the degree of correlation of other non-seed genes with seed genes. Since PCCs sampling distributions were not normal distribution, we applied the Fisher *Z*-transformation, which computed the inverse hyperbolic tangent (artanh) of correlation coefficients, to convert the sampling distribution into an approximately normal distribution with stable variance, enabling reliable significance testing. The transformation was calculated as Eq. 5:


(5) \begin{eqnarray*}
{{{\mathrm{z}}}_{ij}}{\mathrm{\text{'}}} = .5\left[ {\ln \left( {1 + {{{\mathrm{r}}}_{ij}}} \right) - \ln \left( {1 - {{{\mathrm{r}}}_{ij}}} \right)} \right]{\mathrm{\ }}.
\end{eqnarray*}

In this equation, ${{{\mathrm{r}}}_{ij}}$ represents the PCCs of the $i$th gene with the seed genes of $j$th cell type,${\mathrm{\ }}{{{\mathrm{z}}}_{ij}}{\mathrm{\text{'}}}$ is the transformed value of ${{{\mathrm{r}}}_{ij}}$. ${\mathrm{\ }}{{{\mathrm{z}}}_{ij}}{\mathrm{\text{'}\ }}$ follows a normal distribution, a *p*-value of correlation coefficient in ${{{\mathrm{z}}}_{ij}}{\mathrm{\text{'}}}$ is determined by *z*-test. We assigned the $i$th gene to the $j$th cell type if *p *< .01, ensuring the selection of highly significant specific genes for each cell type. Ultimately, the cell-type-specific gene list G was obtained for further analysis.

### Constructing a random forest classifier and predict

For both the SC ($X$) expression profile and ST ($S$) expression profile, we initially filter them using gene list G, obtained expression profiles $X^{\prime}( {g*n} )$ and $S^{\prime}( {g*q} )$, with rows corresponding to feature genes, columns represent cells/spots, each entry represents the expression value of genes within cells or spots. Considering the diverse viewpoints offered by geometric spatial distances to portray the relationship between single cells and spots. We next applied canonical correlation analysis (CCA) [32] of Seurat package to integrate SC and ST datasets. Furthermore, uniform manifold approximation and projection (UMAP) was employed to generate a two-dimensional projection of the integrated data. The Euclidean distance between single cells and spots was calculated based on their UMAP coordinates, resulting in a distance matrix composed of $n$ single cells and $q$ spots. Based on the distance matrix, the top *k* nearest neighbor single cells to each spot were selected to represent the features of the spot (for high-resolution ST data, set *k *= 1; for low-resolution ST data, set *k *= 5), and the cluster labels of the spots were transferred to the corresponding single cells. Subsequently, CellMap trained an RF model, employing the *randomForest* function from the randomForest package with a default of 1000 trees. The model was trained using the feature gene expression matrix of neighboring single cells along with the cluster labels. The trained RF model was then applied to the entire SC data to predict the cluster labels for each cell.

### Assessing the similarity between single cells and spots

Based on the cluster labels of single cells and spots, the single cells and spots belonging to the same cluster are combined. Within each cluster, cosine similarity was used to evaluate the similarity between single cells and spots, as it captured the directional similarity of gene expression patterns and was less sensitive to differences in expression level. This results in a similarity matrix $simi$, which was defined with Eq. 6.

$simi = \{ sim{{i}^z}| {z\,\textit{from}\,1\,to\,c\} } $ , where


(6) \begin{eqnarray*}
\ sim{{i}_{ij}}^z = \frac{{\mathop \sum \nolimits_{k = 1}^g ({{X}^\text{'}}_{ki}\ \times \ {{S}^\text{'}}_{kj})}}{{\sqrt {\mathop \sum \nolimits_{k = 1}^g {{X}^\text{'}}{{{_{ki}}}^{2\ }}} \ \times \ \sqrt {\mathop \sum \nolimits_{k = 1}^g {{S}^\text{'}}{{{_{kj}}}^{2\ }}} }}\ .
\end{eqnarray*}

In this equation, $simi\ $represents the similarity between single cells and spots, $c\ $represents the number of clusters. $\ sim{{i}_{ij}}^z$ represents the cosine similarity between $ith$ single cell and $jth$ spot in the $zth$ cluster. $\ {{X}^\text{'}}_{kj}$ represents the expression level of $kth$gene in $ith$ single cell. ${{S}^\text{'}}_{kj}$ represents the expression level of $kth\ $gene in${\mathrm{\ }}jth\ $spot. $g$ represents the number of feature genes. Therefore, higher $simi$ values indicate greater similarity between single cells and spots, while lower $simi$ values indicate less similarity.

### Assessing the number of cells in each spot

Given the mixed composition spots, it is crucial to estimate the number of single cells per spot beforehand. We extracted the count expression matrix from ST data and measured the variability of genes across different spots using analysis of variance. Genes were then sorted by their variance value, and those exhibiting a variance of < 0.5 (the default threshold) were automatically categorized as “stable genes.” In general, genes characterized by consistent expression levels were often less influenced by both biological and experimental factors, rendering them more reliable for estimating cell numbers. The total expression level of stable genes within each spot ($spot.{\mathrm{expr}}$) was calculated, along with the average expression level across all spots ($spot.\textit{mean}$). For low-resolution ST data, such as 10X Visium, where spots typically contain 1–10+ per spot, we utilized an average of five cells per spot throughout this study. For high-resolution ST data, such as Slide-seq V2, Visium HD, Stereo-seq, and MERFISH, we set the average cell number of one cell per spot. Therefore, the cell number for a specific spot could be calculated as: ${\mathrm{n}} = \frac{{{\mathrm{spot}}.{\mathrm{expr}}}}{{{\mathrm{spot}}.{\mathrm{mean}}}}{\mathrm{*}}\textit{mean}.\textit{cell}.num$, where $mean.\textit{cell}.num$ was assigned as 5 for low-resolution ST data and 1 for high-resolution ST data, and then rounded to the nearest integer.

### Allocating single cells to spatial locations

Within each cluster, the expression profile of single cells is denoted as $X\text{'} = g \times n\text{'}$, and the expression profile of spots as $S\text{'} = g \times q\text{'}$, where $g$ represents the shared feature genes, and $n\text{'}$ and $q\text{'}$ represent the number of single cells and spots, respectively. Based on the number of single cells within each spot ${{n}_s},s = 1,\ldots S,$ we hypothesize that ${{s}_{th}}$ spot contains ${{n}_s}$ sub-spots, and $N = \ \mathop \sum \limits_{s = 1}^S {{n}_s}$ represents the total number of sub-spots in the ST data. A linear allocation matrix $mat\ $of $n\text{'}\ \times N\ $is constructed by replicating the column of similarity values corresponding to the ${{s}_{th}}$ spot ${{n}_s}$ times in the cosine similarity matrix $simi$. If *n*$\text{'} > N$, to optimally assign single cells to sub-spots, we formulated the problem as a linear assignment problem that minimizes the overall cost defined as the dissimilarity between cells and sub-spots. The linear cost function can be described with Eq. 7:


(7) \begin{eqnarray*}
{\mathrm{\ }}\arg min\mathop \sum \limits_{i = 1}^{n\text{'}} \mathop \sum \limits_{j = 1}^N \left( {1 - mat} \right){{A}_{ij}}\ ,
\end{eqnarray*}

where ${{{\boldsymbol{A}}}_{{\boldsymbol{ij}}}}$ = 1 if single cell ${\boldsymbol{i}}$ is assigned to sub-spot$\ {\boldsymbol{j}}$. This setting ensures the global optimality of the entire allocation, where each cell is assigned to a sub-spot. The Jonker–Volgenant algorithm [40] was applied to realize such optimal allocation, which was solved with function *solve_LSAP* in R. If ${\boldsymbol{n}}\text{'} < {\boldsymbol{N}}$, wherein the presence of duplicated single cells within a spot is permissible, we implement a strategy based on the similarity matrix to selectively allocate a specific quantity of most similar single cells to the spot. Finally, we aggregate the allocations of single cells to spots within each cluster to obtain the final assignment for the entire ST section.

### Benchmark analysis with simulated datasets

To evaluate the precision and reliability of estimating cell number of each spot in CellMap, simulated ST data were generated using SC data from cerebral cortex of mouse. Specifically, we first generated a random cell number vector (λ) using Poisson distribution, ensuring its length matched the number of spots. Subsequently, for each spot, a specified number of single cells were sampled from the SC data, and gene expression was aggregated to obtain the corresponding gene expression profile, adding 1% transcriptional perturbation to better simulate the scenario of real ST data. Using λ values of 5, 10, and 20, PCCs were applied to assess the correlation between the predicted and actual cell number per spot.

To compare the performance of integration methods in predicting the cell type proportion of each spot, simulated ST datasets were generated using human HER2+ breast cancer SC data, with the HER2+ breast cancer ST data serving as a reference template. For the SC data, cells of each cell type were divided into train and test groups in a ratio of 1:1. In addition, the top 2000 variable genes were selected for the train set using *FindVariableFeatures* function in Seurat. The train set and the ST data were then integrated using CCA method of Seurat. Additionally, UMAP was employed to generate a two-dimensional projection of the merged SC and ST data. The Euclidean distance between each cell and each spot was calculated, resulting in a distance matrix. Based on the distance matrix, the top *k* (default value *k* = 5) nearest neighbor cells to each spot were identified and aggregated to represent the expression of the spot. Furthermore, synthetic ST datasets with different transcriptional noise (0%, 5%, and 20%) were generated to create simulated ST datasets with different noise levels. Similarly, to evaluate the impact of parameter settings on CellMap’s performance, we used human colorectal cancer (CRC) data to simulate high-resolution (*k *= 1) and low-resolution (*k* = 5) ST datasets under 0% transcriptional noise. The characteristics of these simulated spots were recorded, including the number of cells, proportion of cell types, to facilitate subsequent evaluations.

We used SC test set as a reference to analyze cell type proportion in simulated ST datasets with varying noise levels. Since the true cell type proportion of each spot in the simulated ST datasets is known and serves as ground truth, the performance of these methods was evaluated using root mean square errors (RMSEs) and PCCs. Specifically, the RMSEs and PCCs between the predicted and actual cell type proportion within each spot were calculated for each cell type.

### Benchmark analysis with real ST data using spot-level correlation

We calculated the spot-level Pearson’s correlation between cell type fractions and signature scores to evaluate the performance, as described previously [41]. For each cell type, the top 100 specific marker genes were identified as signature genes using the *FindAllMarkers* function in Seurat v4.4.0. The score for the signature genes is difference between the expression of the signature genes and the expression of equal-sized background genes, using the *AddModuleScore* function in Seurat v4.4.0. This approach provides an indirect but effective means of evaluating mapping accuracy in the absence of ground truth.

### Benchmark metrics

For the ST datasets with available ground truth, we used the following six metrics to assess the accuracy of integration methods.

PCC. The PCC value was calculated using Eq. 8:
  (8) \begin{eqnarray*}
{\boldsymbol{PCC}} = \frac{{{{\bf E}}\left[ {\left( {\widetilde {{{{\boldsymbol{x}}}_{\boldsymbol{i}}}} - \widetilde {{{{\boldsymbol{u}}}_{\boldsymbol{i}}}}} \right)\left( {{{{\boldsymbol{x}}}_{\boldsymbol{i}}} - {\mathrm{\ }}{{{\boldsymbol{u}}}_{\boldsymbol{i}}}} \right)} \right]}}{{\widetilde {{{{\boldsymbol{\sigma }}}_{\boldsymbol{i}}}}{{{\boldsymbol{\sigma }}}_{\boldsymbol{i}}}}}{\mathrm{\ }},
\end{eqnarray*}

where ${{{\boldsymbol{x}}}_{\boldsymbol{i}}}$ and $\widetilde {{{{\boldsymbol{x}}}_{\boldsymbol{i}}}}$ represent the composition vectors of cell type ${\boldsymbol{i}}$ in the ground truth and the predicted result, respectively; ${{{\boldsymbol{u}}}_{\boldsymbol{i}}}$ and $\widetilde {{{{\boldsymbol{u}}}_{\boldsymbol{i}}}}$ are the average composition value of cell type ${\boldsymbol{i}}$ in the ground truth and the predicted result, respectively; and ${{{\boldsymbol{\sigma }}}_{\boldsymbol{i}}}$ and $\widetilde {{{{\boldsymbol{\sigma }}}_{\boldsymbol{i}}}}$ are the s.d. of the composition of cell type ${\boldsymbol{i}}$ in the ground truth and the predicted result, respectively.

Structural similarity index (SSIM) . SSIM combines mean value, variance, and covariance to measure the similarity between the predicted result and the ground truth. It was calculated for each cell type with Eq. 9:
  (9) \begin{eqnarray*}
{{\mathrm{ SSIM}}} = \frac{{\left( {2\widetilde {{{{\boldsymbol{u}}}_{\boldsymbol{i}}}}{{{\boldsymbol{u}}}_{\boldsymbol{i}}} + {\boldsymbol{C}}_1^2} \right)\left( {2{{\bf cov}}\left( {{{{\boldsymbol{x}}}_{\boldsymbol{i}}}^{\prime},{{{\widetilde {{{{\boldsymbol{x}}}_{\boldsymbol{i}}}}}}^{\prime}}} \right) + {\boldsymbol{C}}_2^2} \right)}}{{\left( {{{{\widetilde {{{{\boldsymbol{u}}}_{\boldsymbol{i}}}}}}^2} + {{{\boldsymbol{u}}}_{\boldsymbol{i}}}^2 + {\boldsymbol{C}}_1^2} \right)\left( {{{{\widetilde {{{{\boldsymbol{\sigma }}}_{\boldsymbol{i}}}}}}^2} + {{{\boldsymbol{\sigma }}}_{\boldsymbol{i}}}^2 + {\boldsymbol{C}}_2^2} \right)}}\ ,
\end{eqnarray*}

where the definitions of ${{{\boldsymbol{u}}}_{\boldsymbol{i}}}$, $\widetilde {{{{\boldsymbol{u}}}_{\boldsymbol{i}}}}$,${\mathrm{\ }}{{{\boldsymbol{\sigma }}}_{\boldsymbol{i}}}$, and $\widetilde {{{{\boldsymbol{\sigma }}}_{\boldsymbol{i}}}}{\mathrm{\ }}$follow those for calculating the PCC value; ${{{\boldsymbol{C}}}_1}$ and ${{{\boldsymbol{C}}}_2}$ are 0.01 and 0.03, respectively; and ${{\bf cov}}( {{{{\boldsymbol{x}}}_{\boldsymbol{i}}}^{\mathrm{^{\prime}}},{{{\widetilde {{{{\boldsymbol{x}}}_{\boldsymbol{i}}}}}}^{\mathrm{^{\prime}}}}} )$ denotes the covariance between the composition of cell type ${\boldsymbol{i}}$ in the ground truth (${{{\boldsymbol{x}}}_{\boldsymbol{i}}}^{\mathrm{^{\prime}}}$) and that of the predicted result (${{\widetilde {{{{\boldsymbol{x}}}_{\boldsymbol{i}}}}}^{\mathrm{^{\prime}}}}$).

RMSE. The RMSE was calculated using the following equation on the z-scores of the composition for each cell type across all spots (Eq. 10):
  (10) \begin{eqnarray*}
{{\mathrm{ RMSE}}} = \sqrt {\frac{1}{{\boldsymbol{M}}}\mathop \sum \limits_{{\boldsymbol{j}} = 1}^{\boldsymbol{M}} {{{\left( {{{{{\boldsymbol{\tilde{Z}}}}}_{{\boldsymbol{ij}} - \ }}{{{\boldsymbol{Z}}}_{{\boldsymbol{ij}}}}} \right)}}^2}} \ ,
\end{eqnarray*}

where ${{{\boldsymbol{Z}}}_{{\boldsymbol{ij}}}}$ and ${{{\boldsymbol{\tilde{Z}}}}_{{\boldsymbol{ij}}{\mathrm{\ }}}}$ are the *z*-score of the composition of cell type ${\boldsymbol{i}}$ in spot ${\boldsymbol{j}}$ in the ground truth and the predicted result, respectively.

Jensen–Shannon (JS) divergence. JS uses relative information entropy (that is, Kullback–Leibler divergence) to determine the difference between two distributions. We first calculated the spatial distribution probability of each cell type (Eq. 11):
  (11) \begin{eqnarray*}
\ {{{\boldsymbol{P}}}_{{\boldsymbol{ij}}}} = \frac{{{{{\boldsymbol{x}}}_{{\boldsymbol{ij}}}}}}{{\mathop \sum \nolimits_{{\boldsymbol{j}} = 1}^{\boldsymbol{M}} {{{\boldsymbol{x}}}_{{\boldsymbol{ij}}}}}}\ ,
\end{eqnarray*}

where ${{{\boldsymbol{x}}}_{{\boldsymbol{ij}}}}$ is the composition of cell type ${\boldsymbol{i}}$ in spot ${\boldsymbol{j}}$, M is the total number of spots, and ${{{\boldsymbol{P}}}_{{\boldsymbol{ij}}}}$ is the distribution probability of cell type ${\boldsymbol{i}}$ in spot ${\boldsymbol{j}}$. We then calculated the JS value of each cell type using the following equations [42] (Eq. 12):


(12) \begin{eqnarray*}
JS = \frac{1}{2}\ KL\left( {\widetilde {{{P}_i}}{\mathrm{|}}\frac{{\widetilde {{{P}_i}} + {{P}_i}}}{2}} \right) + \frac{1}{2}\ KL\left( {{{P}_i}{\mathrm{|}}\frac{{\widetilde {{{P}_i}} + {{P}_i}}}{2}} \right)
\end{eqnarray*}

(13) \begin{eqnarray*}
KL({{a}_i}|{\mathrm{|}}{{b}_i}{\mathrm{)}} = \ \mathop \sum \limits_{j = 0}^M ({{a}_{ij}}\ \times \ log\frac{{{{a}_{ij}}}}{{{{b}_{ij}}}}) ,
\end{eqnarray*}

where ${{P}_i}$ and $\widetilde {{{P}_i}}$ denote the spatial distribution probability vectors of cell type $i$ in the ground truth and the predicted result, respectively; $KL({{a}_i}|{\mathrm{|}}{{b}_i}{\mathrm{)}}$ in Eq. 13 repersents the Kullback–Leibler divergence between two probability distribution ${{a}_i}$ and ${{b}_i}$, and ${{a}_{ij}}$ and ${{b}_{ij}}$ are the predicted and true probability of cell type $i$ in spot $j$, respectively.

ACCU. We defined the mapping accuracy as the proportion of spots for which the predicted cell type matched the ground truth (Eq. 14).
  (14) \begin{eqnarray*}
\textit{ACCU} = {\mathrm{\ }}\frac{1}{M}\mathop \sum \limits_{j = 1}^M 1\left( {\widehat {{{y}_j}} = {{y}_j}} \right),
\end{eqnarray*}

where $M$ is the total number of spots, $\widehat {{{y}_j}}$ is the predicted cell type for spot $j$, and ${{y}_j}$ is the ground truth cell type, $1( . )$ is an indicator function that equals 1 if the predicted and true cell types are identical, and 0 otherwise. It is worth noting that for the methods designed to assign each cell from scRNA-seq data to a spot of ST data, the mapped spot-level cell type can be directly calculated from the cell type annotation of the assigned single cell. Therefore, ACCU was determined by assessing the concordance between the predicted and true cell types for all spots. For the methods designed to deconvolve the cell-type composition of spots, the cell type with the highest proportion was designated as the assigned cell type, and ACCU was calculated accordingly.

Accuracy score (AS). To evaluate the relative accuracy of the integration methods for each dataset, we defined an indicator, named as AS, by combining PCC, SSIM, ACCU, RMSE, and JS. For one dataset, we first calculated the average PCC, SSIM, ACCU, RMSE, and JS of all cell types predicted by each method. The PCC, SSIM, and ACCU values were then ranked in ascending order to obtain RANK_PCC_, RANK_SSIM_, and RANK_ACCU_, where the method with the highest PCC/SSIM/ACCU value will have RANK_PCC/SSIM/ACCU_ = N (total number of methods), and lowest will have RANK_PCC/SSIM/ACCU _= 1. Conversely, RMSE and JS values were ranked in descending order to obtain RANK_RMSE_ and RANK_JS_, the method with the highest RMSE/JS value will have RANK_RMSE/JS_ = 1, and the lowest will have RANK_RMSE/JS_ = N. Finally, we calculated the average value of RANK_PCC_, RANK_SSIM_, RANK _ACCU_, RANK_RMSE _, and RANK_JS_ to obtain the AS value of each method (Eq. 15):
  (15) \begin{eqnarray*}
AS &=& \frac{1}{5}\big( RAN{{K}_{PCC}} + RAN{{K}_{\textit{SSIM}}} + RAN{{K}_{\textit{ACCU}}}\\ &&\ \ \ \ +\, RAN{{K}_{\textit{RMSE}}} + RAN{{K}_{JS}} \big)
\end{eqnarray*}

For each dataset, the method with the highest AS value was the best performing method. For one cell type, higher PCC, SSIM, and ACCU, and lower RMSE and JS indicate better prediction.

## Results

### Outline of CellMap

**Figure 1.**
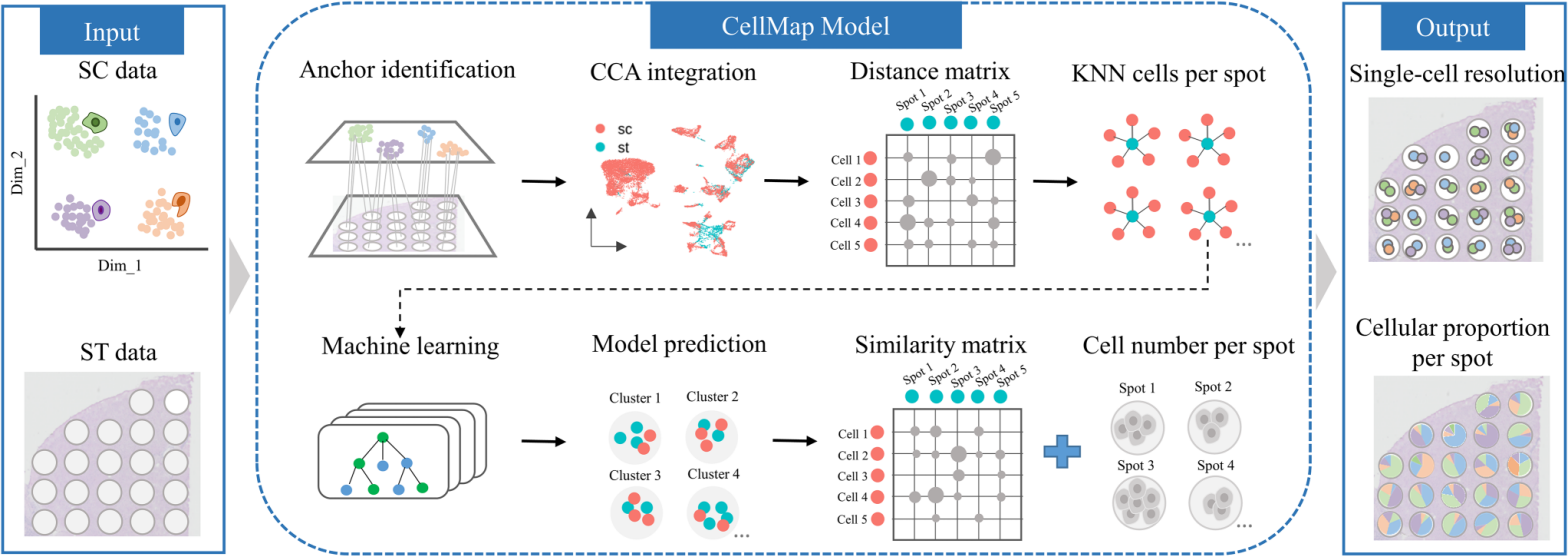
Outline of CellMap. CellMap is designed to infer spatial transcriptomic spots at SC resolution and resolve cell type proportion of spatial transcriptomic data by combining RF classifier and linear assignment algorithm. The workflow involves data integration, feature selection, construction of RF model, and mapping single cells to spatial locations. The output comprises spatial tissue slice data with SC resolution and the cell type proportion at spatial locations, available for downstream analysis.

The CellMap toolkit comprises four primary modules: data preprocessing, feature selection, construction of RF model, and mapping single cells to spatial locations (Fig. 1). The input to CellMap includes SC data and ST data from the same region or tissue source, with the SC data well annotated with cell types. The standard processing pipeline of Seurat package is applied to normalize both SC data and ST data. Given that not all genes accurately capture cell-type characteristics, identifying representative cell type-specific genes is essential. CellMap applies a strategy that combines co-expression genes with seed genes as a core, ultimately deriving a set of feature genes by merging cell-type-specific genes, which are then used for data integration and model training. Based on the shared feature genes, the Seurat’s CCA method is applied to integrate SC data and ST data. Subsequently, UMAP is employed to create a two-dimensional projection of the integrated data, and the Euclidean distance matrix between single cells and spots is determined based on spatial coordinates. For each spot, we selected the top *k* nearest neighbor single cells to represent its expression characteristics and transfer the cluster labels of the spot to the single cells. The feature matrix is composed of the expression matrix of feature genes in neighboring single cells, where the cluster labels are used as response variable for training an RF model. This trained model is then employed to predict the cluster labels for each cell of the entire SC data. Furthermore, cosine similarity is applied within each cluster to evaluate the similarity between single cells and spots. Next, a cost function was constructed based on the similarity matrix using the number of single cells in each spot as a constraint. A linear assignment algorithm was employed to achieve the globally optimal mapping from single cells to spots. Finally, a cost function was constructed based on the similarity matrix with the number of single cells in each spot as the constraint, and a linear assignment algorithm was employed to achieve the globally optimal allocation from single cells to spatial spots.

### CellMap reconstructs the hierarchical structure of the mouse cerebral cortex region

**Figure 2.**
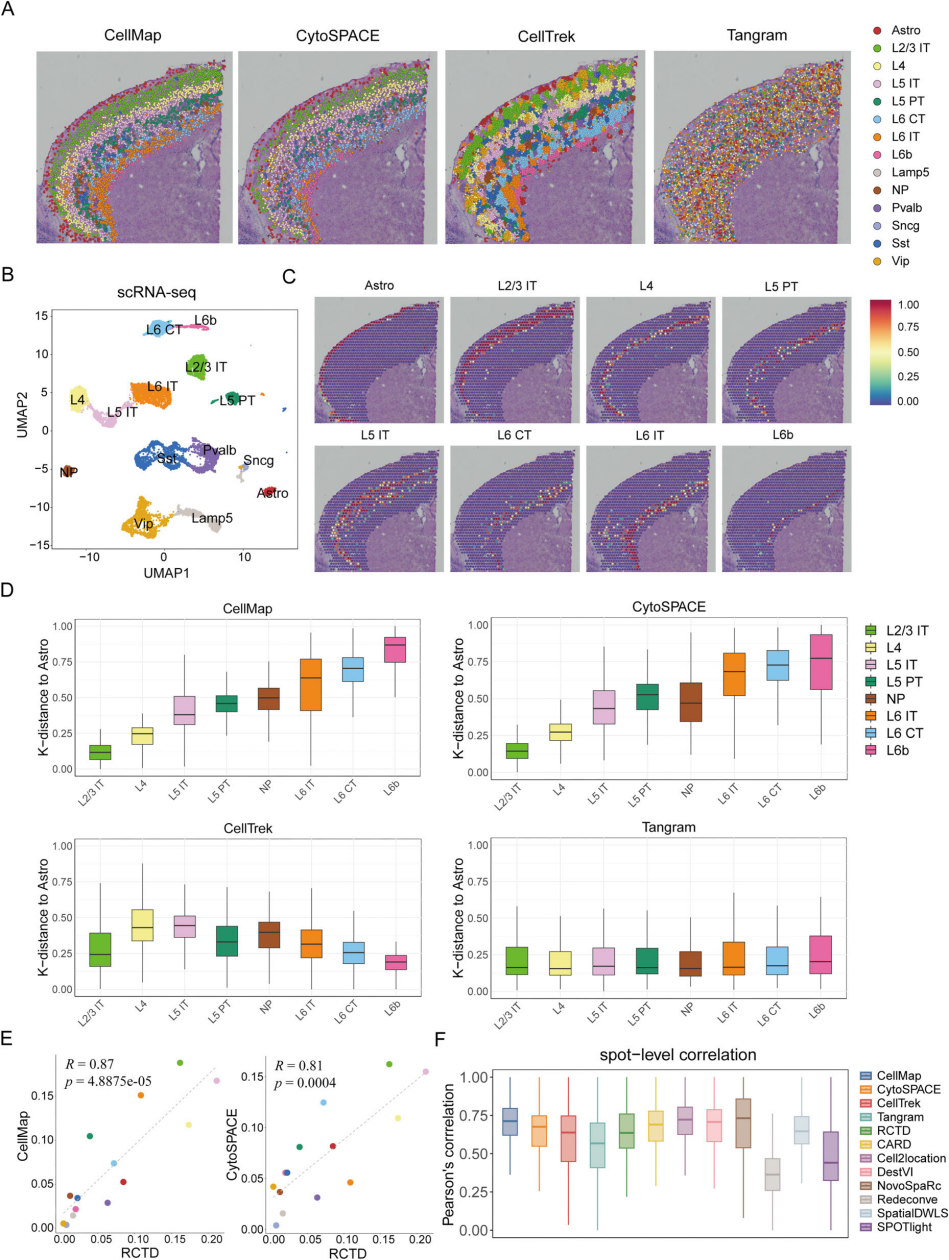
Performance assessment of CellMap on mouse cerebral cortex region. (**A**) Spatial structure of mouse cerebral cortex region reconstructed using CellMap, CytoSPACE, CellTrek, and Tangram. The cell types are color-coded, with each dot representing an individual cell. Only cell types with >100 cells are shown. (**B**) The UMAP layout depicting the clustering space of the scRNA-seq data. (**C**) Spatial structure of cell types including Astro, L2/3 IT, L4, L5 PT, L5 IT, L6 CT, L6 IT, and L6b on the ST slice of mouse cerebral cortex. The colors from blue to red indicate the cell proportions from low to high. (**D**) Boxplots illustrate the spatial k-distance (*k* = 10) of L2/3 IT, L4, L5 IT, L5 PT, NP, L6 IT, L6 CT, and L6b to Astro cells in mapping graphs generated by the four mapping methods. The boxplots display the median and quartile ranges (25%–75%), with whiskers extending up to 1.5× interquartile range from the box. (**E**) Scatter plots depicting the consistency between cell type proportions in spatial SCs maps reconstructed using CellMap and CytoSPACE, and cellular compositions predicted by RCTD spatial deconvolution method. “*R*” represents the PCCs and the *p*-values were obtained by *t*-test. Each point represents a cell type, with the corresponding colors referenced in panel (A). (**F**) Benchmark of CellMap’s performance with various methods. The box plot reflects the overall distribution of Pearson’s correlation calculated for each spot by each method.

To evaluate the performance of CellMap in reconstructing tissue structures with spatial patterns, we used 10X Visium ST data from the mouse cerebral cortex region, which exhibits a clear layered pattern (Supplementary Fig. S1A). Additionally, the corresponding SC data, consisting of 14 249 individual cells, covering 23 distinct cell types, was also used (Fig. 2B and Supplementary Table S1). Subsequently, CellMap was applied to the ST and SC data to reconstruct the spatial structure. As shown in Fig. 2A and C, CellMap successfully reconstructed the clear layered structure of the mouse cerebral cortex region, in the order of L2/3 IT, L4, L5 IT, L6 IT, L6 CT, and L6b. Thus, supporting the efficacy of CellMap in reconstructing the spatial organization of tissues. It is noteworthy that the cell-type-specific genes identified by CellMap exhibited high cell-type specificity (Supplementary Fig. S1B). Next, we compared the performance of CellMap with other SC spatial mapping methods, including CytoSPACE [36], CellTrek [34], and Tangram [35]. The results indicated that CytoSPACE showed a similar spatial layer structure (Fig. 2A and Supplementary Fig. S1D), followed by CellTrek (Fig. 2A and Supplementary Fig. S1E). However, Tangram did not effectively reconstruct the spatial structure of different cell types (Fig. 2A and Supplementary Fig. S1F). Additionally, for quantitative comparison of the performance of different methods in reconstructing the spatial structure of the mouse brain cortex region, we computed the spatial k-distances of different cell types to the “Astro” cells, where k-distance represents the mean Euclidean distance between each query cell and its *k* nearest cells in the reference population. The results indicated that the spatial k-distances exhibited an increasing trend from L2/3 to L6 in the mapped graph by CellMap and CytoSPACE, successfully recapitulating the cortical architecture. However, the other two methods were less effective in capturing this trend (Fig. 2D and Supplementary Table S2).

To validate the reliability of CellMap in analyzing cellular compositions in whole spatial slice, we next employed the deconvolution results of RCTD as the baseline for ST data. Notably, the results demonstrate that CellMap exhibits the highest correlation with RCTD (*R* = 0.87), followed by CytoSPACE (*R* = 0.81) (Fig. 2E), while CellTrek (*R* = 0.55) and Tangram (*R* = 0.28) perform less favorably (Supplementary Fig. S2).

We also compared the performance of 12 methods in predicting the cell-type compositions of spatial spots using spot-level correlation (Fig. 2F). These methods include CellMap, CytoSPACE, CellTrek, Tangram, RCTD [22], CARD [23], Cell2location [24], DestVI [25], NovoSpaRc [43], Redeconve [26], SpatialDWLS [21], and SPOTlight [20]. CellMap showed the high concordance (median PCC = 0.71) between the predicted proportion and corresponding feature gene scores of each cell type across all spots, comparable to NovoSpaRc (Fig. 2F and Supplementary Table S3). Finally, we assessed the performance of estimating the number of single cells per spot in CellMap by applying simulated ST data, with the mouse cerebral cortex tissue section as reference template (Supplementary Fig. S3A and B). For average cell counts of 5, 10, and 20, respectively, the predicted cell number per spot by CellMap showed high consistency with the actual number, comparable to the performance of CytoSPACE (Supplementary Fig. S3C). Hence, the aforementioned findings suggest that CellMap can effectively reconstruct the spatial structure of tissues and precisely infer cell type fraction within spatial spots.

### Performance evaluation of CellMap across biological and simulated datasets

**Figure 3.**
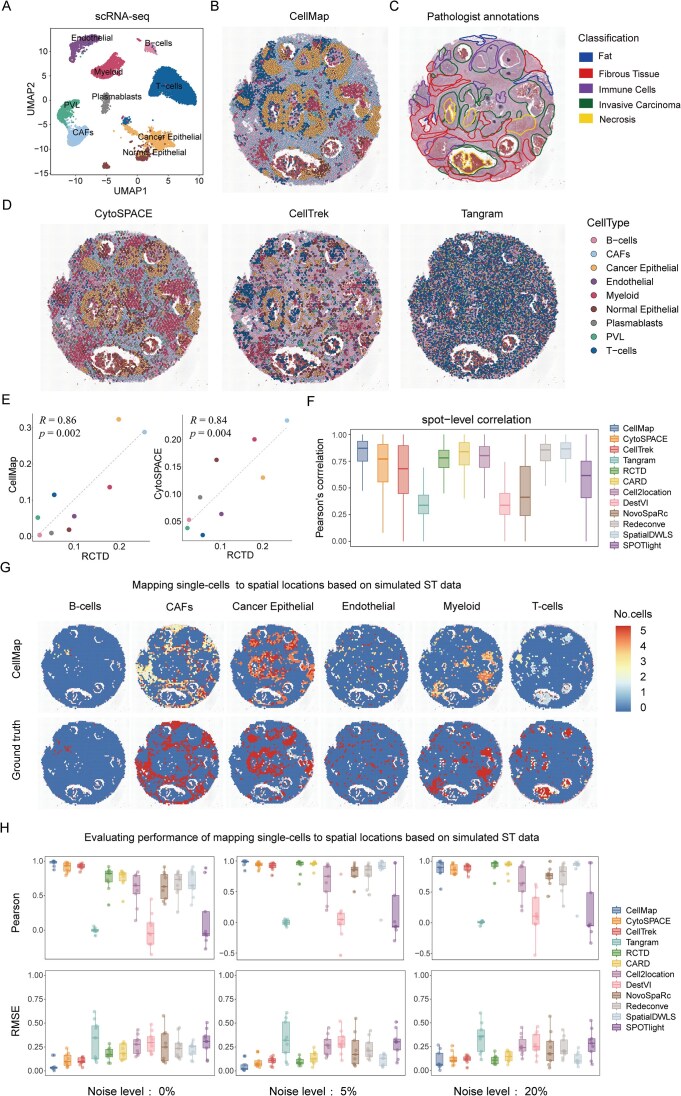
Performance assessment of CellMap on biological and simulated datasets. (**A**) The UMAP layout depicted the clustering space of HER2+ breast cancer scRNA-seq data. (**B**) Spatial structure of HER2+ breast cancer reconstructed using CellMap. The cell types are color-coded, with each dot representing an individual cell. (**C**) The pathological annotations of HER2+ breast cancer FFPE slice, including fat, fibros tissue, immune cells, invasive carcinoma and necrosis. (**D**) Spatial structure of HER2+ breast cancer reconstructed using CytoSPACE, CellTrek, and Tangram. The cell types are color-coded, with each dot representing an individual cell. (**E**) Scatter plots demonstrate the consistency between cellular composition in spatial SCs maps reconstructed using CellMap and CytoSPACE, and cellular composition predicted by RCTD spatial deconvolution method. “*R*” represents the PCCs and the *p*-values were obtained by *t*-test. Each point represents a cell type, with the respective colors referenced in panel (D). (**F**) Benchmark of CellMap’s performance with various methods. The box plot reflects the overall distribution of Pearson’s correlation calculated for each spot by each method. **(G)** Spatial heat maps depicting the performance of CellMap for aligning scRNA-seq data (with 5% added noise) to spatial locations in simulated ST dataset with each spot containing an average of five cells (see the “Materials and methods” section). For clarity, only cell types with distinct spatial structures are shown. The color intensity of each spot indicates the number of mapped single cells. (**H**) Boxplots depicting the PCCs and RMSEs between predicted and true cell type proportions in simulated ST datasets across varying noise levels (0%, 5%, and 20%), derived from outputs generated by 12 methods. Each data point represents a unique cell type. The center lines of the box, the box boundaries and the whiskers indicating the medians, the first and third quartiles and the minimum and maximum values respectively, within 1.5× interquartile range of the box borders.

We further validated CellMap’s mapping performance using the 10X Visium human HER2+ breast cancer FFPE dataset. To do this, we first used ST data with annotated pathology information where pathologists label the local tissue density and regions of tumors, immune cells, and fibroblasts without any prior information. Additionally, the corresponding SC data was used, consisting of 19 311 individual cells and covering nine cell types (Fig. 3A and Supplementary Table S1). We applied four spatial mapping methods to project single cells onto the tissue section. The results indicated that CellMap successfully reconstructed the spatial structures of different cell types within the HER2+ breast cancer FFPE dataset (Fig. 3B). The spatial distributions of various cell types corresponded to the annotations provided by pathologists, including cancer epithelial cells, cancer-associated fibroblasts (CAFs), B-cells, T-cells, etc. CytoSPACE followed CellMap, exhibiting similar mapping outcomes (Fig. 3D). However, CellTrek achieved sparse cell mapping and displayed unclear spatial structures of cell types (Fig. 3D). CellMap achieved the highest PCC (*R* = 0.86) when describing the cellular composition within the whole slice, followed by CytoSPACE (*R* = 0.84), both correlating well with the deconvolution of RCTD (Fig. 3E), CellTrek (*R* = 0.26) and Tangram (*R* = −0.115) exhibited weaker performance (Supplementary Fig. S4). At the spot-level correlation, CellMap (median PCC = 0.87) showed the highest concordance among the 12 methods (Fig. 3F and Supplementary Table S4).

We then simulated ST data at low resolution to assess the performance of CellMap more accurately. We applied HER2+ breast cancer SC data, with tissue section as reference template, to simulate ST data with known cell type localization and composition. Each spot aggregated the gene expression of its five nearest neighbor single cells (see “Materials and methods” section; Supplementary Fig. S5A). To simulate technical and platform variations between SC and ST data, we introduced different levels of noise (0%, 5%, and 20%) into the synthesized ST gene expression profiles (Supplementary Fig. S5B). We applied 12 methods to simulated ST datasets. The results indicated that the predicted cell number per spot by CellMap showed high consistency with the actual number for each cell type (Fig. 3G and Supplementary Fig. S5C). The predicted and actual cell type proportion within each spot exhibited the higher correlations (0.97, 0.97, and 0.87) and the lower RMSEs (0.03, 0.02, and 0.06), respectively, at different levels of noise (Fig. 3H, Supplementary Fig. S6, and  Supplementary Table S5).

To further demonstrate how CellMap elucidates spatial biology, we applied CellMap to 10X Visium data from mouse kidney and compared its performance with CytoSPACE, CellTrek, and Tangram in three aspects: the spatial structure of cell types, known zone areas and epithelial states (Supplementary Table S1). The results showed that CellMap reconstructed the spatial structures of different cell types located in different regions (Supplementary Fig. S7A and B) and achieved the highest PCCs (*R* = 0.96) with RCTD deconvolution results, similar to CytoSPACE (Supplementary Fig. S7C). Since the SC data were collected from different micro anatomical regions of the mouse kidney, including cortex, outer medulla, and inner medulla. Therefore, we calculated the k-distance from DistTub, Prin, and Vascular cells in different regions to a group of center spots. Of interest, a consistent decreasing trend in k-distance from cortex to outer medulla and then to inner medulla for CellMap (Supplementary Fig. S7D), indicating that CellMap effectively unveiled the zonal structure of mouse kidney. Furthermore, CellMap not only reconstructed the known zonal regions but also arranged nearly 30 epithelial states in the spots consistent with their known positions in the nephron epithelium and collecting duct system (Supplementary Figs S7E and S8). The predicted distances of epithelial states from the core inner medulla showed a high consistency with the known distances (*R* = 0.76), outperforming CellTrek and Tangram (Supplementary Fig. S7F–G and Supplementary Table S6). Briefly, after extensive analyses of both biological and simulated datasets, CellMap proves to be a powerful tool for reconstructing the spatial architecture and delineating the cellular composition within a spatial tissue section.

### Performance evaluation of CellMap using datasets with ground truth

**Figure 4.**
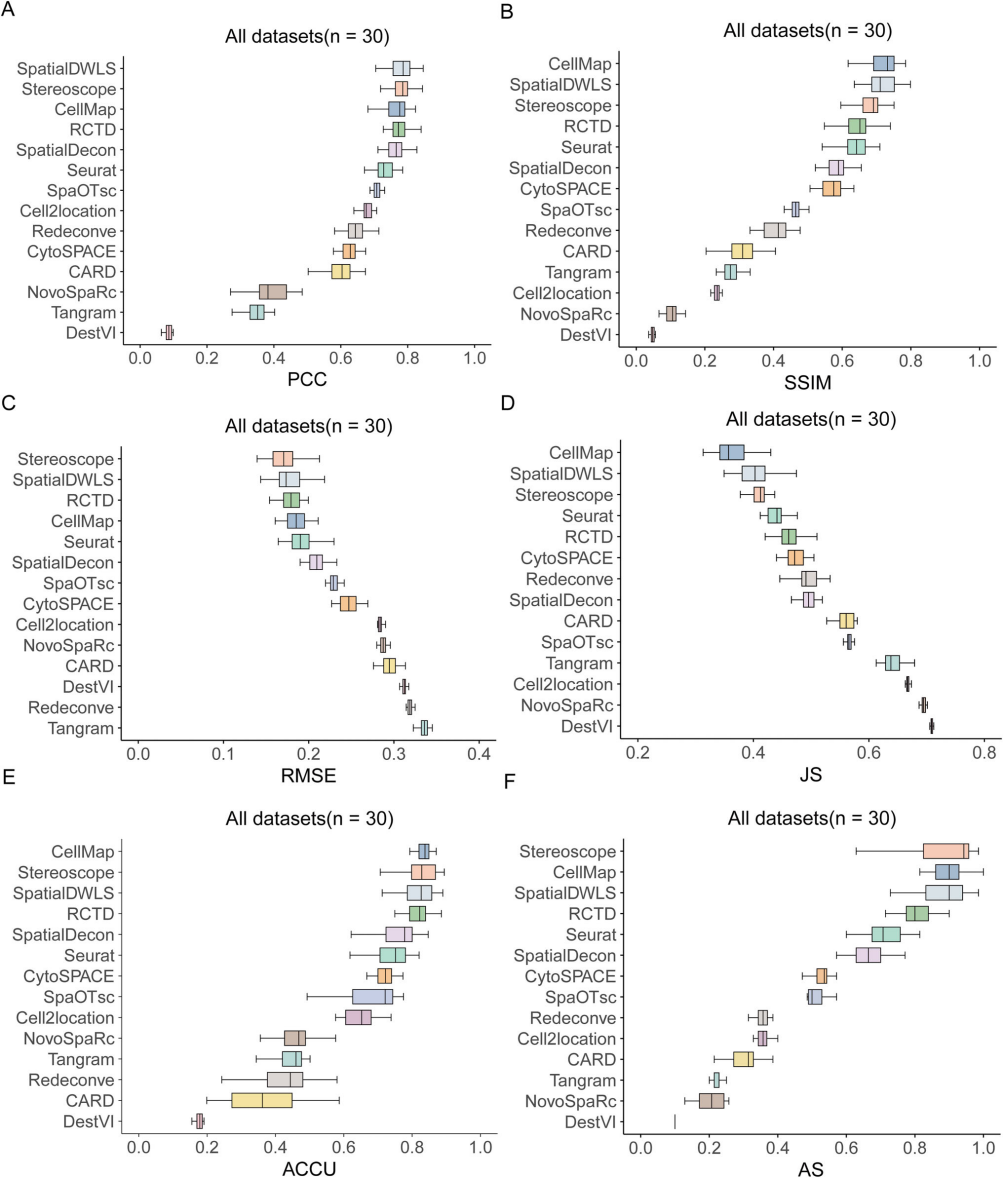
Comparing the performance of 14 integration methods for resolving the spatial distribution of cell types. (**A**–**D**) Boxplots of PCC, SSIM, RMSE, and JS of the 14 integration methods for all 30 paired datasets. Center line, median; box limits, upper and lower quartiles; whiskers, 1.5× interquartile range; *n* = 30 independent datasets. (**E**) Boxplots showing the mapping accuracy of 14 methods across 30 paired datasets. Center line, median; box limits, upper and lower quartiles; whiskers, 1.5× interquartile range; *n* = 30 independent datasets. (**F**) The boxplot of AS (which is aggregated from the PCC, SSIM, RMSE, JS, and ACCU) of the 14 integration methods for resolving the spatial distribution of cell types. Center line, median; box limits, upper and lower quartiles; whiskers, 1.5× interquartile range.

We then evaluated our method using MERFISH ST data from 30 slices of hypothalamic preoptic region [44]. In these datasets, for each spot, cell types had been annotated, thus serving as a form of “ground truth” for benchmarking purposes. The *in situ* cell-type identification in MERFISH datasets was realized as follows: first, scRNA-seq data was used to catalog cell populations and identify their marker genes; then, MERFISH imaging of these marker genes was done to identify cell populations and map their spatial organization *in situ*; finally, MERFISH was combined with expression of immediate early genes to identify discrete cell populations. We systematically benchmarked 14 spatial integration methods, including CellMap, CytoSPACE, Tangram, NovoSpaRc, SpaOTsc, Seurat, SpatialDecon, SpatialDWLS, RCTD, Stereoscope, Cell2location, CARD, Redeconve, and DestVI, across all 30 MERFISH datasets. Performance was assessed using six complementary metrics, including PCC, SSIM, RMSE, JS, ACCU, and AS (Supplementary Tables S7–S10). We computed the distribution of these metrics across the 30 datasets (Fig. 4). The results indicated that CellMap achieved the highest median SSIM (0.731), lowest median JS (0.357), and highest median ACCU (0.838) (Fig. 4B, D, and E). For PCC and RMSE, CellMap ranked third (0.776) and fourth (0.185), respectively (Fig. 4A and C). SpatialDWLS (0.786, 0.173), Stereoscope (0.785, 0.170), and RCTD (0.772, 0.179) performed better. Notably, the three methods were designed to deconvolve spot-level cell-type composition, while CellMap was designed for mapping single cells onto ST spots. Nevertheless, CellMap consistently outperformed other spatial mapping methods (Seurat, SpaOTsc, CytoSPACE, NovoSpaRc, and Tangram). Additionally, we aggregated the five metrics into a composite AS score to rank these methods. Although the median AS of CellMap (0.90) was slightly lower than that of Stereoscope (0.94), CellMap still outperformed all other mapping methods overall (Fig. 4F). Moreover, CellMap exhibited lower variance in median AS compared with Stereoscope, indicating greater generalizability and robustness (Fig. 4F and Supplementary Tables S11–S17).

On the averages of these metrics, CellMap exhibited the highest mean SSIM (0.72), the lowest mean JS (0.36), and the highest ACCU (0.83) (Supplementary Fig. S9B, D, and E). Although it ranked fourth for mean PCC and mean RMSE, it still outperformed all other spatial mapping methods (Supplementary Fig. S9A and C, and Supplementary Table S18). According to the mean AS score, CellMap emerged as the top performer (0.894), followed closely by Stereoscope (0.894), SpatialDWLS (0.879), and RCTD (0.80), all of which outperformed widely used spatial integration methods such as Seurat (0.714), CytoSPACE (0.529), SpaOTsc (0.509), Tangram (0.214), and NovoSpaRc (0.208) (Supplementary Fig. S9F). As exemplified by the MERFISH_12 dataset, CellMap’s predicted spatial distribution of cell types closely matched the ground truth (Supplementary Fig. S10), and exhibited the highest AS value (Supplementary Fig. S11). Taken together, the results demonstrated that CellMap consistently achieved superior performance in reconstructing the spatial distribution of cell types, with greater accuracy and robustness.

### Benchmarking CellMap across various spatial transcriptomics techniques

**Figure 5.**
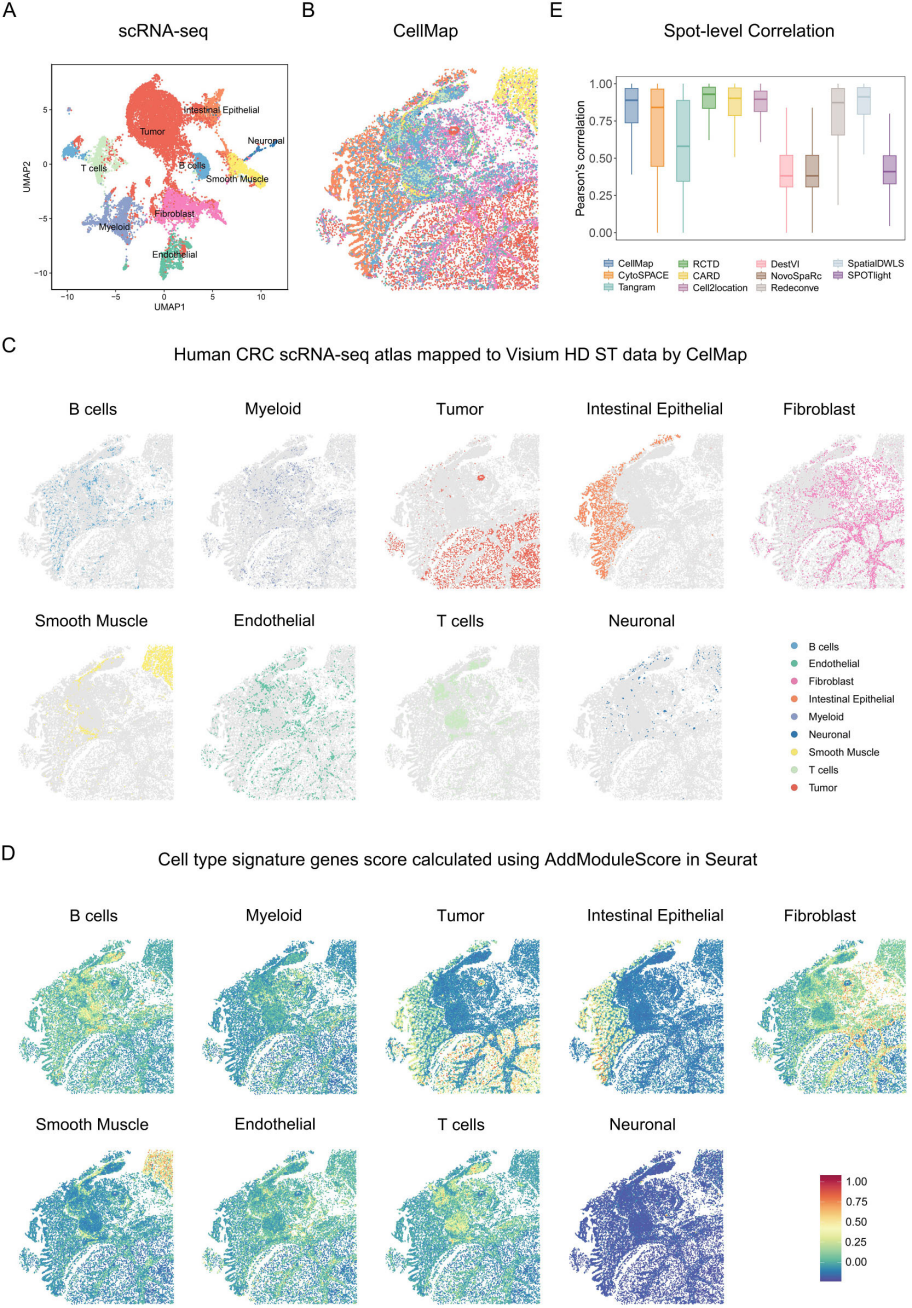
Benchmark CellMap on the Visium HD data from human CRC. (**A**)The UMAP layout depicting the clustering space of human CRC scRNA-seq data (B cells, Endothelial, Fibroblast, Intestinal Epithelial,Myeloid, Neuronal, Smooth Muscle, T cells, and Tumor). The cell types are color-coded, with each dot representing an individual cell. (**B**) Spatial structure of human CRC reconstructed using CellMap. (**C**) Spatial heat maps showing the spatial distribution of nine cell types predicted by CellMap in the Visium HD ST data, with each cell type highlighted in a different color. (**D**) Spatial heat maps showing cell type signature genes score calculated using AddModuleScore in Seurat. The colors from blue to red indicate the scores from low to high. (**E**) Benchmark of CellMap’s performance with different methods. The box plot reflects the overall distribution of Pearson’s correlation calculated for each spot by various method.

We then evaluated the performance of CellMap on multiple ST sequencing techniques, generated by Visium HD, Slide-seq V2, and Stereo-seq (Supplementary Table S1), in the paired scRNA-seq data and ST data from the same tissue samples. In human CRC Visium HD data, the paired SC data consists of 18 856 individual cells and includes nine cell types (Fig. 5A and B). The spatial distribution of the nine cell types showed a good match between the mapped by CellMap and the original ST data (Fig. 5C and D). We then calculated the spot-level correlation i.e. the correlation between cell type proportions and signature scores in spatial spots. Here again, CellMap showed a good performance and was comparable to the deconvolution methods RCTD and CARD (Fig. 5E and Supplementary Table S19).

The Slide-seq V2 data were derived from mouse cerebellum and hippocampus (Supplementary Table S1), of with the corresponding SC data comprising 15 197 and 52 846 individual cells and containing 19 and 16 cell types, respectively (Supplementary Figs S12A and S13A). We compared its performance with CytoSPACE and Tangram (Supplementary Figs S12B and S13B). Notably, CellMap successfully reconstructed the spatial organization of all cell types (Supplementary Figs S12C and S13C) and showed the highest correlation with the RCTD deconvolution in predicting the cellular composition of the whole slice (0.96, 0.85), outperforming CytoSPACE (0.48, 0.7) and Tangram (0.24, 0.57) (Supplementary Figs S12D and S13D). Also, CellMap exhibited the highest spot-level correlation in predicting the cell-type composition of spatial spots (median PCC = 0.75, 0.76) (Supplementary Figs S12E and S13E, and Supplementary Tables S20 and  S21).

In addition, we applied CellMap to Stereo-seq data corresponds to the mouse forebrain (Supplementary Table S1). The corresponding SC data consists of 10 537 individual cells, representing eight cell types (Supplementary Fig. S14A and B). CellMap (median PCC = 0.73) showed a comparable performance to CytoSPACE in spot-level correlation, outperforming Tangram (Supplementary Fig. S14C). As expected, the spatial location of each cell type predicted by CellMap largely matched the Stereo-seq data (Supplementary Fig. S14D and E, and Supplementary Table S22).

Building upon these representative cases, we systematically evaluated the performance of 14 spatial integration methods in resolving cell-type compositions within spots across 38 ST datasets, encompassing diverse tissue types, species, and sequencing platforms (10X Visium, Slide-seq, ST, seqFISH, MERFISH, Seq-scope [45], STARmap [46], and ISS [47]). Their corresponding scRNA-seq datasets were collected and cell types were annotated in advance (Supplementary Table S1). The evaluated methods included spatial mapping methods (CellMap, CytoSPACE, Tangram, NovoSpaRc, SpaOTsc, and Seurat) and spatial deconvolution methods (SpatialDecon, SaptailDWLS, RCTD, Stereoscope, Cell2location, CARD, Redeconve, and DestVI).

The performance was evaluated using spot-level correlation between cell type fractions and signature scores (see the “Materials and methods” section). It should be noted that for Dataset68 (seqFISH), Dataset69 (STARmap), Dataset70 (ISS), Dataset71 (seqFISH), and Dataset72 (MERFISH), which employ high-plex RNA imaging technologies, only a limited number of genes can be simultaneously detected per cell or spot [6]. Therefore, for each cell type, the top 10 specific marker genes were selected as signature genes, whereas the top 100 were selected for all other datasets. For each dataset, we computed the mean correlation across all spatial spots (Supplementary Table S23), and found that CellMap consistently exhibited good performance, surpassing multiple mapping methods (e.g. CytoSPACE, Tangram, NovoSpaRc) and achieving performance comparable to or exceeding deconvolution methods (e.g. RCTD, CARD) (Supplementary Fig. S15).

The aggregating results over the 38 datasets demonstrated that CellMap was favorable in overall performance. Although its median correlation (0.630) was slightly lower than that of Cell2location (0.656), it showed lower variance in mean spot-level correlation, suggesting better robustness (Supplementary Fig. S16A). Importantly, its mean correlation across all datasets was the highest (0.635) among the evaluated methods, exceeding those of Cell2location (0.629), Redeconve (0.615), SpaOTsc (0.612), RCTD (0.610), SpatialDWLS (0.609), CARD (0.595), DestVI (0.590), Seurat (0.590), CytoSPACE (0.584), NovoSpaRc (0.558), Stereoscope (0.551), Tangram (0.534), and SpatialDecon (0.487) (Supplementary Fig. S16B).

Overall, these results indicated that CellMap performed well across multiple ST platforms, including both high- and low-resolution datasets, demonstrating its robustness and broad applicability in resolving the cellular composition of spatial spots.

### Reasonableness assessment of CellMap’s built-in parameter settings

To ensure optimal performance and robustness of CellMap across datasets with varying resolutions, we evaluated the reasonableness of its built-in parameter settings by performing systematic sensitivity analyses on key parameters, including the number of seed genes and the average number of cells per spatial spot. For the *seed.num* parameter, CellMap was applied to simulated ST data of human HER2+ breast cancer, in which the true cell-type composition of each spot was known (see the “Materials and methods” section). The number of seed genes per cell type was varied from 10 to 50 in increments of 5, and performance was assessed using PCC, SSIM, JS, and RMSE. The optimal results achieved when 30 seed genes were used, which maximized PCC (0.968) and SSIM (0.956) while minimizing RMSE (0.058) and JS (0.127) (Supplementary Fig. S17 and  Supplementary Table S24). Therefore, we set *seed.num *= 30 as the default. To assess the impact of the average number of cells per spot on CellMap’s performance, we simulated high- and low- resolution ST datasets based on human CRC data (see the “Materials and methods” section). For the high-resolution dataset, the average number of cells per spot was varied from 1 to 5, and performance metrics did not exhibit obvious variation. Since this parameter determines the number of single cells that CellMap assigns to each spot, we set the default value of *mean.cell.num* = 1 for high-resolution ST data (Supplementary Fig. S18 and  Supplementary Table S25). For the low-resolution dataset, the average cell number per spot ranged from 5 to 10. The optimal performance achieved at five cells per spot. Accordingly, we set *mean.cell.num* = 5 as the default for low-resolution data (Supplementary Fig. S19 and  Supplementary Table S26).

In addition to the key parameters, we assessed the impact of UMAP parameter settings on the robustness of CellMap’s spatial mapping. Using the 30 MERFISH datasets with known ground truth, we found that performance remained consistent across a range of parameter values (Supplementary Figs S20–S22). Furthermore, we compared Euclidean distances calculated from UMAP coordinates and PCs of PCA, including the first two PCs and the set of PCs that collectively explained 90% of the total variance. The results demonstrated that Euclidean distance computation with UMAP coordinates outperformed that with PCs (Supplementary Fig. S23).

### The potential applications of CellMap

**Figure 6.**
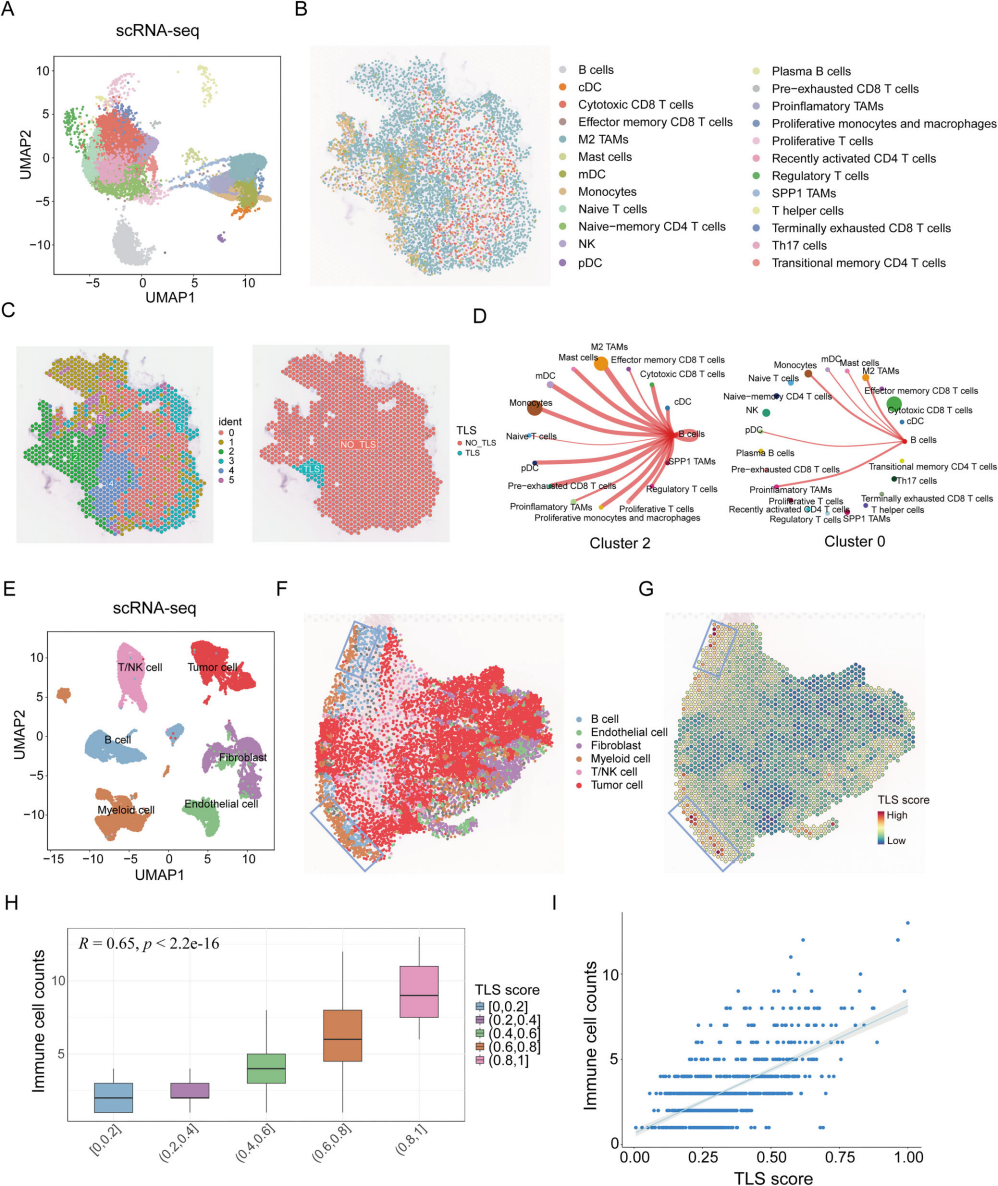
The application analysis of CellMap in reconstructing critical structural regions within tissues. (**A**) The UMAP layout depicted the clustering space of immune SC atlas. (**B**) Spatial structure of RCC tissue section reconstructed using CellMap. The cell types are color-coded, with each dot representing an individual cell. (**C**) Left: Spatial spot clustering of RCC tissue slice using the Seurat standard pipeline. Right: Spatial localization of predefined TLSs. (**D**) Circle plots illustrating the interactions between B cells and other cell types within individual spot clusters. The thickness of the edges reflects the strength of interactions. The size of dots represents the number of cells. Left: Cluster 2; Right: Cluster 0. (**E**) The UMAP layout depicted the clustering space of CRC scRNA-seq data (B cell, Endothelial cell, Fibroblast, Myeloid cell, T/NK cell, and Tumor cell). (**F**) The spatial SCs map reconstructed by CellMap. The blue boxes depicting potential locations of TLS with aggregation of immune cells. (**G**) The feature scoring of TLS at the ST spot level. The blue boxes depicting regions with high TLS scores. (**H**) The box plot illustrates the association between immune cell counts based on CellMap map and TLS score quantiles. A Pearson correlation test was performed. (**I**) The scatter plot depicting the association between immune cell counts and TLS scores, with the blue line represents linear fit, and the shaded area indicates the 95% confidence interval.

To validate the potential applications of CellMap, we acquired 10X Visium ST data containing tertiary lymphoid structures (TLS) in renal cell carcinoma (RCC), for which researchers have accurately annotated the regions where TLS reside (Supplementary Table S1). Intra tumoral TLS is known to correlate with positive clinical outcomes and responses to immunotherapy in cancer [48, 49]. Considering this, we next applied immune SC atlas, consisting of 18 340 individual cells representing twenty-five major immune cell types, to map onto the ST slice of RCC (Fig. 6A and B, and Supplementary Table S1). In cluster 2, which is characterized by the presence of TLS structures, B cells exhibited the most abundant interactions with other immune cells (Fig. 6C and D), which is consistent with previous findings [50]. To further explore whether CellMap can detect critical structural regions of tissues, we obtained 10X Visium ST data and corresponding SC data from patients with CRC, consisting of 27 845 individual cells comprising six major cell types (Fig. 6E). By mapping the SC data onto the CRC ST slice, we observed immune cells predominantly aggregate around tumor cells, including B cells, T/NK cells and myeloid cells. Thus, provide a reason to speculate that these areas may harbor TLS. Therefore, we calculated spot-level TLS scores using the annotated genes corresponding to TLS (Supplementary Table S27) and found that spots with high TLS scores corresponded to mixed immune cell aggregations in CellMap (Fig. 6F and G). Additionally, we found that the TLS scores at the spot level were positively correlated with the number of immune cells they contained (*R *= 0.65, *p* < 2.2e−16) (Fig. 6H and I). For comparison, we utilized three additional methods to map SC data onto CRC ST slice, showing CellMap’s superiority in analyzing critical tissue structural regions (Supplementary Fig. S24). In summary, these findings indicate that CellMap can not only explore the critical structural regions within tissues but also elucidate the intricate interactions among different cell types.

## Discussion

Current spatial biology relies on the advances in the successfully integration of scRNA-seq and ST based methodologies. Considering this, herein, we developed CellMap (https://github.com/liuhong-jia/CellMap), a computational tool that allows spatial transcriptomic spots to be resolved at SC resolution. The presented method is widely applicable to a variety of ST techniques, and aims to understand transcriptional heterogeneity in tissues from the perspective of single cell spatial resolution. CellMap is an innovative tool that enables the precise spatial mapping of individual cells to spatial locations within ST data, facilitating the creation of spatial SC 2D maps. In particular, CellMap introduces a strategy of combining co-expression genes with seed genes as the core to identify cell type-specific gene sets and thereby construct feature genes for subsequent analysis. In addition, it constructs an RF model based on the spatial relationship between individual cells and spots in two-dimensional space to classify individual cells across the entire SC dataset. Cross-validation analysis indicated that the most influential genes identified by the RF model exhibited distinct spatial expression patterns, which were consistent with established spatial structures and cell-type distributions (Supplementary Figs S25 and  S26). This not only demonstrated the model’s validity but also provided a biologically interpretable explanation for the model. Furthermore, it evaluates the cosine similarity between single cells and spots within each cluster, which is constrained by the number of single cells in each spot, and achieves optimal assignment of single cells to spatial spots using a linear assignment algorithm.

We did a comprehensive evaluation for CellMap and other integration methods. First, using mouse cerebral ST data, we visually demonstrated CellMap’s ability to accurately reconstruct the topological structure of cell types. Second, on the simulated ST data from the human HER2+ Breast cancer, we showed capacity of CellMap in noise tolerance and cell type proportion recovery compared to existing methods. Third, on the 30 MERFISH ST datasets with ground truth from the mouse hypothalamic preoptic region, we validated CellMap and 13 methods in literatures using six metrics (PCC, RMSE, SSIM, JS, ACCU, and AS). For SSIM, JS, and ACCU, CellMap exhibited the best among the methods. In terms of PCC and RMSE, CellMap’s performance was slightly lower than that of deconvolution methods, SpatialDWLS, Stereoscope, and RCTD (ranking third and fourth, respectively). However, CellMap still achieved the best results in the comprehensive metric, the mean of AS. Moreover, CellMap’s metrics exhibited smaller variations across all datasets, indicating more stable performance. The decreased performance of Cell2location in this context likely reflected its original design for spot-based cell-type deconvolution, whereas the MERFISH datasets provide subcellular-resolution measurements.

Finally, using ST data from multiple high-resolution platforms (Visium HD, Slide-seqV2, Stereo-seq, seqFISH, MERFISH, STARmap and ISS) and low-resolution platforms (10X Visium and ST), totally 41 datasets, we showed that CellMap exhibited broad applicability across diverse platforms and outperformed other methods. Shortly, CellMap is able to map single cells to spatial spots with high accuracy and robust performance, while simultaneously accurately resolving the cellular composition of spatial spots.

It should be noted that MERFISH datasets provides SC resolution, and thus spatial mapping is not strictly necessary. The goal that we included this dataset in this work is to assess CellMap’s performance on high-resolution ST data, since the dataset contained the cell type annotation, which can be used as “ground truth.” The accuracy of cell type annotations in MERFISH data depends on both the reliability of marker genes and the sensitivity of MERFISH imaging. We think the computational mapping will provide another validation for the MERFISH technique. We also demonstrated the potential application of CellMap in the restoration of disease-associated structures, TLSs.

In practical scenarios where SC reference data lack predefined cell-type annotations, we suggest to integrate our automated annotation tool, scAnno [39], into the analytical workflow. scAnno demonstrates full compatibility with CellMap, enabling robust cell-type label assignment prior to spatial mapping. This synergistic approach significantly expands CellMap’s utility beyond well-annotated datasets, ensuring broad applicability.

To summarize, CellMap (https://github.com/liuhong-jia/CellMap) represents a novel method to resolve spatial transcriptomic spots at SC resolution, it can easily determine transcriptional heterogeneity in tissues from the perspective of SC spatial resolution. Comprehensive analyses confirmed its superiority over other existing methods, positioning CellMap at the forefront of methods required to define the landscape of spatial biology.

## Supplementary Material

gkaf1484_Supplemental_Files

## Data Availability

The detailed information of the data is provided in Supplementary Table S1. The original data used in this paper is accessible through the following links: Dataset1 (mouse cerebral cortex):10X Visium, https://www.10xgenomics.com/cn/resources/datasets/mouse-brain-serial-section-1-sagittal-anterior-1-standard-1-1-0; Smart-seq, https://www.dropbox.com/s/dl/cuowvm4vrf65pvq/allen_cortex.rds [51]. Dataset2 (human HER2+ breast cancer): 10X Visium, https://zenodo.org/records/4739739; 10X Chromium, GSE176078 in the GEO database [52]. Dataset3 (mouse kidney): 10X Visium, https://www.10xgenomics.com/resources/datasets?query=&page=1&configure%5BhitsPerPage%5D=50&configure%5BmaxValuesPerFacet%5D=1000; 10X Visium, GSE171406 in the GEO database [53]; 10X Chromium, GSE129798 in the GEO database [54]. Dataset4 (human renal cell carcinoma): 10X Visium, GSE175540 in the GEO database [55]; Smart-seq2, https://zenodo.org/records/4263972. Dataset5 (human colorectal cancer): 10X Visium, GSE225857 in the GEO database; BD Rhapsody, GSE225857 in the GEO database [17]. Dataset6 (mouse cerebellum): Slide-seqV2, https://singlecell.broadinstitute.org/single_cell/study/SCP948; Smart-seq, https://singlecell.broadinstitute.org/single_cell/study/SCP948 [22]. Dataset7 (mosue hippocampus): Slide-seqV2, https://singlecell.broadinstitute.org/single_cell/study/SCP815/sensitive-spatial-genome-wide-expression-profiling-at-cellular-resolution#study-summary [56]; Drop-seq, GSE116470 in the GEO database [57]. Dataset8 (human colorectal cancer): Visium HD, https://www.10xgenomics.com/products/visium-hd-spatial-gene-expression/dataset-human-crc; 10X Chromium, https://www.10xgenomics.com/products/visium-hd-spatial-gene-expression/dataset-human-crc. Dataset9 (mouse developing cortex): Stereo-seq, https://db.cngb.org/stomics/mosta/spatial/ [58]; 10X Chromium, https://singlecell.broadinstitute.org/single_cell/study/SCP1290/molecular-logic-of-cellular-diversification-in-the-mammalian-cerebral-cortex [59]. Dataset10–Dataset39 (mouse hypothalamus): MERFISH data of mouse hypothalamic preoptic region at bregma 0.26 (ID = 1, 2, 3, 5, 6, 7, 12, 13, 14, 15, 16, 17, 18, 19, 20, 21, 22, 23, 24, 25, 26, 28, 29, 30, 31, 32, 33, 34, 35, 36), https://datadryad.org/stash/dataset/doi:10.5061/dryad.8t8s248 [44]; Drop-seq, GSE113576 in the GEO database [44]. Dataset40 (mouse hindlimb muscle): 10X Visium, Vis5A in GSE161318 in the GEO database [60]; 10X Chromium, GSE159500 in the GEO database [60]. Dataset41 (mouse hindlimb muscle): 10X Visium, Vis9A in GSE161318 in the GEO database; 10X Chromium, GSE159500 in the GEO database. Dataset42 (human breast cancer): 10X Visium,“CID3586” in https://zenodo.org/record/4739739#.YY6N_pMzaWC [61]; 10X Chromium, GSE176078 in the GEO database [61]. Dataset43 (human breast cancer): 10X Visium,“1160920F” in https://zenodo.org/record/4739739#.YY6N_pMzaWC; 10X Chromium, GSE176078 in the GEO database. Dataset44 (human breast cancer): 10X Visium,“CID4290” in https://zenodo.org/record/4739739#.YY6N_pMzaWC; 10X Chromium, GSE176078 in the GEO database. Dataset45 (human breast cancer): 10X Visium,“CID4465” in https://zenodo.org/record/4739739#.YY6N_pMzaWC; 10X Chromium, GSE176078 in the GEO database. Dataset46 (human breast cancer): 10X Visium,“CID44971” in https://zenodo.org/record/4739739#.YY6N_pMzaWC; 10X Chromium, GSE176078 in the GEO database. Dataset47 (human breast cancer): 10X Visium,“CID4535” in https://zenodo.org/record/4739739#.YY6N_pMzaWC; 10X Chromium, GSE176078 in the GEO database. Dataset48 (mouse embryo): 10X Visium, GSE160137 in the GEO database [62]; 10X Chromium, GSE143806 in the GEO database [62]. Dataset49 (mouse prostate): 10X Visium, “D25” in GSE159697 in the GEO database [63]; 10X Chromium, “V8” in GSE142489 in the GEO database [63]. Dataset50 (mouse kidney): 10X Visium, Sham Model in GSE171406 in the GEO database [53]; 10X Chromium, GSE171639 in the GEO database [53]. Dataset51 (mouse kidney): 10X Visium, ischemia reperfusion injury model in GSE171406 in the GEO database; 10X Chromium, wild-type ischemic acute kidney injury mouse in GSE171639 in the GEO database. Dataset52 (mouse brain): 10X Visium, GSE153424 in the GEO database [64]; 10X Chromium, https://www.dropbox.com/s/dl/cuowvm4vrf65pvq/allen_cortex.rds. Dataset53 (mouse prefrontal cortex): 10X Visium, GSE158450 in the GEO database [65]; 10X Chromium, https://www.dropbox.com/s/dl/cuowvm4vrf65pvq/allen_cortex.rds. Dataset54 (mouse hippocampus): 10X Visium, GSE158450 in the GEO database; Drop-seq, GSE116470 in the GEO database [65]. Dataset55 (mouse kidney): 10X Visium, GSE154107 in the GEO database [66]; 10X Chromium, GSE129798 in the GEO database [66]. Dataset56 (mouse prostate): 10X Visium, “D25” in GSE159697 in the GEO database; 10X Chromium, GSE142489 in the GEO database. Dataset57 (mouse lymph node): 10X Visium, https://github.com/romain-lopez/DestVI-reproducibility [67]; 10X Chromium, https://github.com/romain-lopez/DestVI-reproducibility. Dataset58 (mouse MCA205 tumor): 10X Visium, https://github.com/romain-lopez/DestVI-reproducibility; 10X Chromium, https://github.com/romain-lopez/DestVI-reproducibility. Dataset59 (mouse primary motor cortex); 10X Visium, https://console.cloud.google.com/storage/browser/tommaso-brain-data [68]; 10X Chromium, https://www.dropbox.com/s/dl/cuowvm4vrf65pvq/allen_cortex.rds. Dataset60 (mouse primary motor cortex): Slide-seq, https://console.cloud.google.com/storage/browser/tommaso-brain-data; 10X Chromium, https://www.dropbox.com/s/dl/cuowvm4vrf65pvq/allen_cortex.rds. Dataset61 (mouse cortex): seqFISH, https://github.com/CaiGroup/seqFISH-PLUS [13]; Smart-seq, https://www.dropbox.com/s/dl/cuowvm4vrf65pvq/allen_cortex.rds. Dataset62 (human squamous carcinoma): ST, GSE144239 (GSM4284322) in the GEO database [69]; 10X Chromium, GSE14236 in the GEO database [69]. Dataset63 (human hippocampus): ST, https://data.mendeley.com/datasets/6s959w2zyr/1 [70]; 10X Chromium, GSE116470 in the GEO database [57]. Dataset64 (mouse olfactory bulb): seqFISH, https://github.com/CaiGroup/seqFISH-PLUS; 10X Chromium, https://db.cngb.org/stomics/mosta/ [71]. Dataset65 (human osteosarcoma): MERFISH, https://www.pnas.org/doi/suppl/10.1073/pnas.1912459116/suppl_file/pnas.1912459116.sd12.csv [72];10X Chromium, GSE152048 in the GEO database [73]. Dataset66 (Zebrafish melanoma): 10X Visium, GSE159709 in the GEO database [74]; 10X Chromium, GSE159709 in the GEO database. Dataset67 (mouse liver): Seq-scope, https://deepblue.lib.umich.edu/data/downloads/gx41mj14n [75]; 10X Chromium, GSE185042 in the GEO database [76]. Dataset68 (mouse gastrulation): seqFISH, https://content.cruk.cam.ac.uk/jmlab/SpatialMouseAtlas2020/ [77]; 10X Chromium, https://bioconductor.org/packages/release/data/experiment/html/MouseGastrulationData.html [77]. Dataset69 (mouse visual cortex): STARmap, https://www.starmapresources.com/data [46]; Smart-seq, https://www.dropbox.com/s/dl/cuowvm4vrf65pvq/allen_cortex.rds. Dataset70 (mouse primary visual cortex (VISp)): ISS, https://github.com/spacetx-spacejam/data [78]; Smart-seq, https://portal.brain-map.org/atlases-and-data/rnaseq/mouse-v1-and-alm-smart-seq. Dataset71 (mouse hippocampus): seqFISH, https://ars.els-cdn.com/content/image/1-s2.0-S0896627316307024-mmc6.xlsx [79]; Drop-seq, GSE116470 in the GEO database. Dataset72 [mouse primary visual cortex (VISp)]: MERFISH, https://github.com/spacetx-spacejam/data/; Smart-seq, https://www.dropbox.com/s/dl/cuowvm4vrf65pvq/allen_cortex.rds. Dataset73 (olfactory bulb): Stereo-seq, https://db.cngb.org/stomics/mosta/ [58]; Smart-seq, GSE71585 in the GEO database [51]. Dataset74 (Zebrafish embryo): Stereo-seq, https://db.cngb.org/stomics/datasets/STDS0000057; Drop-seq, https://db.cngb.org/stomics/datasets/STDS0000057. Dataset75 (mouse brain): 10X Visium, https://github.com/BayraktarLab/cell2location; 10X Chromium, https://github.com/BayraktarLab/cell2location. Dataset76 (human PDAC): ST, GSE111672 in the GEO database [80]; inDrop, GSE111672 in the GEO database. Dataset77 (mouse brain): seqFISH, https://github.com/CaiGroup/seqFISH-PLUS; Drop-seq, GSE113576 in the GEO database [44]. The source code of CellMap is available at https://github.com/liuhong-jia/CellMap and https://zenodo.org/records/17240236. The benchmarking scripts are available at https://github.com/liuhong-jia/CellMap.Analysis.

## References

[1] Jovic D, Liang X, Zeng H et al. Single-cell RNA sequencing technologies and applications: a brief overview. Clinic Transl Med. 2022;12:e694. 10.1002/ctm2.694. PMC8964935 35352511

[2] Tian L, Chen F, Macosko EZ. The expanding vistas of spatial transcriptomics. Nat Biotechnol. 2023;41:773–82. 10.1038/s41587-022-01448-2. 36192637 PMC10091579

[3] Su M, Pan T, Chen QZ et al. Data analysis guidelines for single-cell RNA-seq in biomedical studies and clinical applications. Military Med Res. 2022;9:68. 10.1186/s40779-022-00434-8. PMC9716519 36461064

[4] Wang Y, Navin NE. Advances and applications of single-cell sequencing technologies. Mol Cell. 2015;58:598–609. 10.1016/j.molcel.2015.05.005. 26000845 PMC4441954

[5] Wu X, Liu Y, Jin S et al. Single-cell sequencing of immune cells from anticitrullinated peptide antibody positive and negative rheumatoid arthritis. Nat Commun. 2021;12:4977. 10.1038/s41467-021-25246-7. 34404786 PMC8371160

[6] Longo SK, Guo MG, Ji AL et al. Integrating single-cell and spatial transcriptomics to elucidate intercellular tissue dynamics. Nat Rev Genet. 2021;22:627–44. 10.1038/s41576-021-00370-8. 34145435 PMC9888017

[7] Rodriques SG, Stickels RR, Goeva A et al. Slide-seq: a scalable technology for measuring genome-wide expression at high spatial resolution. Science. 2019;363:1463–7. 10.1126/science.aaw1219. 30923225 PMC6927209

[8] Stickels RR, Murray E, Kumar P et al. Highly sensitive spatial transcriptomics at near-cellular resolution with Slide-seqV2. Nat Biotechnol. 2021;39:313–9. 10.1038/s41587-020-0739-1. 33288904 PMC8606189

[9] Rao A, Barkley D, França GS et al. Exploring tissue architecture using spatial transcriptomics. Nature. 2021;596:211–20. 10.1038/s41586-021-03634-9. 34381231 PMC8475179

[10] Park HE, Jo SH, Lee RH et al. Spatial transcriptomics: technical aspects of recent developments and their applications in neuroscience and cancer research. Adv Sci. 2023;10:e2206939. 10.1002/advs.202206939 PMC10238226 37026425

[11] Wang WJ, Chu LX, He LY et al. Spatial transcriptomics: recent developments and insights in respiratory research. Military Med Res. 2023;10:38. 10.1186/s40779-023-00471-x. PMC10433685 37592342

[12] Li B, Zhang W, Guo C et al. Benchmarking spatial and single-cell transcriptomics integration methods for transcript distribution prediction and cell type deconvolution. Nat Methods. 2022;19:662–70. 10.1038/s41592-022-01480-9. 35577954

[13] Eng C-HL, Lawson M, Zhu Q et al. Transcriptome-scale super-resolved imaging in tissues by RNA seqFISH+. Nature. 2019;568:235–9. 10.1038/s41586-019-1049-y. 30911168 PMC6544023

[14] Wang X, Allen WE, Wright MA et al. Three-dimensional intact-tissue sequencing of single-cell transcriptional states. Science. 2018;361:eaat5691. 10.1126/science.aat5691. 29930089 PMC6339868

[15] Moncada R, Barkley D, Wagner F et al. Integrating microarray-based spatial transcriptomics and single-cell RNA-seq reveals tissue architecture in pancreatic ductal adenocarcinomas. Nat Biotechnol. 2020;38:333–42. 10.1038/s41587-019-0392-8. 31932730

[16] Qi J, Sun H, Zhang Y et al. Single-cell and spatial analysis reveal interaction of FAP(+) fibroblasts and SPP1(+) macrophages in colorectal cancer. Nat Commun. 2022;13:1742. 10.1038/s41467-022-29366-6. 35365629 PMC8976074

[17] Wang F, Long J, Li L et al. Single-cell and spatial transcriptome analysis reveals the cellular heterogeneity of liver metastatic colorectal cancer. Sci Adv. 2023;9:eadf5464. 10.1126/sciadv.adf5464. 37327339 PMC10275599

[18] Yu X, Xie L, Ge J et al. Integrating single-cell RNA-seq and spatial transcriptomics reveals MDK-NCL dependent immunosuppressive environment in endometrial carcinoma. Front Immunol. 2023;14:1145300. 10.3389/fimmu.2023.1145300. 37081869 PMC10110842

[19] Jain S, Rick JW, Joshi RS et al. Single-cell RNA sequencing and spatial transcriptomics reveal cancer-associated fibroblasts in glioblastoma with protumoral effects. J Clin Invest. 2023;133:e147087:10.1172/JCI147087. 36856115 PMC9974099

[20] Elosua-Bayes M, Nieto P, Mereu E et al. SPOTlight: seeded NMF regression to deconvolute spatial transcriptomics spots with single-cell transcriptomes. Nucleic Acids Res. 2021;49:e50. 10.1093/nar/gkab043. 33544846 PMC8136778

[21] Dong R, Yuan GC. SpatialDWLS: accurate deconvolution of spatial transcriptomic data. Genome Biol. 2021;22:145. 10.1186/s13059-021-02362-7. 33971932 PMC8108367

[22] Cable DM, Murray E, Zou LS et al. Robust decomposition of cell type mixtures in spatial transcriptomics. Nat Biotechnol. 2022;40:517–26. 10.1038/s41587-021-00830-w. 33603203 PMC8606190

[23] Ma Y, Zhou X. Spatially informed cell-type deconvolution for spatial transcriptomics. Nat Biotechnol. 2022;40:1349–59. 10.1038/s41587-022-01273-7. 35501392 PMC9464662

[24] Kleshchevnikov V, Shmatko A, Dann E et al. Cell2location maps fine-grained cell types in spatial transcriptomics. Nat Biotechnol. 2022;40:661–71. 10.1038/s41587-021-01139-4. 35027729

[25] Lopez R, Li B, Keren-Shaul H et al. DestVI identifies continuums of cell types in spatial transcriptomics data. Nat Biotechnol. 2022;40:1360–9. 10.1038/s41587-022-01272-8. 35449415 PMC9756396

[26] Zhou Z, Zhong Y, Zhang Z et al. Spatial transcriptomics deconvolution at single-cell resolution using Redeconve. Nat Commun. 2023;14:7930. 10.1038/s41467-023-43600-9. 38040768 PMC10692090

[27] Danaher P, Kim Y, Nelson B et al. Advances in mixed cell deconvolution enable quantification of cell types in spatial transcriptomic data. Nat Commun. 2022;13:385. 10.1038/s41467-022-28020-5. 35046414 PMC8770643

[28] Andersson A, Bergenstråhle J, Asp M et al. Single-cell and spatial transcriptomics enables probabilistic inference of cell type topography. Commun Biol. 2020;3:565. 10.1038/s42003-020-01247-y. 33037292 PMC7547664

[29] Wan X, Xiao J, Tam SST et al. Integrating spatial and single-cell transcriptomics data using deep generative models with SpatialScope. Nat Commun. 2023;14:7848. 10.1038/s41467-023-43629-w. 38030617 PMC10687049

[30] Zhang Q, Jiang S, Schroeder A et al. Leveraging spatial transcriptomics data to recover cell locations in single-cell RNA-seq with CeLEry. Nat Commun. 2023;14:4050. 10.1038/s41467-023-39895-3. 37422469 PMC10329686

[31] Li H, Zhou J, Li Z et al. A comprehensive benchmarking with practical guidelines for cellular deconvolution of spatial transcriptomics. Nat Commun. 2023;14:1548. 10.1038/s41467-023-37168-7. 36941264 PMC10027878

[32] Stuart T, Butler A, Hoffman P et al. Comprehensive integration of single-cell data. Cell. 2019;177:1888–902. 10.1016/j.cell.2019.05.031. 31178118 PMC6687398

[33] Korsunsky I, Millard N, Fan J et al. Fast, sensitive and accurate integration of single-cell data with Harmony. Nat Methods. 2019;16:1289–96. 10.1038/s41592-019-0619-0. 31740819 PMC6884693

[34] Wei R, He S, Bai S et al. Spatial charting of single-cell transcriptomes in tissues. Nat Biotechnol. 2022;40:1190–9. 10.1038/s41587-022-01233-1. 35314812 PMC9673606

[35] Biancalani T, Scalia G, Buffoni L et al. Deep learning and alignment of spatially resolved single-cell transcriptomes with Tangram. Nat Methods. 2021;18:1352–62. 10.1038/s41592-021-01264-7. 34711971 PMC8566243

[36] Vahid MR, Brown EL, Steen CB et al. High-resolution alignment of single-cell and spatial transcriptomes with CytoSPACE. Nat Biotechnol. 2023;41:1543–8. 10.1038/s41587-023-01697-9. 36879008 PMC10635828

[37] Moriel N, Senel E, Friedman N et al. NovoSpaRc: flexible spatial reconstruction of single-cell gene expression with optimal transport. Nat Protoc. 2021;16:4177–200. 10.1038/s41596-021-00573-7. 34349282

[38] Cang Z, Nie Q. Inferring spatial and signaling relationships between cells from single cell transcriptomic data. Nat Commun. 2020;11:2084. 10.1038/s41467-020-15968-5. 32350282 PMC7190659

[39] Liu H, Li H, Sharma A et al. scAnno: a deconvolution strategy-based automatic cell type annotation tool for single-cell RNA-sequencing data sets. Brief Bioinform. 2023;24:bbad179. 10.1093/bib/bbad179. 37183449

[40] Jonker R, Volgenant A. A shortest augmenting path algorithm for dense and sparse linear assignment problems. Computing. 1987;38:325–40. 10.1007/BF02278710.

[41] Sun D, Liu Z, Li T et al. STRIDE: accurately decomposing and integrating spatial transcriptomics using single-cell RNA sequencing. Nucleic Acids Res. 2022;50:e42. 10.1093/nar/gkac150. 35253896 PMC9023289

[42] Lin J . Divergence measures based on the Shannon entropy. IEEE Trans Inf Theor. 1991;37:145–51. 10.1109/18.61115.

[43] Moriel N, Senel E, Friedman N et al. NovoSpaRc: flexible spatial reconstruction of single-cell gene expression with optimal transport. Nat Protoc. 2021;16:4177–200. 10.1038/s41596-021-00573-7. 34349282

[44] Moffitt JR, Bambah-Mukku D, Eichhorn SW et al. Molecular, spatial, and functional single-cell profiling of the hypothalamic preoptic region. Science. 2018;362:eaau5324:10.1126/science.aau5324. 30385464 PMC6482113

[45] Kim Y, Cheng W, Cho C-S et al. Seq-Scope: repurposing Illumina sequencing flow cells for high-resolution spatial transcriptomics. Nat Protoc. 2025;20:643–89. 10.1038/s41596-024-01065-0. 39482362 PMC11896753

[46] Wang X, Allen WE, Wright MA et al. Three-dimensional intact-tissue sequencing of single-cell transcriptional states. Science. 2018;361:eaat5691. 10.1126/science.aat5691. 29930089 PMC6339868

[47] Kukanja P, Langseth CM, Rubio Rodríguez-Kirby LA et al. Cellular architecture of evolving neuroinflammatory lesions and multiple sclerosis pathology. Cell. 2024;187:1990–2009. 10.1016/j.cell.2024.02.030. 38513664

[48] Sautès-Fridman C, Petitprez F, Calderaro J et al. Tertiary lymphoid structures in the era of cancer immunotherapy. Nat Rev Cancer. 2019;19:307–25. 10.1038/s41568-019-0144-6. 31092904

[49] Cabrita R, Lauss M, Sanna A et al. Tertiary lymphoid structures improve immunotherapy and survival in melanoma. Nature. 2020;577:561–5. 10.1038/s41586-019-1914-8. 31942071

[50] Helmink BA, Reddy SM, Gao J et al. B cells and tertiary lymphoid structures promote immunotherapy response. Nature. 2020;577:549–55. 10.1038/s41586-019-1922-8. 31942075 PMC8762581

[51] Tasic B, Menon V, Nguyen TN et al. Adult mouse cortical cell taxonomy revealed by single cell transcriptomics. Nat Neurosci. 2016;19:335–46. 10.1038/nn.4216. 26727548 PMC4985242

[52] Wu SZ, Al-Eryani G, Roden DL et al. A single-cell and spatially resolved atlas of human breast cancers. Nat Genet. 2021;53:1334–47. 10.1038/s41588-021-00911-1. 34493872 PMC9044823

[53] Melo Ferreira R, Sabo AR, Winfree S et al. Integration of spatial and single-cell transcriptomics localizes epithelial cell-immune cross-talk in kidney injury. JCI Insight. 2021;6:e147703. 10.1172/jci.insight.147703. 34003797 PMC8262485

[54] Ransick A, Lindström NO, Liu J et al. Single-cell profiling reveals sex, lineage, and regional diversity in the mouse kidney. Dev Cell. 2019;51:399–413. 10.1016/j.devcel.2019.10.005. 31689386 PMC6948019

[55] Meylan M, Petitprez F, Becht E et al. Tertiary lymphoid structures generate and propagate anti-tumor antibody-producing plasma cells in renal cell cancer. Immunity. 2022;55:527–41. 10.1016/j.immuni.2022.02.001. 35231421

[56] Stickels RR, Murray E, Kumar P et al. Highly sensitive spatial transcriptomics at near-cellular resolution with Slide-seqV2. Nat Biotechnol. 2021;39:313–9. 10.1038/s41587-020-0739-1. 33288904 PMC8606189

[57] Saunders A, Macosko EZ, Wysoker A et al. Molecular diversity and specializations among the cells of the adult mouse brain. Cell. 2018;174:1015–30. 10.1016/j.cell.2018.07.028. 30096299 PMC6447408

[58] Chen A, Liao S, Cheng M et al. Spatiotemporal transcriptomic atlas of mouse organogenesis using DNA nanoball-patterned arrays. Cell. 2022;185:1777–92. 10.1016/j.cell.2022.04.003. 35512705

[59] Di Bella DJ, Habibi E, Stickels RR et al. Molecular logic of cellular diversification in the mouse cerebral cortex. Nature. 2021;595:554–9. 10.1038/s41586-021-03670-5. 34163074 PMC9006333

[60] McKellar DW, Walter LD, Song LT et al. Large-scale integration of single-cell transcriptomic data captures transitional progenitor states in mouse skeletal muscle regeneration. Communications Biology. 2021;4:1280. 10.1038/s42003-021-02810-x. 34773081 PMC8589952

[61] Wu SZ, Al-Eryani G, Roden DL et al. A single-cell and spatially resolved atlas of human breast cancers. Nat Genet. 2021;53:1334–47. 10.1038/s41588-021-00911-1. 34493872 PMC9044823

[62] Sanchez-Ferras O, Pacis A, Sotiropoulou M et al. A coordinated progression of progenitor cell states initiates urinary tract development. Nat Commun. 2021;12:2627. 10.1038/s41467-021-22931-5. 33976190 PMC8113267

[63] McCray T, Pacheco JV, Loitz CC et al. Vitamin D sufficiency enhances differentiation of patient-derived prostate epithelial organoids. iScience. 2021;24:101974. 10.1016/j.isci.2020.101974. 33458620 PMC7797919

[64] Ratz M, von Berlin L, Larsson L et al. Clonal relations in the mouse brain revealed by single-cell and spatial transcriptomics. Nat Neurosci. 2022;25:285–94. 10.1038/s41593-022-01011-x. 35210624 PMC8904259

[65] Joglekar A, Prjibelski A, Mahfouz A et al. A spatially resolved brain region- and cell type-specific isoform atlas of the postnatal mouse brain. Nat Commun. 2021;12:463. 10.1038/s41467-020-20343-5. 33469025 PMC7815907

[66] Janosevic D, Myslinski J, McCarthy TW et al. The orchestrated cellular and molecular responses of the kidney to endotoxin define a precise sepsis timeline. eLife. 2021;10:e62270. 10.7554/eLife.62270. 33448928 PMC7810465

[67] Lopez R, Li B, Keren-Shaul H et al. DestVI identifies continuums of cell types in spatial transcriptomics data. Nature Biotechnology. 2022;40:1360–9. 10.1038/s41587-022-01272-8. PMC9756396 35449415

[68] Biancalani T, Scalia G, Buffoni L et al. Deep learning and alignment of spatially resolved single-cell transcriptomes with Tangram. Nat Methods. 2021;18:1352–62. 10.1038/s41592-021-01264-7. 34711971 PMC8566243

[69] Ji AL, Rubin AJ, Thrane K et al. Multimodal analysis of composition and spatial architecture in human squamous cell carcinoma. Cell. 2020;182:497–514. 10.1016/j.cell.2020.05.039. 32579974 PMC7391009

[70] Navarro JF, Croteau DL, Jurek A et al. Spatial transcriptomics reveals genes associated with dysregulated mitochondrial functions and stress signaling in alzheimer disease. iScience. 2020;23:101556. 10.1016/j.isci.2020.101556. 33083725 PMC7522123

[71] Brann DH, Tsukahara T, Weinreb C et al. Non-neuronal expression of SARS-CoV-2 entry genes in the olfactory system suggests mechanisms underlying COVID-19-associated anosmia. Sci Adv. 2020;6:10.1126/sciadv.abc5801. PMC10715684 32937591

[72] Xia C, Fan J, Emanuel G et al. Spatial transcriptome profiling by MERFISH reveals subcellular RNA compartmentalization and cell cycle-dependent gene expression. Proc Natl Acad Sci USA. 2019;116:19490–9. 31501331 10.1073/pnas.1912459116 PMC6765259

[73] Zhou Y, Yang D, Yang Q et al. Single-cell RNA landscape of intratumoral heterogeneity and immunosuppressive microenvironment in advanced osteosarcoma. Nat Commun. 2020;11:6322. 10.1038/s41467-020-20059-6. 33303760 PMC7730477

[74] Hunter MV, Moncada R, Weiss JM et al. Spatially resolved transcriptomics reveals the architecture of the tumor-microenvironment interface. Nat Commun. 2021;12:6278. 10.1038/s41467-021-26614-z. 34725363 PMC8560802

[75] Cho CS, Xi J, Si Y et al. Microscopic examination of spatial transcriptome using Seq-Scope. Cell. 2021;184:3559–72. 10.1016/j.cell.2021.05.010. 34115981 PMC8238917

[76] Tabula Muris Consortium . Single-cell transcriptomics of 20 mouse organs creates a *Tabula Muris*. Nature. 2018;562:367–72. 10.1038/s41586-018-0590-4. 30283141 PMC6642641

[77] Lohoff T, Ghazanfar S, Missarova A et al. Integration of spatial and single-cell transcriptomic data elucidates mouse organogenesis. Nat Biotechnol. 2022;40:74–85. 10.1038/s41587-021-01006-2. 34489600 PMC8763645

[78] Hodge RD, Bakken TE, Miller JA et al. Conserved cell types with divergent features in human versus mouse cortex. Nature. 2019;573:61–8. 10.1038/s41586-019-1506-7. 31435019 PMC6919571

[79] Shah S, Lubeck E, Zhou W et al. *In situ* transcription profiling of single cells reveals spatial organization of cells in the mouse hippocampus. Neuron. 2016;92:342–57. 10.1016/j.neuron.2016.10.001. 27764670 PMC5087994

[80] Rosenbluth JM, Mays DJ, Pino MF et al. A gene signature-based approach identifies mTOR as a regulator of p73. Mol Cell Biol. 2008;28:5951–64. 10.1128/MCB.00305-08. 18678646 PMC2547001

