# Supplementary material for: CellMap: precision mapping of cellular landscape in spatial transcriptomics
Source: Nucleic Acids Res. 2026 Jan 8;54(1):gkaf1484. doi: 10.1093/nar/gkaf1484 (PMC12781899; doi:10.1093/nar/gkaf1484)
Supplement: gkaf1484_Supplemental_Files [file gkaf1484_supplemental_files.zip › Supplementary data.pdf]

Supplementary Information

Supplementary Methods

PCA

Transposed the normalized expression matrix to obtain matrix O, $O = z^T$, $O \in \mathrm{R}^{k \times m}$, Let $O = [o_1; o_2; \cdots; o_m]$, where o_i is a vector of expression of each gene in cells, and m is the number of genes; $\bar{o}$ is mean expression of all genes. After normalizing the vectors to unity norm, $o'_i = o_i - \bar{o}$, the covariance matrix of $O' = [o'_1; o'_2; \cdots; o'_m]$ was calculated as: $\mathrm{C} = \frac{1}{m}(O')^T O'$, and the eigenvector Φ and eigenvalues Λ were computed as $\mathrm{C}\Phi = \Phi\Lambda$, where $\Phi = [\Phi_1, \Phi_2, \cdots, \Phi_m]$ and $\Lambda = \mathrm{diag}\{\lambda_1, \lambda_2, \cdots, \lambda_m\}$ (sorted by size, λ is eigenvalues of matrix), the eigenvectors were arranged into a matrix from top to bottom in rows according to the corresponding eigenvalues, and took the first t rows to form the matrix P. $\mathrm{P} = [\Phi_1, \Phi_2, \cdots, \Phi_t]^T$, $\mathrm{P} \in \mathrm{R}^{t \times k}$. The coordinate transformation of the normalized expression matrix z was performed by the transformation matrix P, which obtained the dimensionality reduction signal matrix Q. $Q = P \cdot O$, $Q \in \mathrm{R}^{t \times m}$. We choose the number of Principal Components (PCs) t to be twice the number of cell types in the reference expression matrix by default.

Performance comparison with available methods

CellMap

CellMap is a novel computational method that allows spatial transcriptomic spots to be resolved at single-cell resolution. For high-resolution data, we set knn = 1 and mean.cell.num = 1, while for low-resolution data, we set knn = 5 and mean.cell.num = 5.

CytoSPACE

CytoSPACE [1] is a novel computational method for assigning single-cell transcriptomes to in situ spatial transcriptomics data. We followed the guidelines provided on the GitHub repository of CytoSPACE: https://github.com/digitalcytometry/cytospace and we used the recommended default parameter settings for both low-resolution and high-resolution ST data.

CellTrek

CellTrek [2] is a computational framework that can directly map single cells back to their spatial coordinates in tissue sections based on scRNA-seq and spatial transcriptomics data. We followed the guidelines provided on the GitHub repository of CellTrek: https://github.com/navinlabcode/CellTrek. We ran *celltrek* function with default parameters (intp_pnt = 5000, intp_lin = F, nPCs = 30, ntree = 1000, dist_thresh = 0.55, top_spot = 5, spot_n = 5, repel_r = 20, repel_iter = 20, keep_model = T).

Tangram

Tangram [3] is a deep-learning framework for mapping single-cell (or single-nucleus) gene expression data onto spatial gene expression data. We followed the instructions provided on the GitHub repository of Tangram: https://github.com/broadinstitute/Tangram. To visualize the mapping of single cells onto spatial sections, random coordinates were generated for individual cells based on the spatial coordinates derived from ST data.

RCTD

RCTD [4] is a computational method that uses cell type profiles extracted from single-cell RNA-seq to dissect cell type mixtures. We followed the tutorial provided on the GitHub repository of

RCTD: https://raw.githack.com/dmcable/RCTD/master/vignettes/spatial-transcriptomics.html. We ran RCTD with doublet_mode = 'full'.

CARD

CARD [5] is a deconvolution method that combines cell-type-specific expression information from scRNA-seq with correlation in cell-type composition across tissue locations. Information about the CARD GitHub repository was obtained via web link: https://yma-lab.github.io/CARD/documentation/04_CARD_Example.html.

Cell2location

Cell2location [6] is a Bayesian model designed to resolve fine-grained cell types within spatial transcriptomic data, enabling the construction of comprehensive cellular maps of diverse tissues. We followed the tutorial on the Github repository of Cell2location: https://cell2location.readthedocs.io/en/latest/notebooks/cell2location_tutorial.html.

DestVI

DestVI [7] is a probabilistic method for the deconvolution of spatial transcriptomics profiles that enables multi-resolution analysis and models continuous variation within cell types. We followed the tutorial on DestVI website: https://docs.scvi-tools.org/en/stable/tutorials/notebooks/spatial/DestVI_tutorial.html. The single-cell model was trained with parameters max_epochs = 50, lr = 0.001, number of training genes = 2000. The spatial transcriptome model was trained with parameter max_epochs = 100.

NovoSpaRc

NovoSpaRc [8] is a computational framework that predicts locations of single cells in space by solely using scRNA-seq data. We followed the tutorial on the Github repository of NovoSpaRc: https://github.com/rajewsky-lab/novosparc/blob/master/reconstruct_drosophila_embryo_tutorial.ipynb.

Redeconve

Redeconve [9] is an algorithm designed to deconvolute spatial transcriptomics data at single-cell resolution, enabling interpretation of spatial transcriptomics data. We followed the manual provided on the Github repository of Redeconve: https://zxzhou4150.github.io/Redeconve%20manual.html.

SpatialDWLS

SpatialDWLS [10] is a method for quantitatively estimating cell-type composition at each spatial location. We followed the guidelines on the SpatialDWLS website: https://rubd.github.io/Giotto_site/articles/tut7_giotto_enrichment.html. For low-resolution data, we set n_cell = 5, and for high-resolution data, we set n_cell = 2.

SPOTlight

SPOTlight [11] is a computational tool that enables the integration of ST with scRNA-seq data， enabling precise inference of cell types and states within complex tissues. We followed the guidelines available on the Github repository of SPOTlight ： https://github.com/MarcElosua/SPOTlight.

Stereoscope

Stereoscope [12] is a model-based probabilistic method that uses single cell data to deconvolve the cell mixtures in spatial data. We followed the guidelines available on the Github repository of Stereoscope: https://github.com/almaan/stereoscope.

Seurat

Seurat [13] utilizes a data transfer framework to map spatial transcriptomics (ST) coordinates to individual cell types, treating each spot as a unit at single-cell resolution. In this study, the *FindTransferAnchors* function was applied with ST data as the query and the corresponding single-cell data as the reference. Subsequently, the *TransferData* function was employed to assign single-cell labels (i.e., cell types) to the ST spots. Analyses were performed using the default parameters as specified in the official documentation.

SpaOTsc

SpaOTsc [14] is a method that leverages structured optimal transport to infer the spatial organization of scRNA-seq data using spatial measurements of a relatively small number of genes. We followed the manual provided on the Github repository of SpaOTsc: https://github.com/zcang/SpaOTsc.

SpatialDecon

SpatialDecon [15] is an algorithm designed to quantify cell populations defined by single cell sequencing within the regions of spatial gene expression studies. We followed the guidelines provided on the Github repository of SpatialDecon: https://github.com/Nanostring-Biostats/SpatialDecon/.

Statistical analysis

The statistical analysis was performed using R version 4.1.1. Pearson correlation analysis, z-test, Student's *t*-test, Kruskal-Wallis test were performed. The *p*-values < 0.05 were considered statistically significant.

Supplementary Figures

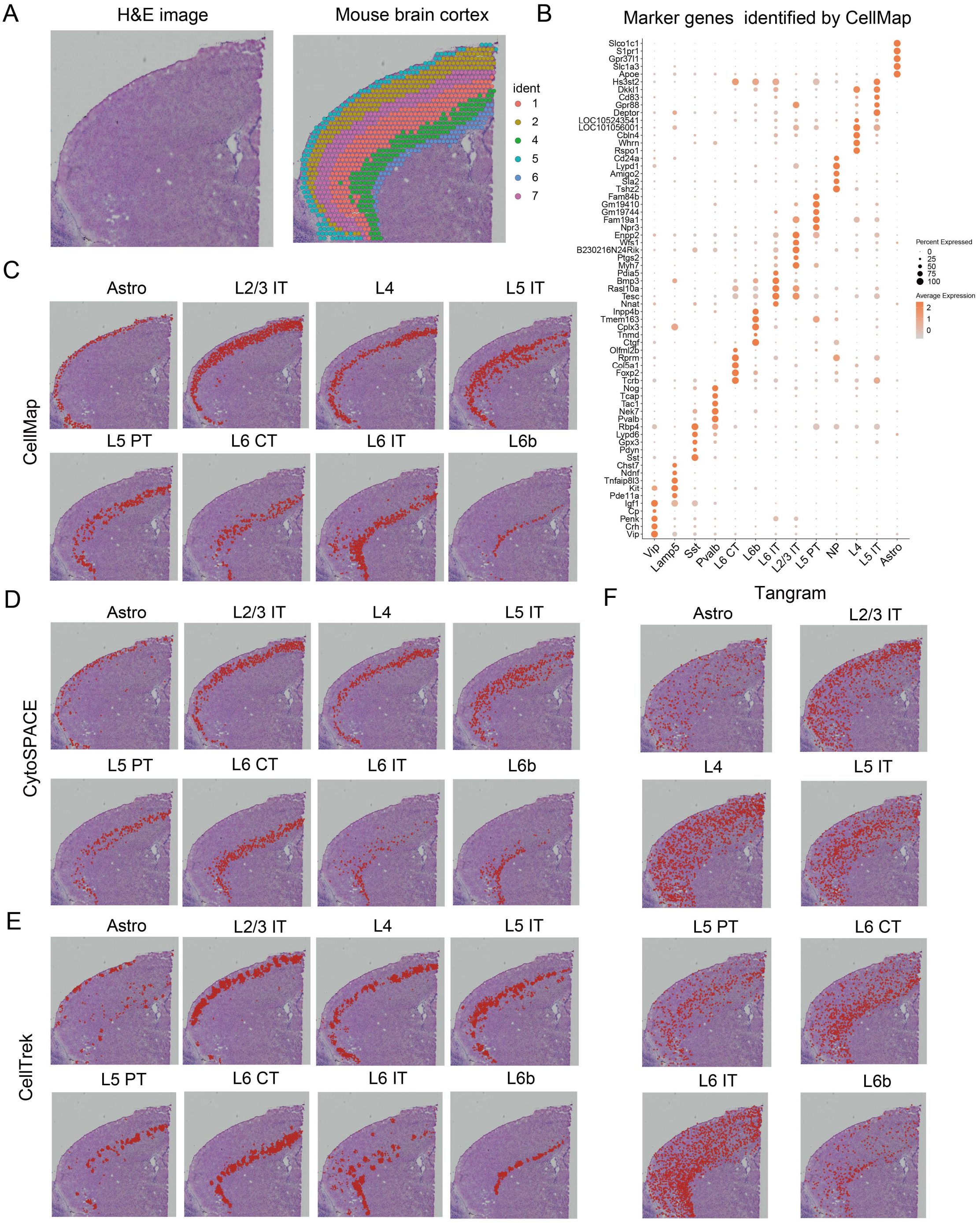

Figure S1. The spatial profiling and cell-type specific genes in mouse cerebral cortex tissue. (A) Left: Hematoxylin and eosin (H&E) staining image of mouse cerebral cortex. Right: Spatial spot clustering of mouse cerebral cortex tissue slice using the Seurat standard pipeline. (B) Bubble plot depicting cell-type specific genes identified by CellMap, where the intensity of color represents the gene expression strength, and dot size represents the percentage of genes expressed in the cell type. (C-F) The spatial distribution of selected cell types in the mouse cerebral cortex region depicted by four mapping methods: (C) CellMap; (D) CytoSPACE; (E) CellTrek; (F) Tangram.

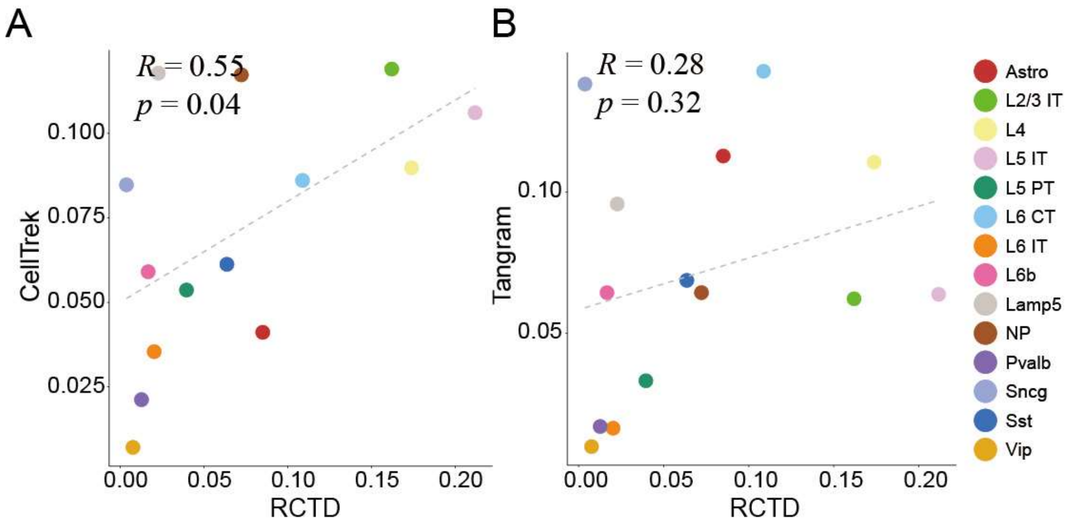

Figure S2. Performance assessment of cellular proportions predicted by CellTrek and Tangram in mouse cerebral cortex ST dataset. Scatter plots depicting the consistency between cellular compositions in spatial single-cells maps reconstructed using CellTrek (A) and Tangram (B), and cellular compositions predicted by RCTD spatial deconvolution method. "*R*" represents the Pearson correlation coefficients and the p-values were obtained by t-test.

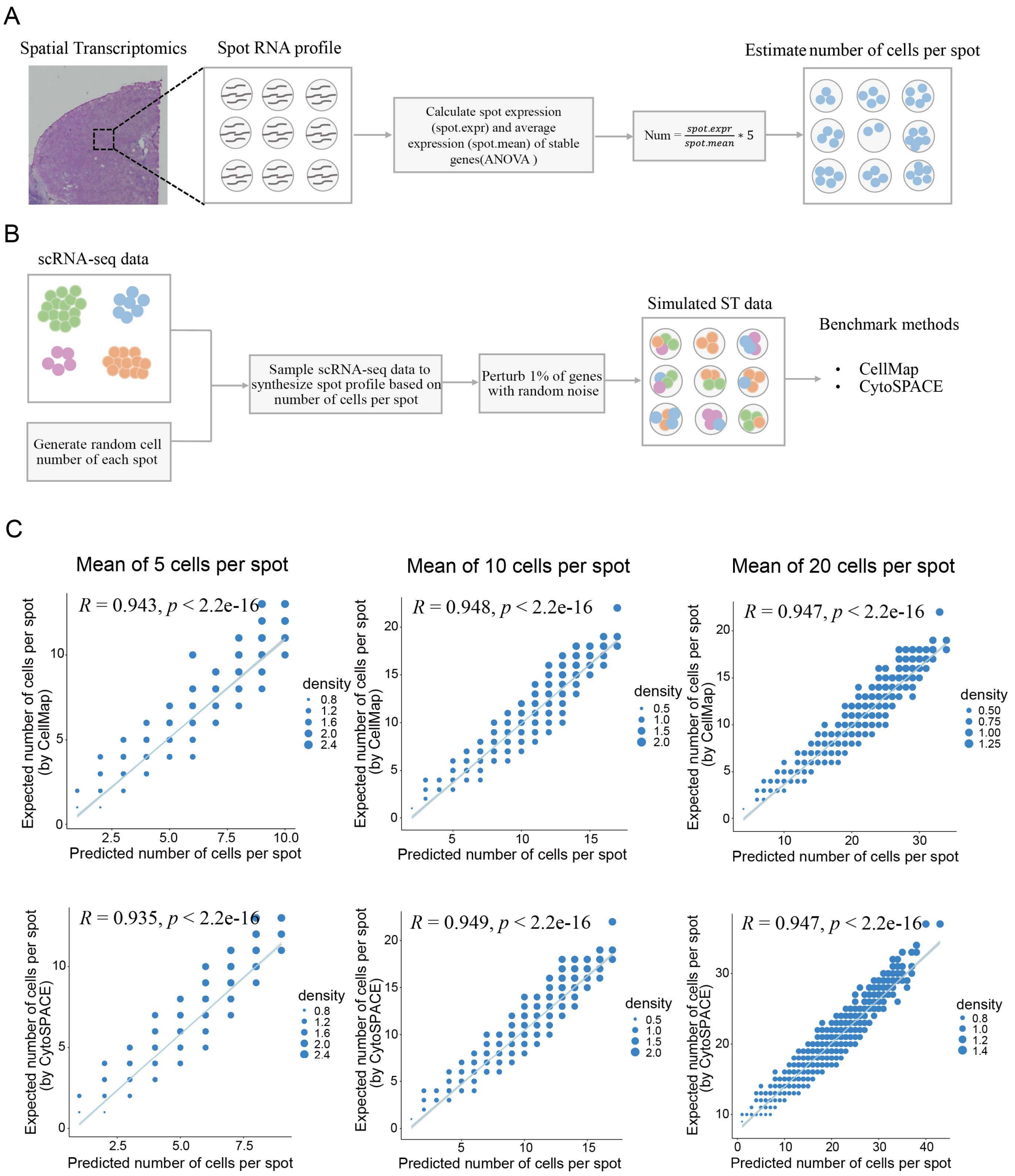

Figure S3. Performance evaluation of CellMap in estimating spot cell counts based on simulated spatial transcriptomic datasets. (A) Procedure for estimating the number of single cells in spots (see Materials and Methods). (B) The strategy for generating synthetic simulated ST (spatial transcriptomic) dataset consists primary of three steps: (1) Generate random cell counts for each spot based on Poisson distribution. (2) Randomly sampling a given number of single cells and aggregating the gene expression of these single cells to create a spot profile. (3) Adding a 1% transcriptional perturbation to the spot profile to generate simulated ST data (see Materials and Methods). (C) Scatter plots depicting the consistency between the predicted and expected number of cells based on simulated ST data with an average cell counts of 5, 10 and 20. (Upper panel) CellMap; (Lower panel) CytoSPACE. The blue line represents linear fit. "*R*" represents the Pearson correlation coefficients and the *p*-values were obtained by t-test.

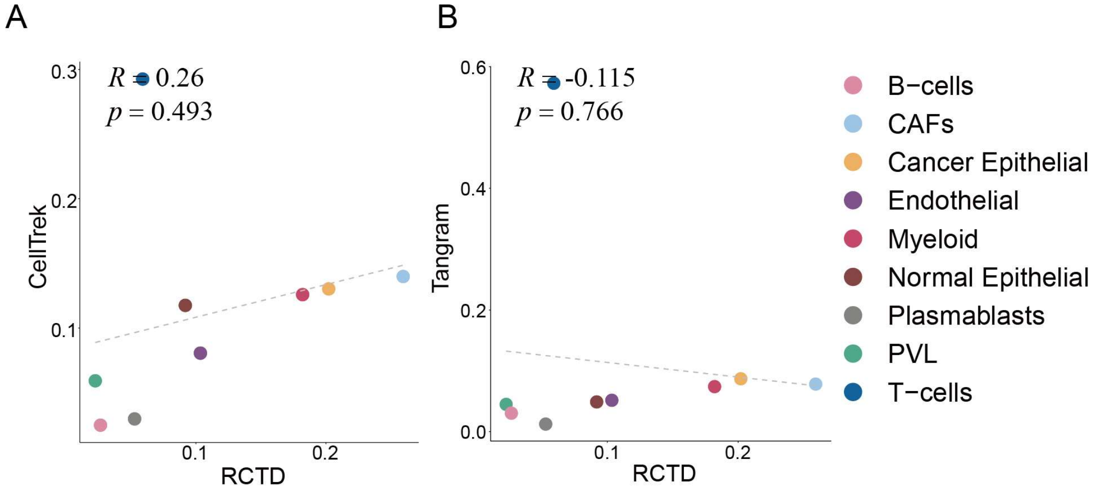

Figure S4. Performance assessment of cellular proportions predicted by CellTrek and Tangram in HER2+ breast cancer ST dataset. Scatter plots depicting the consistency between cellular compositions in spatial single-cells maps reconstructed using CellTrek (A) and Tangram (B), and cellular compositions predicted by RCTD spatial deconvolution method. "*R*" represents the Pearson correlation coefficients and the p-values were obtained by t-test.

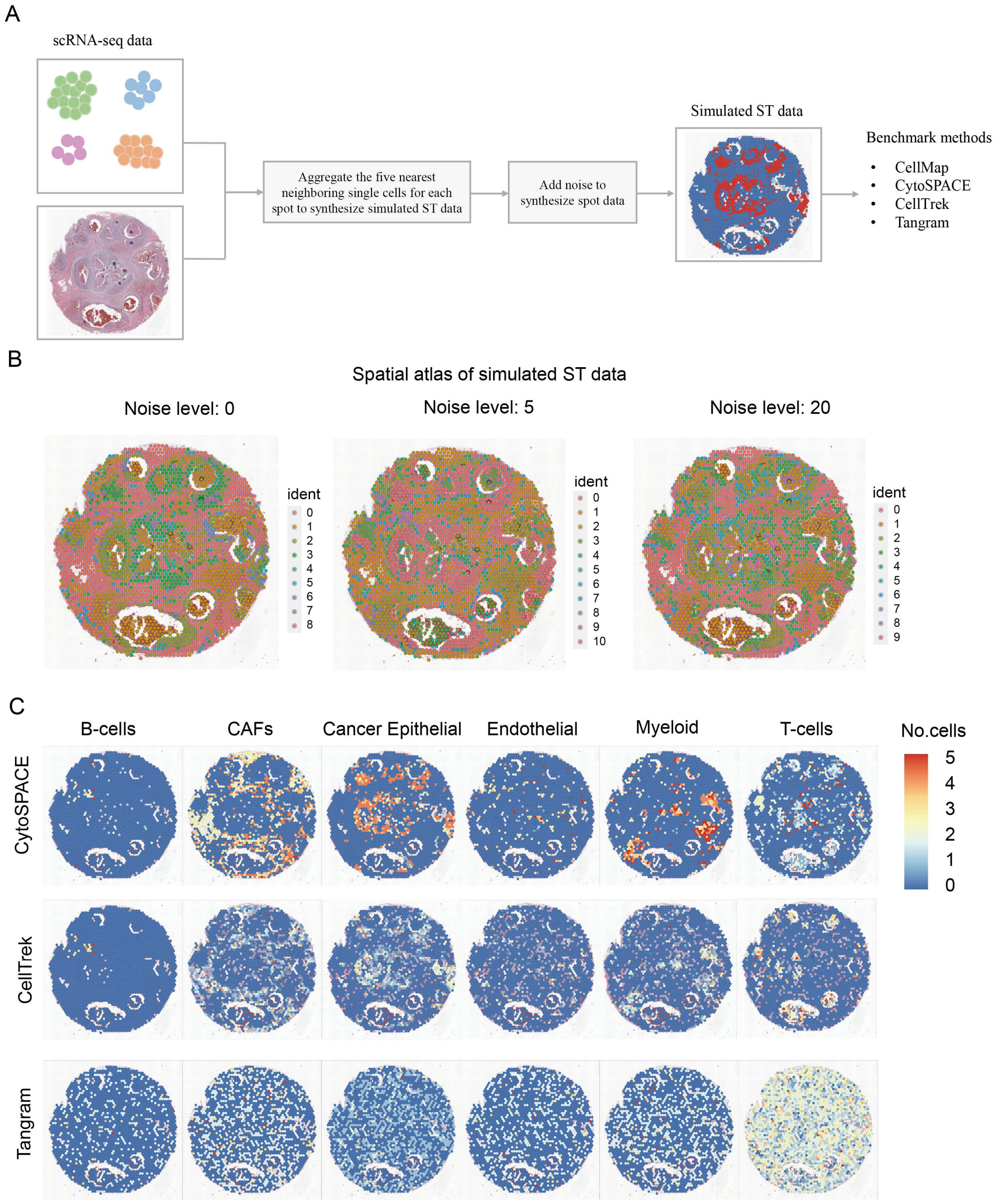

Figure S5. Simulated spatial transcriptomics data generation and performance evaluation. (A) The strategy for generating synthetic simulated ST datasets consists primary of three steps: (1) Integrate SC (single-cell) and ST (spatial transcriptomic) data to obtain integrated data, and UMAP is used to generate a two-dimensional projection of the integrated data. (2) Calculate the Euclidean distances between single cells and spots, obtain the 5 nearest neighbor single cells for each spot. Aggregate the gene expression of the neighboring single cells corresponding to each spot. (3) Random permutations of k% of genes are applied to the synthesized spot expression profiles to generate synthetic ST data with diverse noise levels (see Materials and Methods). (B) Spatial spot clustering of synthetic ST data with diverse noise levels (0%, 5% and 20%) on HER2+ breast cancer FFPE section. (C) Spatial heat maps depicting the performance of publicly available mapping methods (CytoSPACE, CellTrek and

Tangram) for aligning scRNA-seq data (with 5% added noise) to spatial locations in simulated ST dataset with each spot containing an average of five cells (see Materials and Methods). For clarity, only cell types with distinct spatial structures are shown. The color intensity of individual spots indicates the number of mapped single cells.

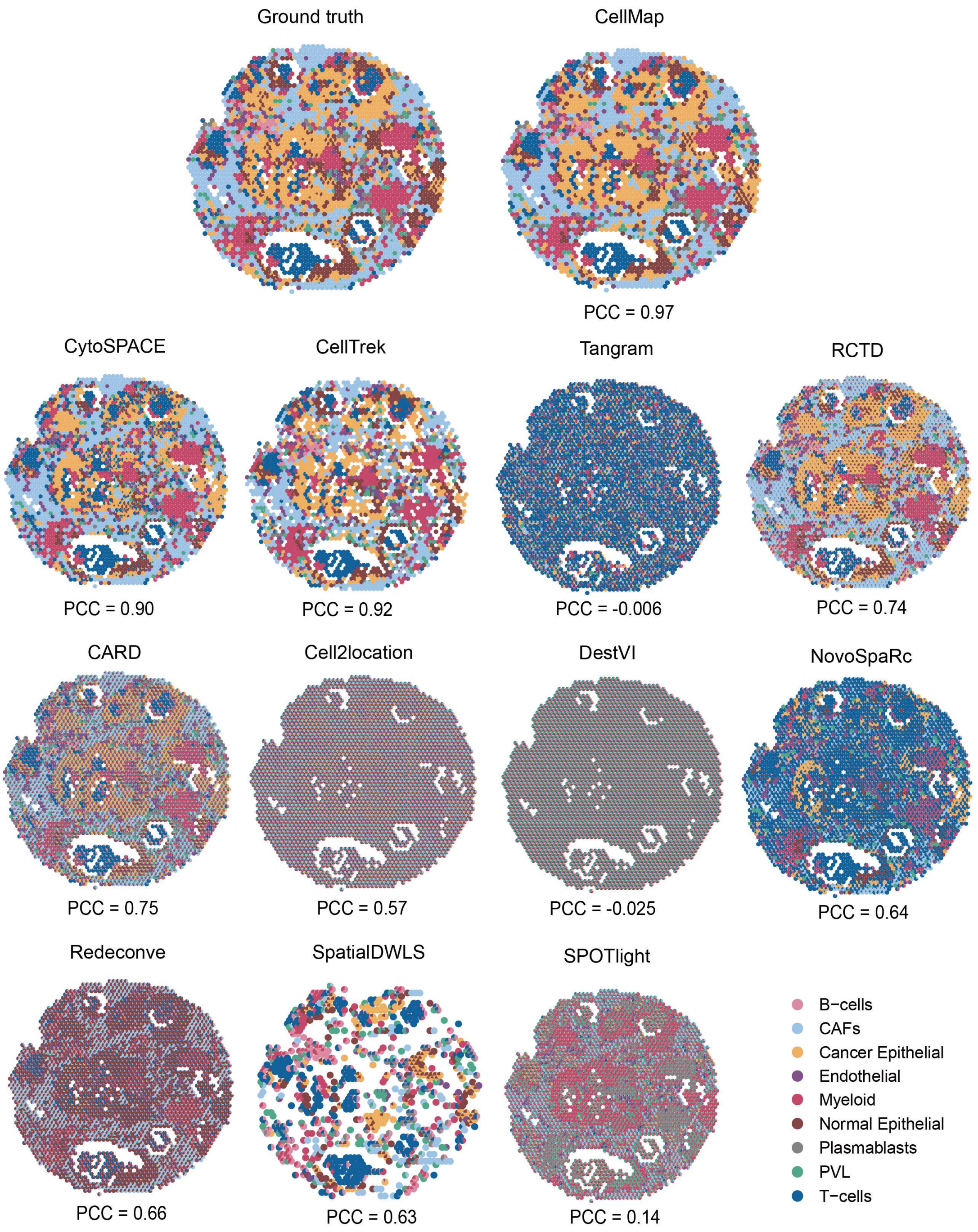

Figure S6. Performance assessment of 12 methods in simulated ST dataset. The proportions of nine cell types in spots from the simulated ST dataset (with 0% added noise), including the ground truth and the predicted results from 12 methods.

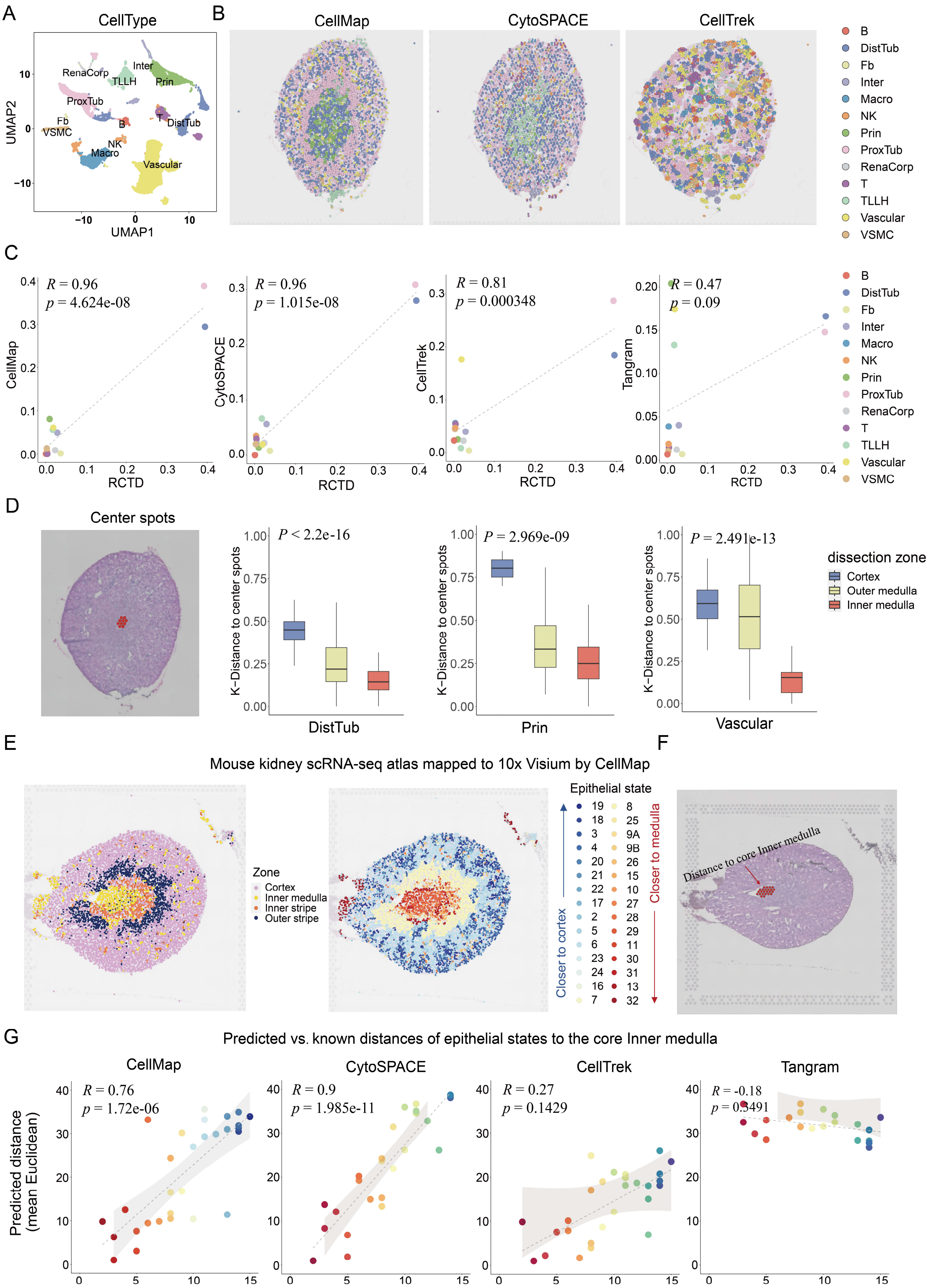

Figure S7. Performance assessment of CellMap on mouse kidney tissue. (A) The UMAP layout depicted the clustering space of mouse kidney scRNA-seq data. (B) Spatial structure of mouse kidney tissue reconstructed using CellMap，CytoSPACE and CellTrek. The cell types are color-coded, with each dot representing an individual cell. (C) Scatter plots demonstrate the consistency between cellular compositions in spatial single-cells maps reconstructed using four mapping methods and the cellular compositions predicted by RCTD spatial deconvolution method. “*R*” represents the PCCs (Pearson correlation coefficients) and the p-values were

obtained by t-test. (D) The left panel displays center spots as reference. From the spatial single-cells map reconstructed by CellMap, the spatial K-distances of DisTub, Prin and Vascular cells to the center spots across experimental zonal dissections (right). The boxplots display the median and quartile ranges (25-75%), with whiskers extending to 1.5 times the interquartile range from the box. p-values were obtained through the Kruskal–Wallis rank sum test. (E) Left: Transcriptome data of epithelial cells from mouse kidney scRNA-seq data mapped to mouse kidney ST sections using CellMap. Right: The same depiction as above, with cells are colored according to their known proximity to the inner medulla. (F) The panel displays the core Inner medulla as reference. (G) Scatter plots showing the consistency between the predicted and known distances of each epithelial state to the base of the inner medulla. Different colored dots represent distinct epithelial states. The dashed line represents linear fit and the shaded area indicates the 95% confidence interval. "*R*" represents the PCCs, the p-values were obtained by t-test.

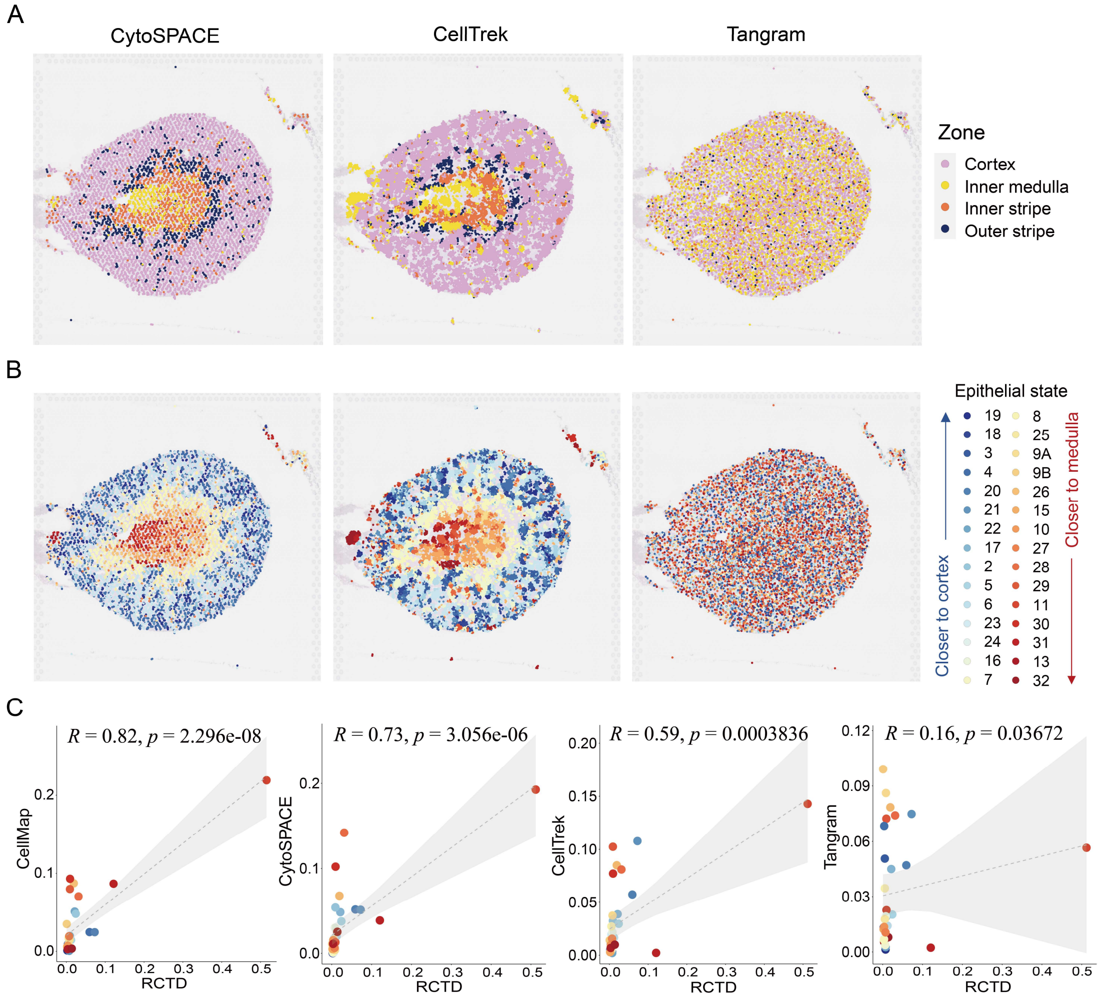

Figure S8. Investigating the spatial structure of mouse kidney tissue. (A-B) Upper: Transcriptome data of epithelial cells from mouse kidney scRNA-seq data mapped to mouse kidney ST sections using publicly available mapping methods (CytoSPACE, CellTrek and Tangram). Lower: The same depiction as above, with cells are colored according to their known proximity to the inner medulla. (C) Scatter plots demonstrate the consistency between cellular compositions in spatial single-cell maps reconstructed using four mapping methods and the cellular compositions predicted by RCTD spatial deconvolution method. Different colored dots represent distinct epithelial states. The dashed line represents linear fit, and the shaded area indicates the 95% confidence interval. “*R*” represents the Pearson correlation coefficients, the *p*-values were obtained by t-test.

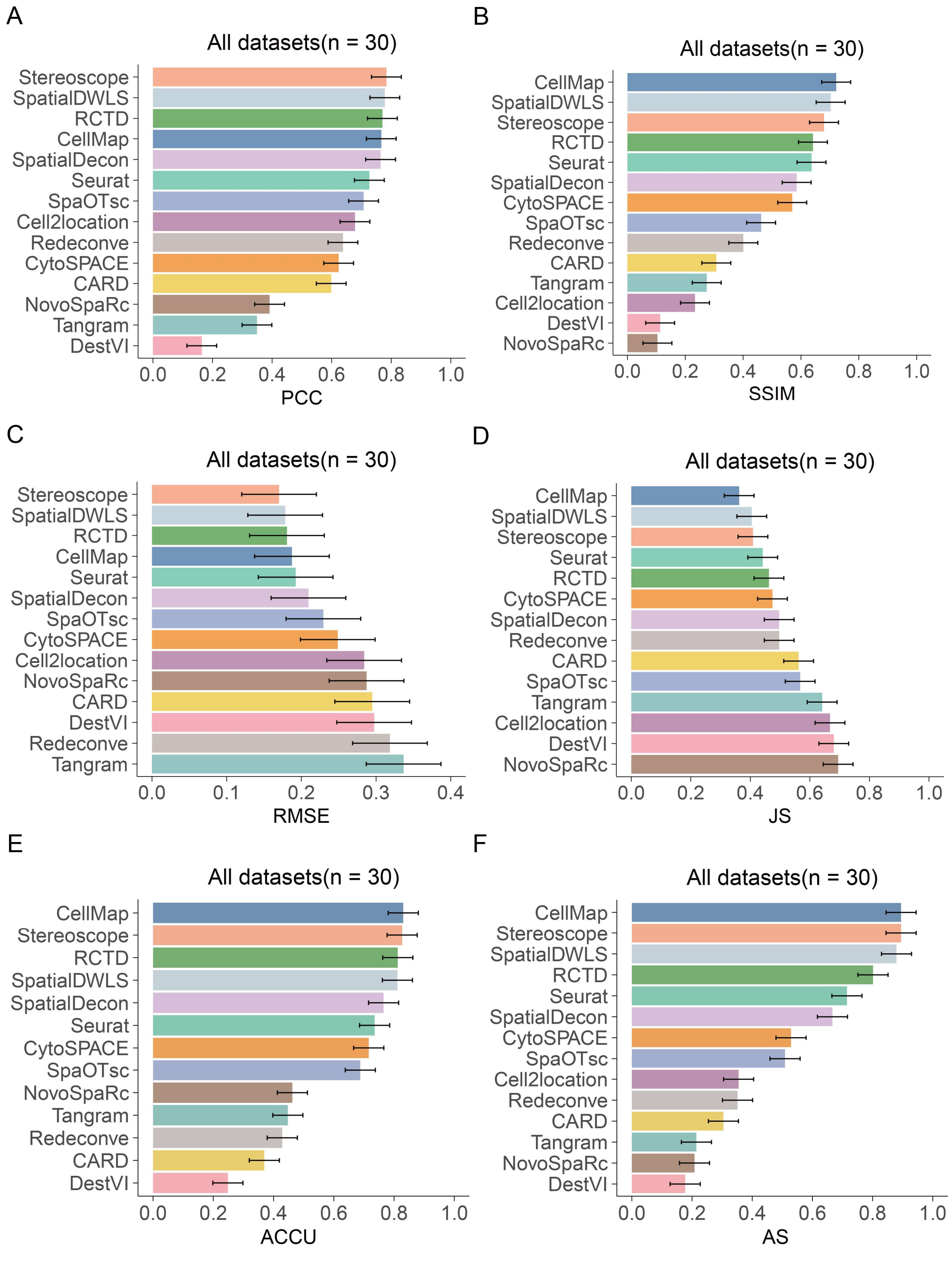

Figure S9. Comparing the accuracy of 14 integration methods for resolving spatial cell type distributions. (A-E) Average PCC, SSIM, RMSE, JS and ACCU values for the cell type composition of spots across 30 MERFISH datasets, generated by 14 integration methods. Data were presented as mean values ± 95% confidence intervals; (F) The average Accuracy Score (which was aggregated from the PCC, SSIM, RMSE, JS and ACCU values) of the 14 methods. Data were presented as mean values ± 95% confidence intervals.

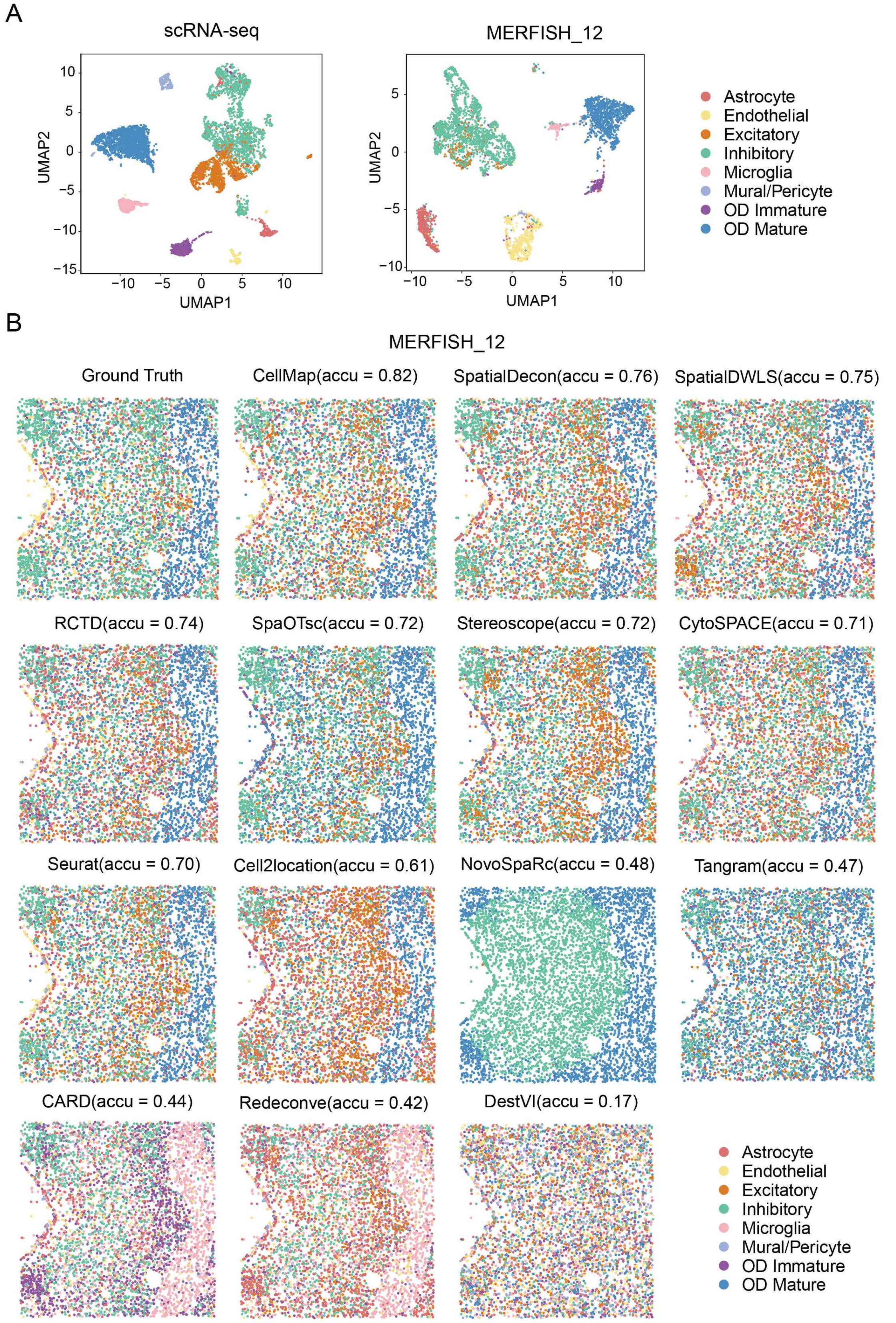

Figure S10. Benchmark CellMap on the MERFISH_12 ground truth dataset. (A) The UMAP layout depicting the clustering space of the scRNA-seq data and MERFISH data (Astrocyte, Endothelial, Excitatory, Inhibitory, Microglia, Mural/Pericyte, OD Immature and OD Mature) (ST

data ID: 12). The shared cell types were labeled with the respective corresponding colors. (B) The visualization results of eight cell types in the MERFISH_12 dataset across 14 integration methods, with the first panel in the first row representing the ground truth.

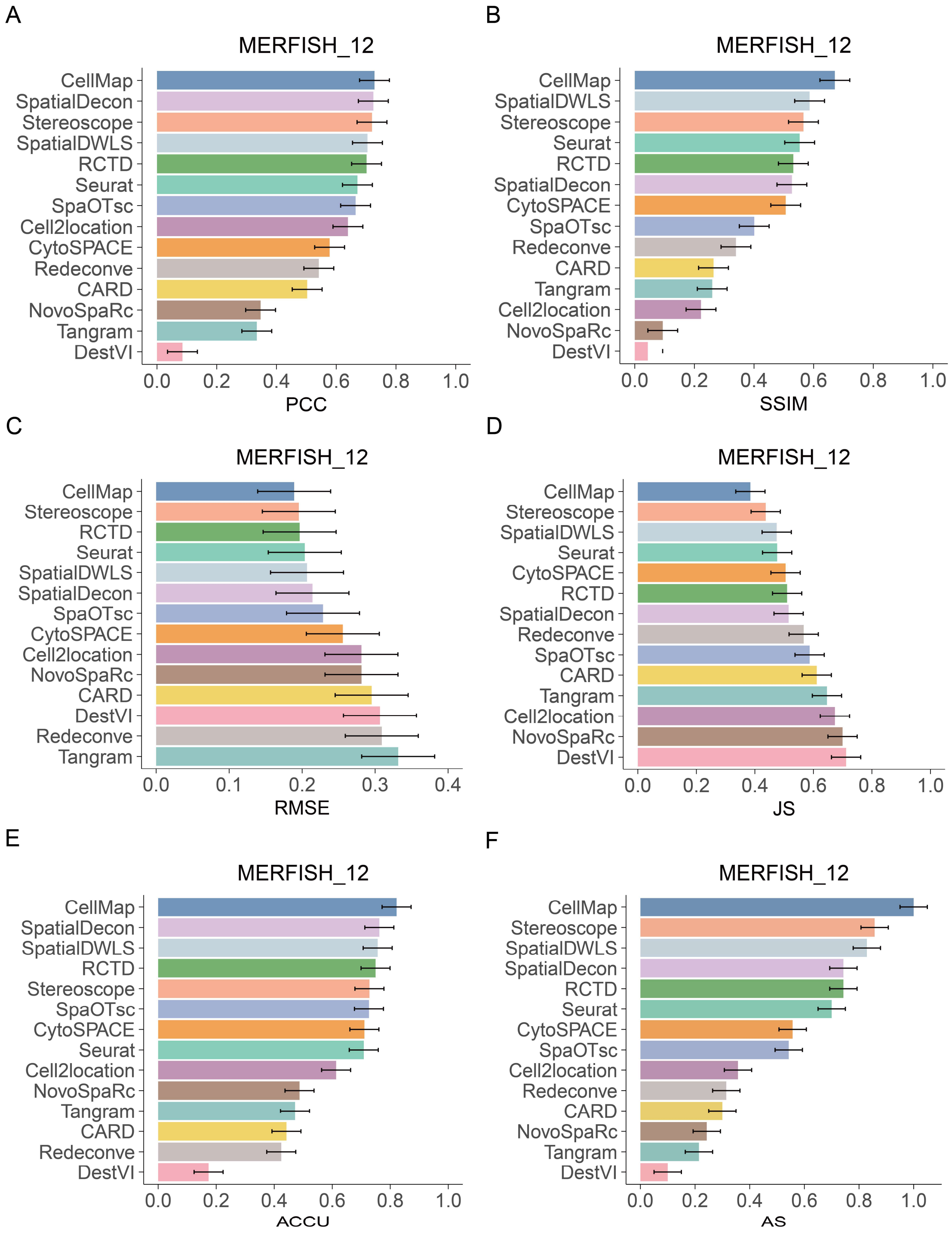

Figure S11. Comparison of the accuracy in predicting the spatial distribution of cell types across 14 methods in the MERFISH_12 dataset. (A-E) The bar plots displaying the PCC, SSIM, RMSE, JS and ACCU for each method in predicting distribution of cell types. Data are presented as mean values ± 95% confidence intervals; (F) The bar plot of AS (which is aggregated from the PCC, SSIM, RMSE, JS and ACCU values) of the 14 methods. Data are presented as mean values ± 95% confidence intervals.

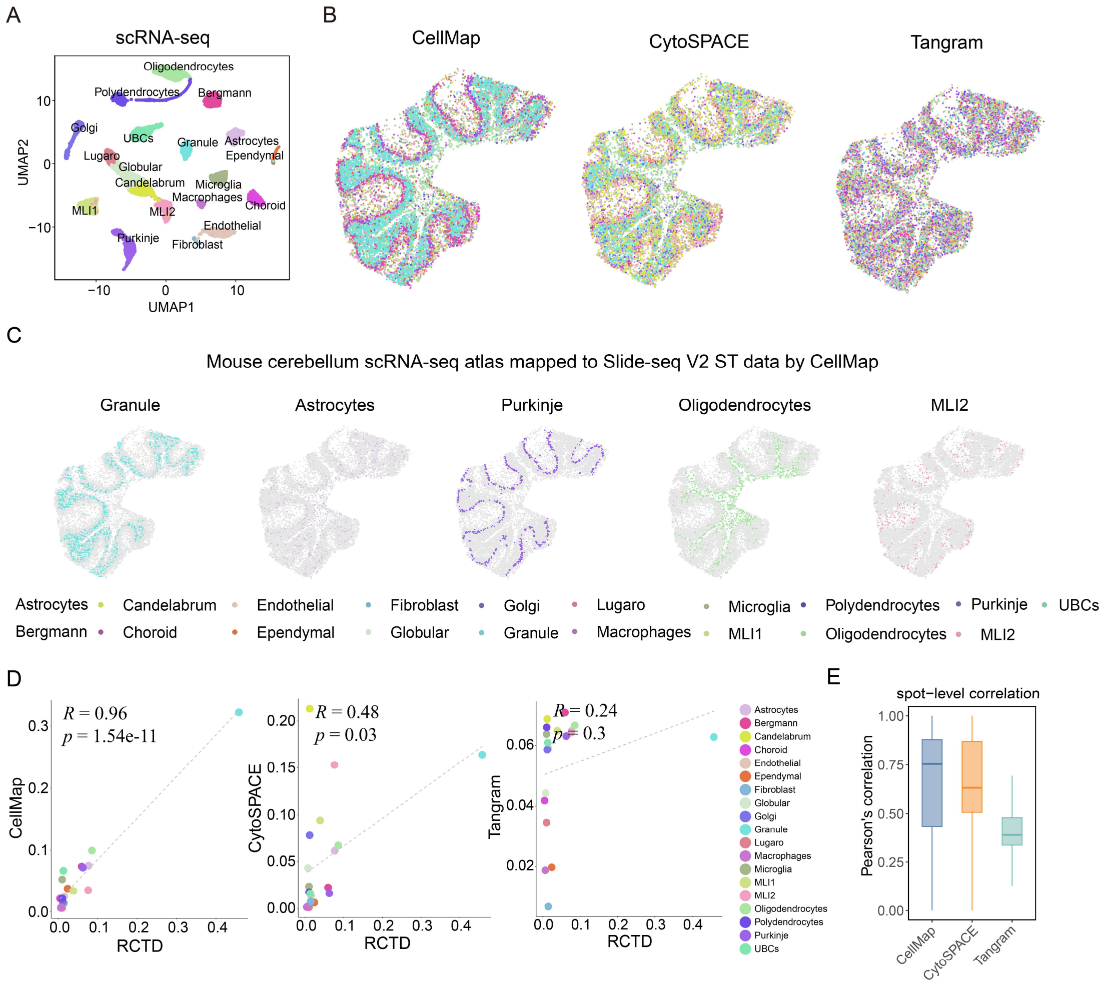

Figure S12. Performance assessment of CellMap on Slide-seq V2 data from mouse cerebellum. (A) The UMAP layout depicted the clustering space of mouse cerebellum scRNA-seq data. (B) Spatial structure of mouse cerebellum tissue reconstructed using CellMap, CytoSPACE and Tangram. The cell types are color-coded, with each dot representing an individual cell. (C) Spatial heat maps highlighting the cell type distribution in aligning scRNA-seq data to spatial locations in the Slide-seq V2 ST dataset (Granule, Astrocytes, Purkinje, Oligodendrocytes and MLI2). Cell types are color-coded, with each dot corresponding to a single cell. (D) Scatter plots depicting the consistency between cellular compositions in spatial single-cells maps reconstructed using CellMap, CytoSPACE and Tangram, and cellular compositions predicted by RCTD spatial deconvolution method. "*R*" represents the Pearson correlation coefficients and the p-values were obtained by t-test. (E) Benchmark of CellMap's performance with different mapping methods. The box plot reflects the overall distribution of Pearson's correlation calculated for each spot by various method.

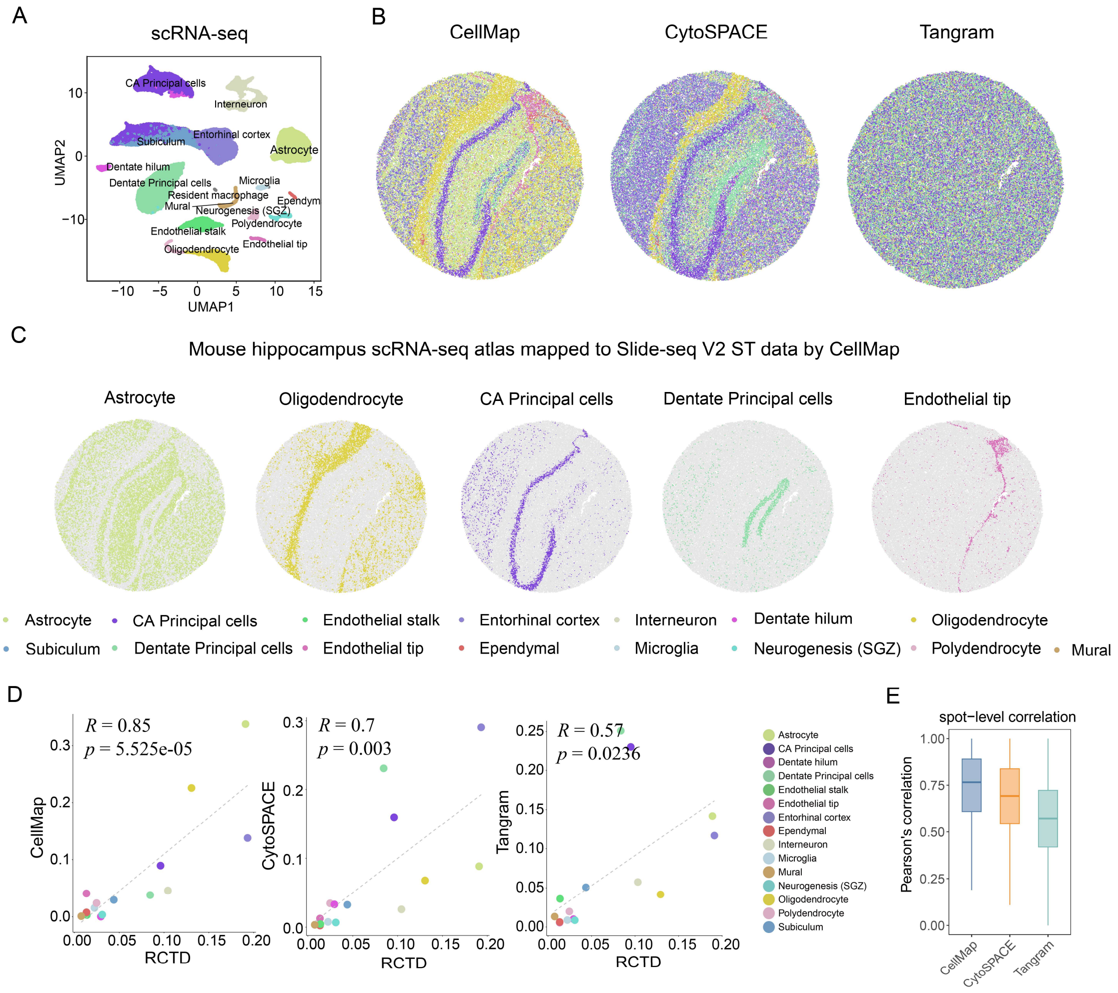

Figure S13. Performance assessment of CellMap on Slide-seq V2 data from mouse hippocampus. (A) The UMAP layout depicted the clustering space of mouse hippocampus scRNA-seq data. (B) Spatial structure of mouse hippocampus tissue reconstructed using CellMap, CytoSPACE and Tangram. The cell types are color-coded, with each dot representing an individual cell. (C) Spatial heat maps highlighting the cell type distribution in aligning scRNA-seq data to spatial locations in the Slide-seq V2 ST data (Astrocyte, Oligodendrocyte, CA Principal cells, Dentate Principal cells and Endothelial tip). Cell types are color-coded, with each dot corresponding to a single cell. (D) Scatter plots depicting the consistency between cellular compositions in spatial single-cells maps reconstructed using CellMap, CytoSPACE and Tangram, and cellular compositions predicted by RCTD spatial deconvolution method. "*R*" represents the Pearson correlation coefficients and the p-values were obtained by t-test. (E) Benchmark of CellMap's performance with different mapping methods. The box plot reflects the overall distribution of Pearson's correlation calculated for each spot by various method.

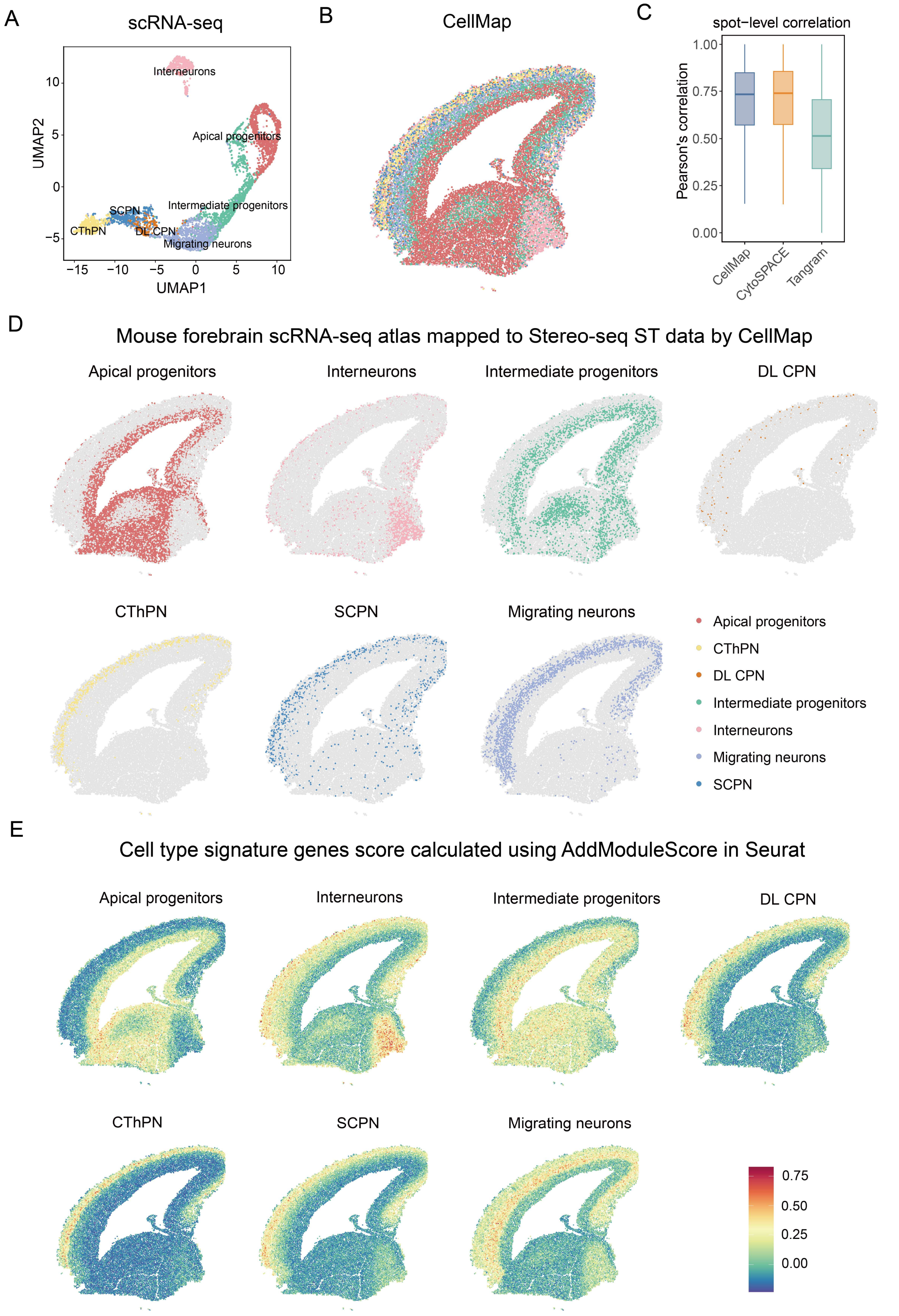

Figure S14. Performance assessment of CellMap on Stereo-seq data from mouse forebrain. (A) The UMAP layout depicting the clustering space of mouse developing cortex scRNA-seq data. The cell types are color-coded, with each dot representing an individual cell. (B) Spatial structure of mouse forebrain reconstructed using CellMap. (C) Benchmark of

CellMap's performance with different mapping methods. The box plot reflects the overall distribution of Pearson's correlation calculated for each spot by each method. (D) Spatial heat maps showing the spatial distribution of seven cell types predicted by CellMap in the Stereo-seq ST data, with each cell type highlighted in a different color. (E) Spatial heat maps showing cell type signature genes score calculated using *AddModuleScore* in Seurat. The colors from blue to red indicate the scores from low to high.

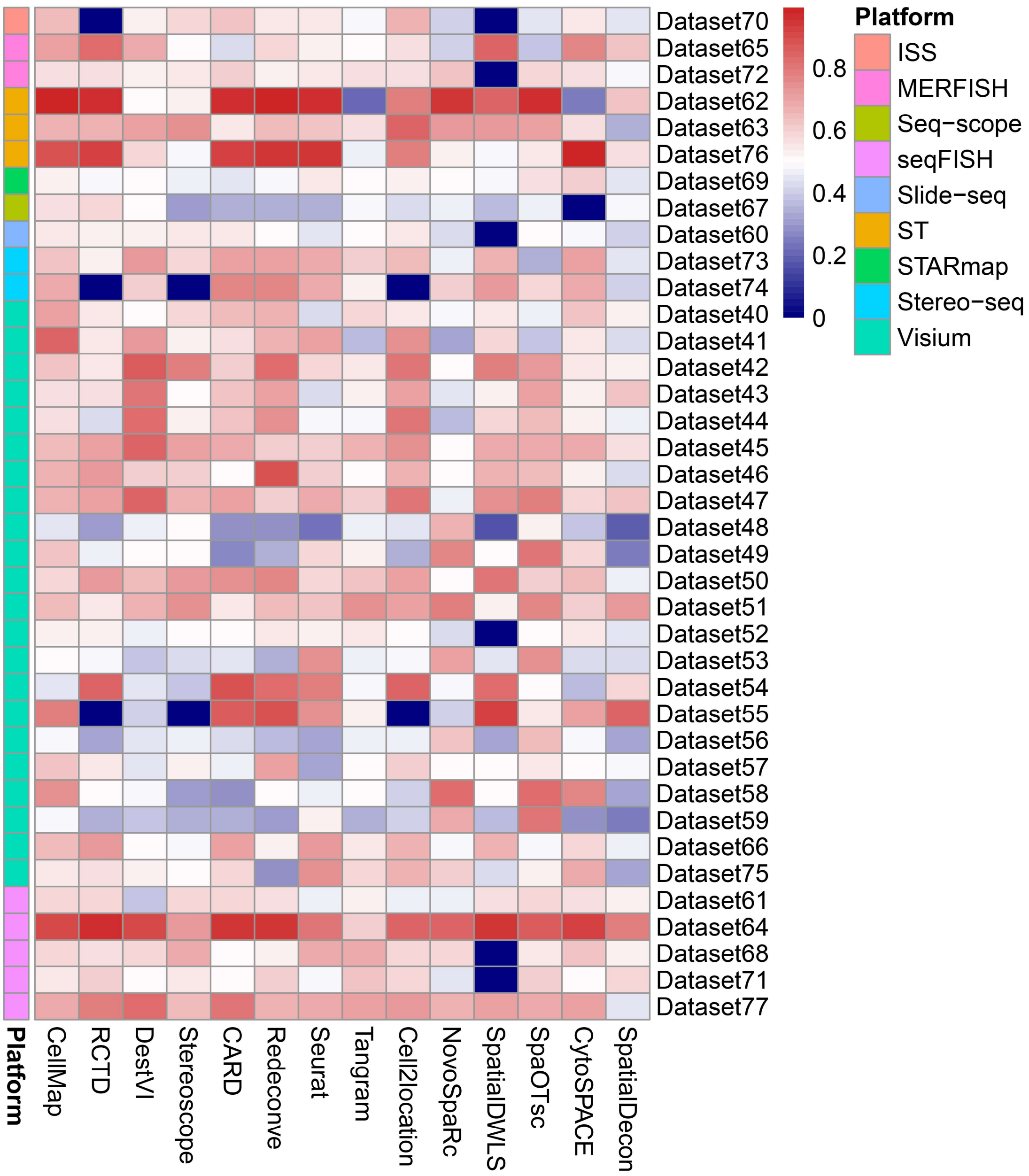

Figure S15. Heatmap of all paired of predicted average spot-level correlation between cell-type fractions and signature scores through 14 methods. In the heatmap, the color gradient from blue to red indicates correlations ranging from low to high. Several datasets contain NA values because: for Dataset55 and Dataset74, the raw count matrices of ST data were unavailable, preventing the execution of RCTD, Stereoscope, and Cell2location; for Dataset70, the ST data contained only 119 genes, making RCTD infeasible. SpatialDWLS failed to run on Dataset52, Dataset60, Dataset68, Dataset70, Dataset71, and Dataset72. CytoSPACE mapped only one cell type in Dataset67, resulting in NA.

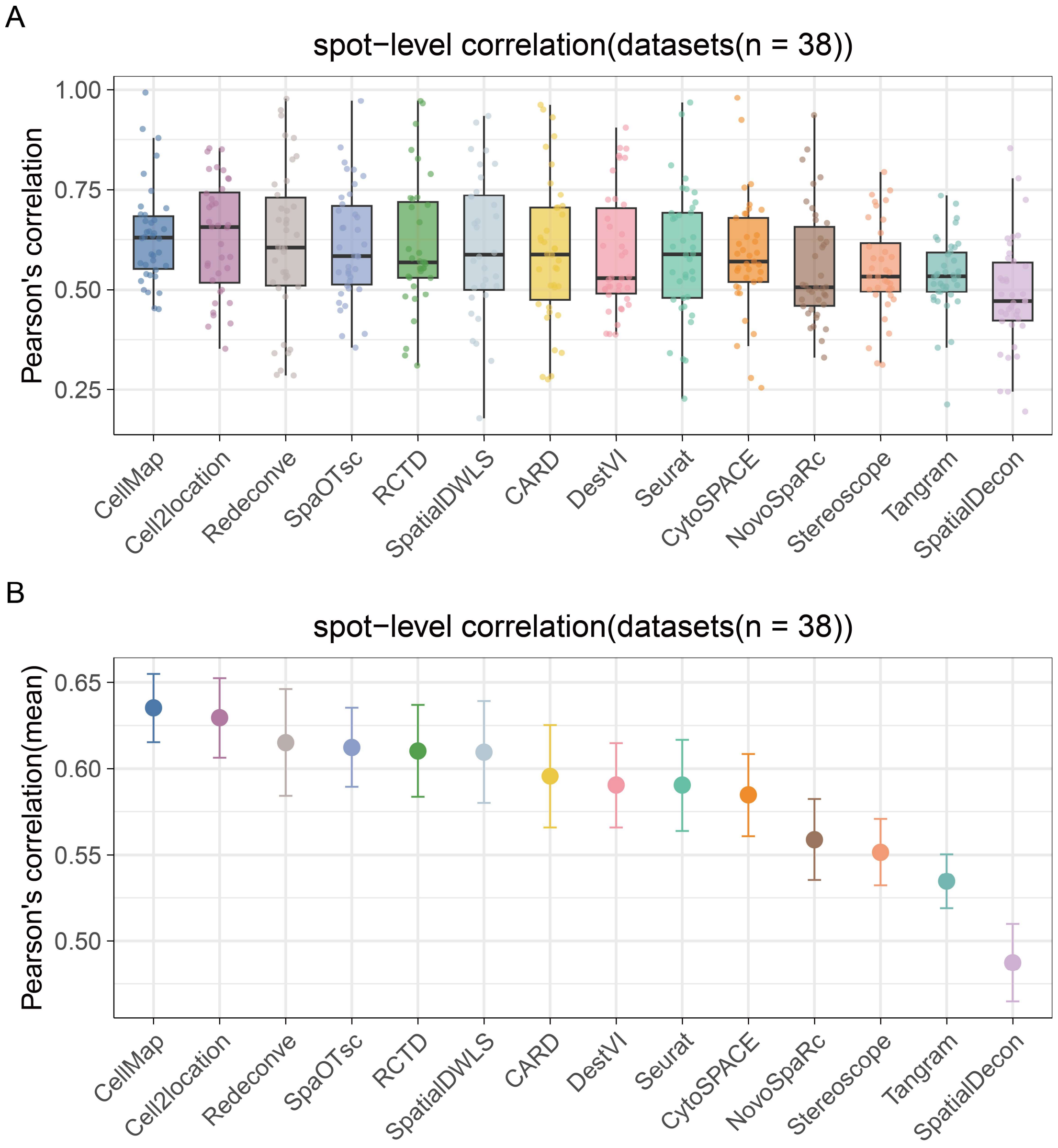

Figure S16. Benchmark of CellMap's performance with various methods across 38 datasets. (A) The boxplot reflects the overall distribution of mean Pearson's correlation for each dataset by 14 methods, with each point representing the mean Pearson's correlation of a single dataset. The methods are ordered according to their mean Pearson's correlation in descending order. (B) Mean Pearson's correlation was first calculated for each dataset, and then averaged across 38 datasets for each method. Points represent the overall mean correlation, and error bars indicate the standard error (± SE) across datasets.

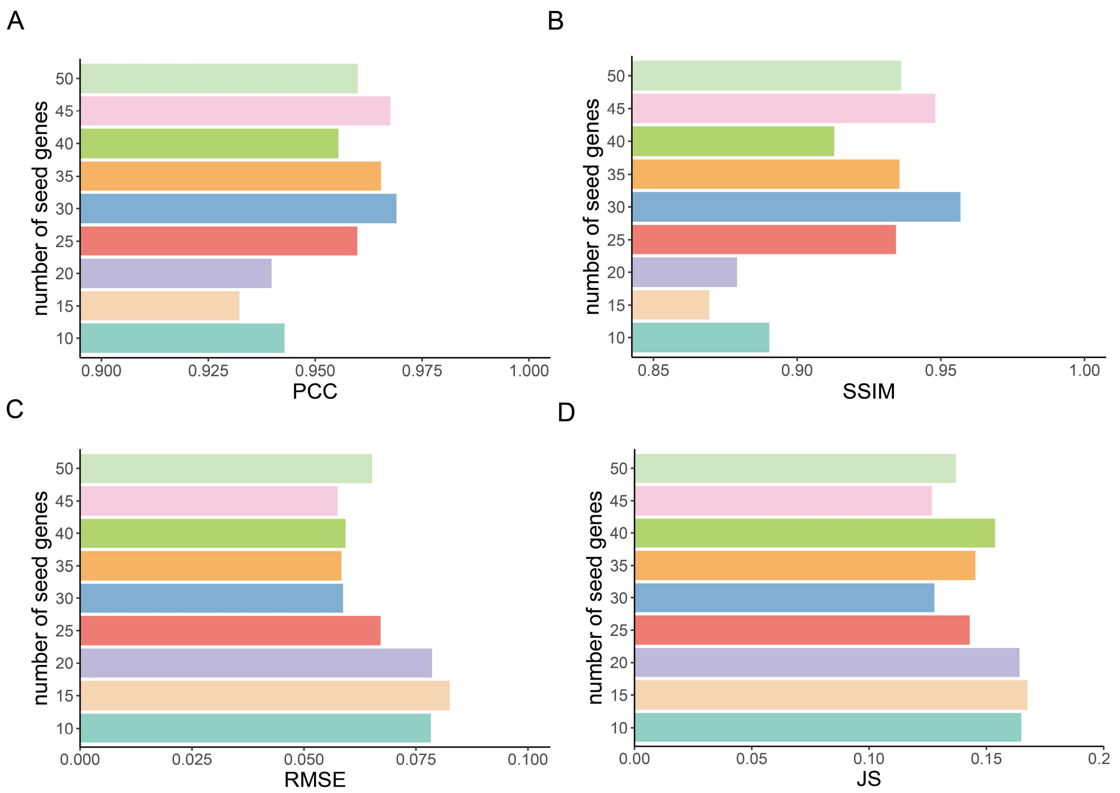

Figure S17. The bar plots of PCC (A), SSIM (B), RMSE (C) and JS (D) between the CellMap-predicted and true cell type proportions under different numbers of seed genes. For each metric, values were first computed per cell type and then averaged across all cell types.

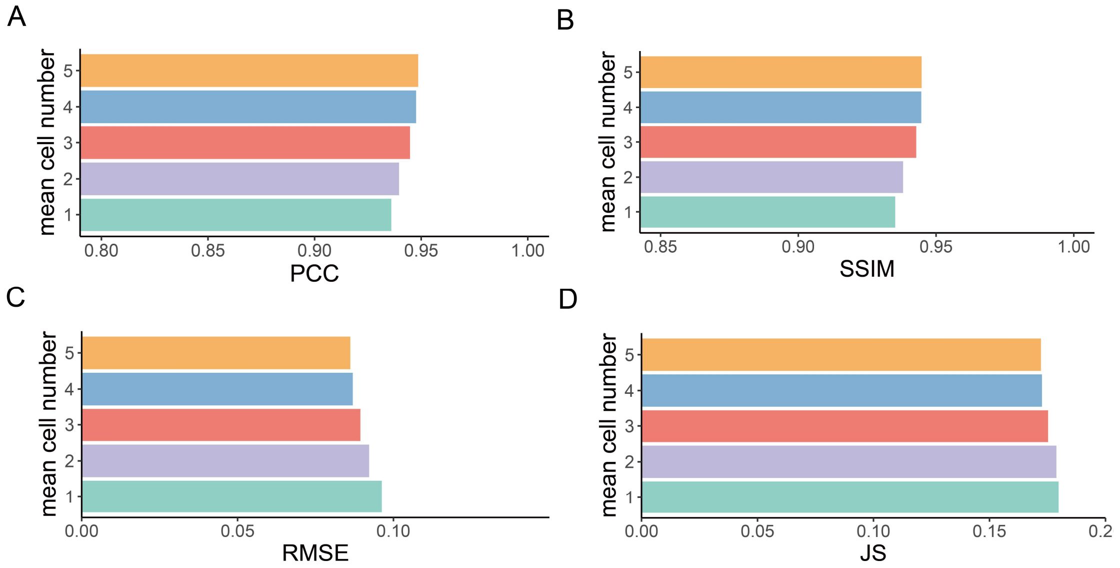

Figure S18. The bar plots of PCC (A), SSIM (B), RMSE (C) and JS (D) between the CellMap-predicted and true cell type proportions. For each metric, values were averaged across all cell types, and comparisons were made under different mean cell numbers per spot in high-resolution spatial transcriptomics data.

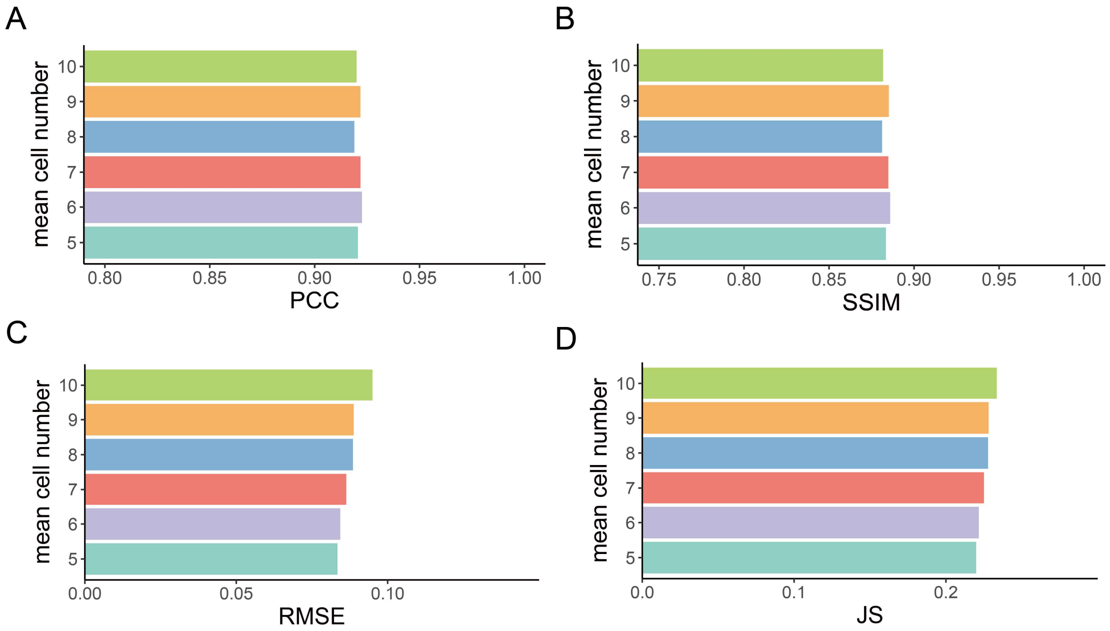

Figure S19. The bar plots of PCC (A), SSIM (B), RMSE (C) and JS (D) between the CellMap-predicted and true cell type proportions. For each metric, values were averaged across all cell types, and comparisons were made under different mean cell numbers per spot in low-resolution spatial transcriptomics data.

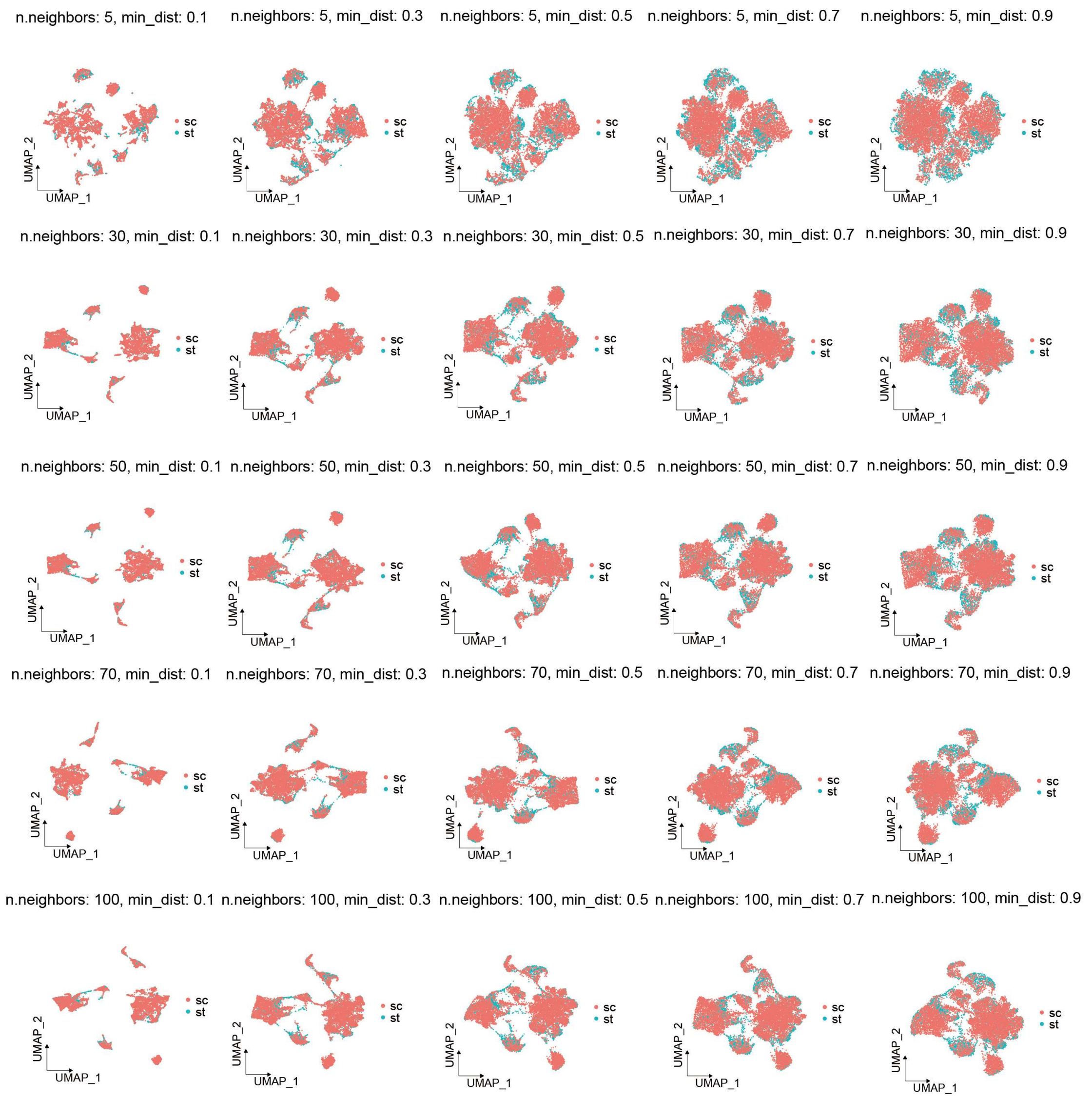

Figure S20. Single-cell and spatial transcriptomic data were embedded into a two-dimensional space under different parameter settings of MERFISH_1 dataset: n_neighbors as the size of the local neighborhood and min_dist as the effective minimum distance between embedded points. Single cells (sc) and spatial spots (st) are labeled with distinct colors, respectively.

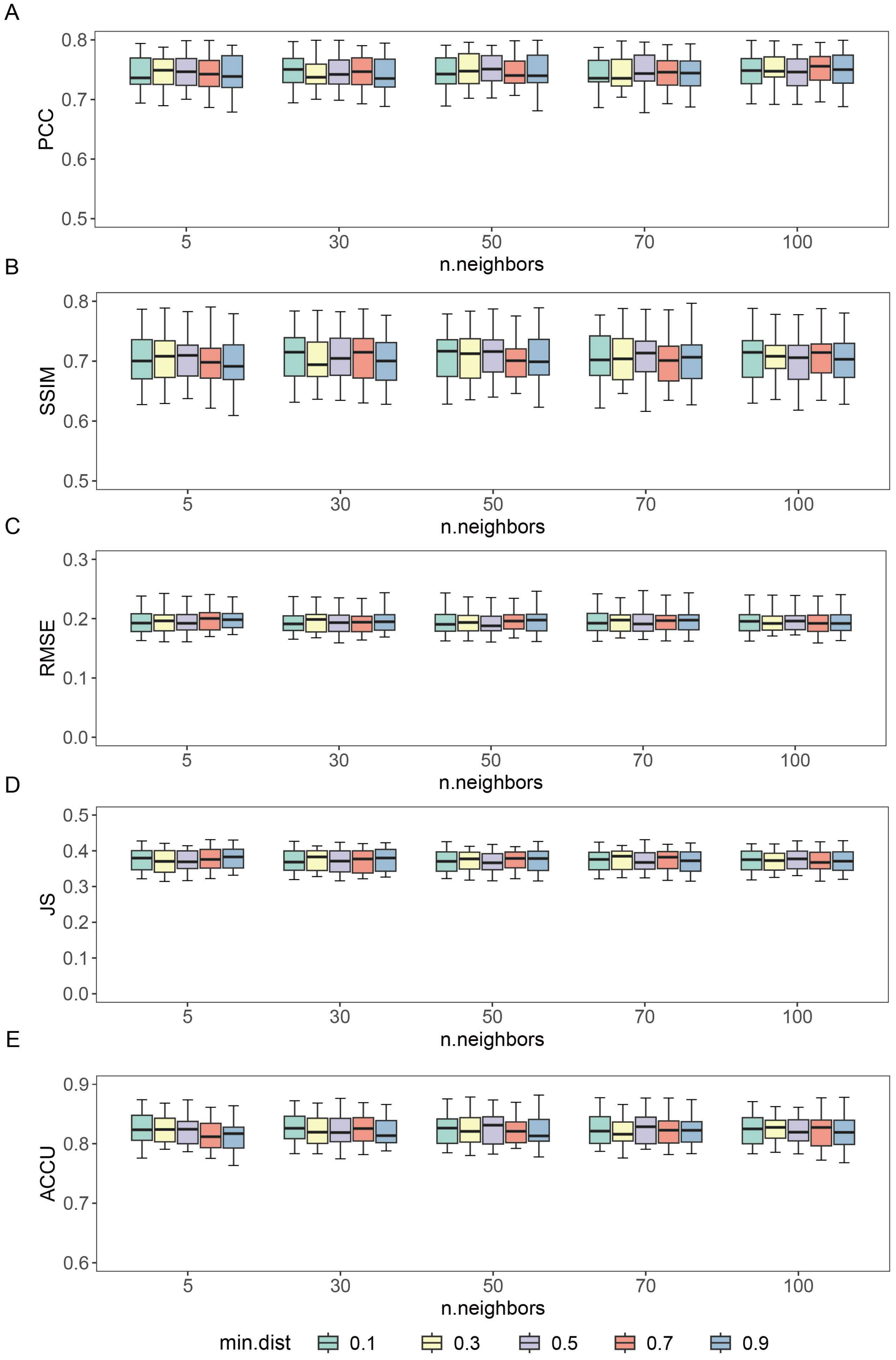

Figure S21. Performance of CellMap under different UMAP parameter settings across 30 MERFISH datasets. (A-E) Boxplots showing the PCC, SSIM, RMSE, JS and ACCU of CellMap under various UMAP parameter configurations (including n_neighbors and min_dist). Center line, median; box limits, upper and lower quartiles; whiskers, 1.5× interquartile range.

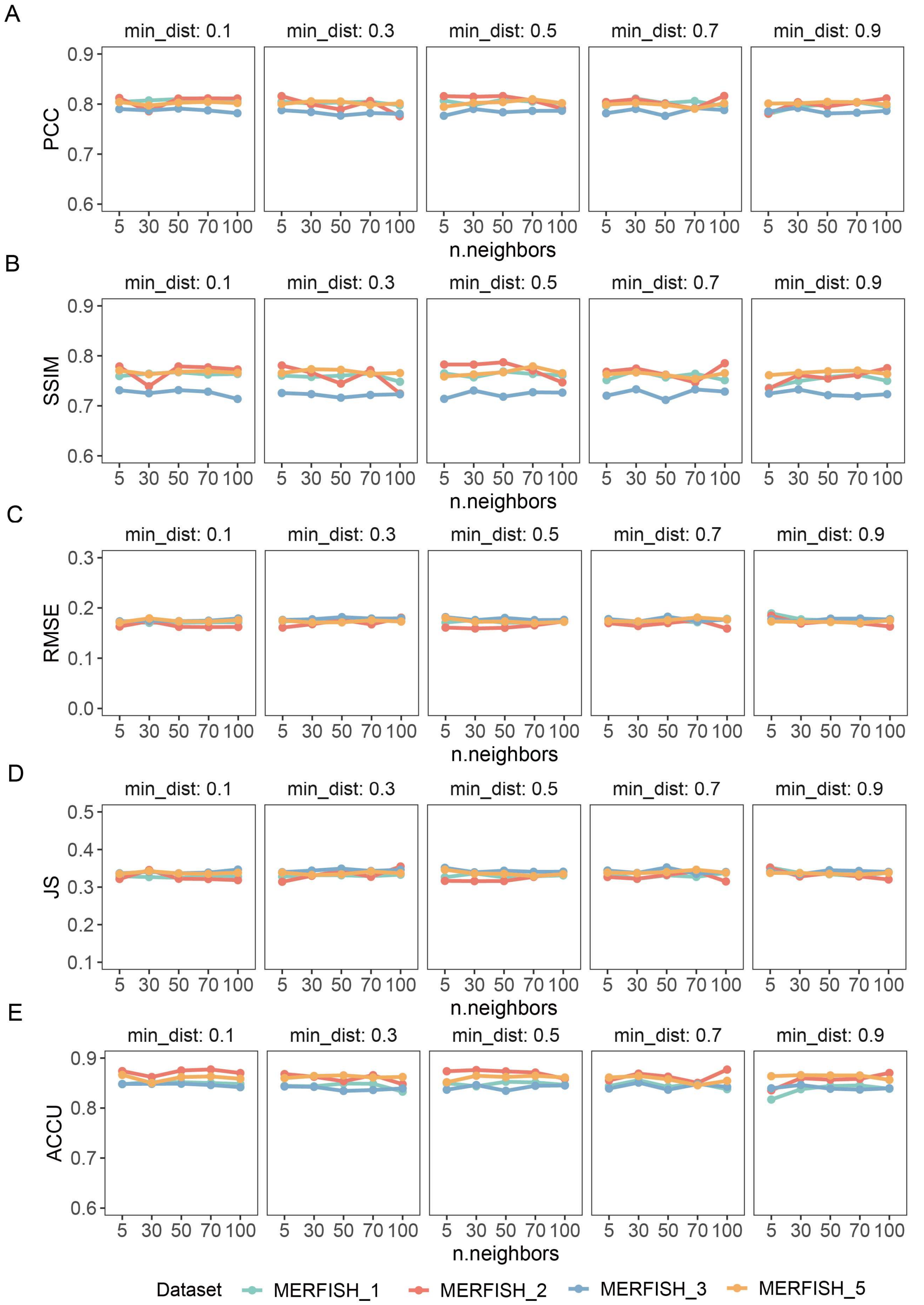

Figure S22. Performance of CellMap under different UMAP parameter across 4 MERFISH datasets. (A-E) Line plots show the PCC, SSIM, RMSE, JS and ACCU values of CellMap across 4 representative MERFISH datasets under varying UMAP parameter configurations (including n_neighbors and min_dist).

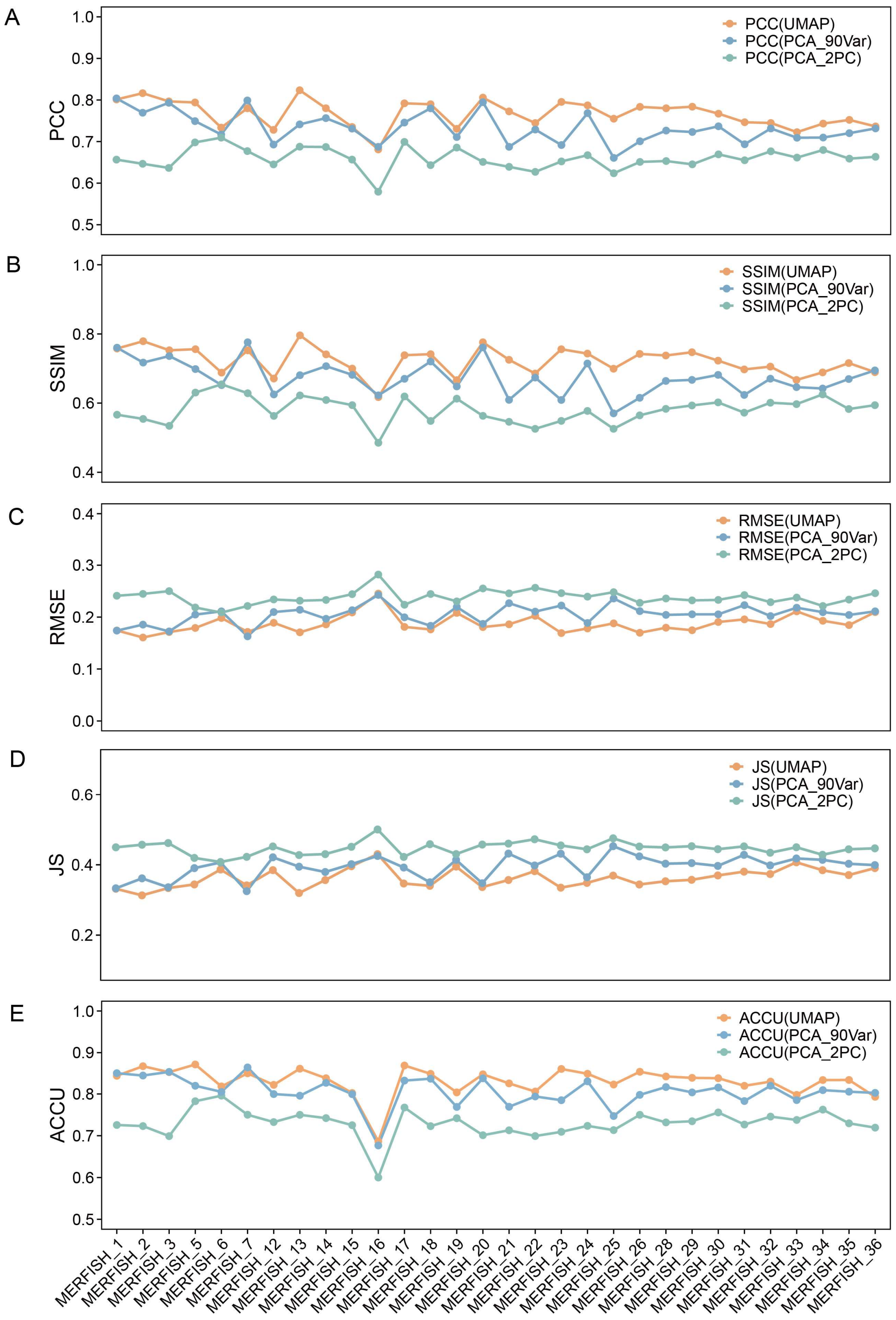

Figure S23. Benchmarking CellMap performance across dimensionality reduction methods. (A-E) Line plots displaying the PCC, SSIM, RMSE, JS, and ACCU of CellMap when evaluated with different dimensionality reduction methods. PCA_90Var denotes the use of principal component set explaining 90% of the total variance, while PCA_2PC indicates the use of only the first two principal components.

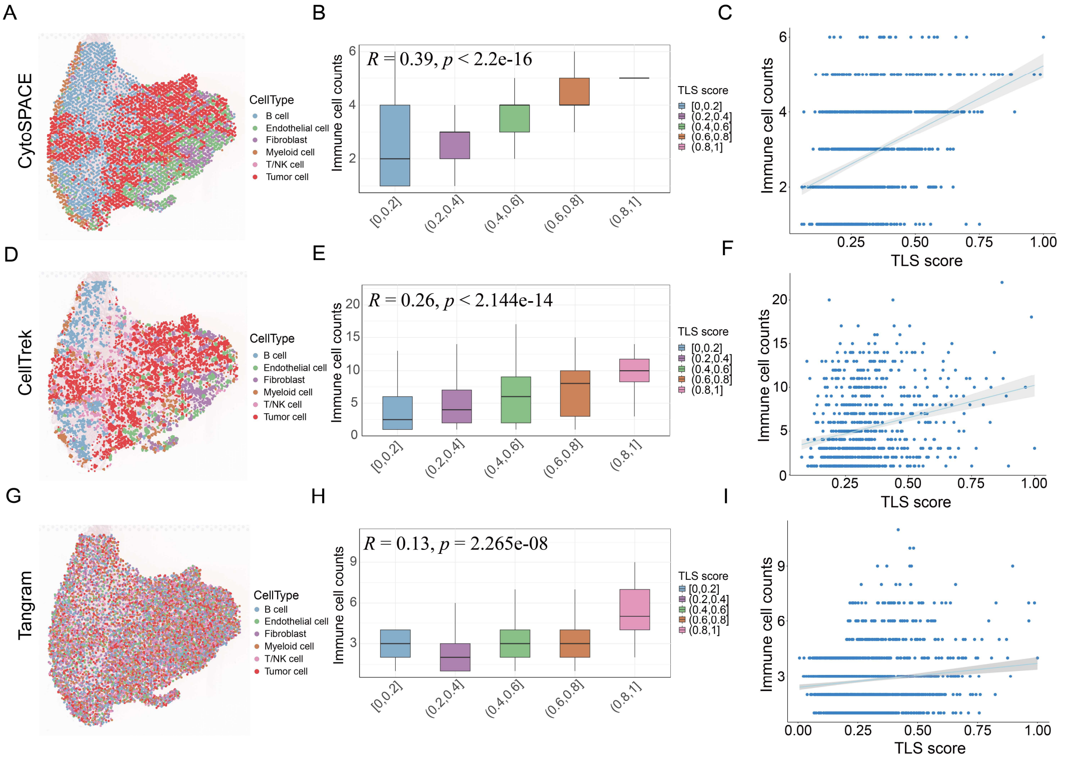

Figure S24. The comparative analysis of three methods in reconstructing critical structural regions within tissues. (A) The spatial single-cell map reconstructed by CytoSPACE. (B) The box plot illustrates the association between immune cell counts based on CytoSPACE map and TLS (tertiary lymphoid structures) score quantiles. A Pearson correlation test was performed. (C) The scatter plot depicting the association between immune cell counts and TLS scores, with the blue line represents linear fitting, and the shaded area indicates the 95% confidence interval. (D-F) The application analysis of CellTrek in reconstructing critical structural regions within tissues, as described above. (G-I) The application analysis of Tangram in reconstructing critical structural regions within tissues, as described above.

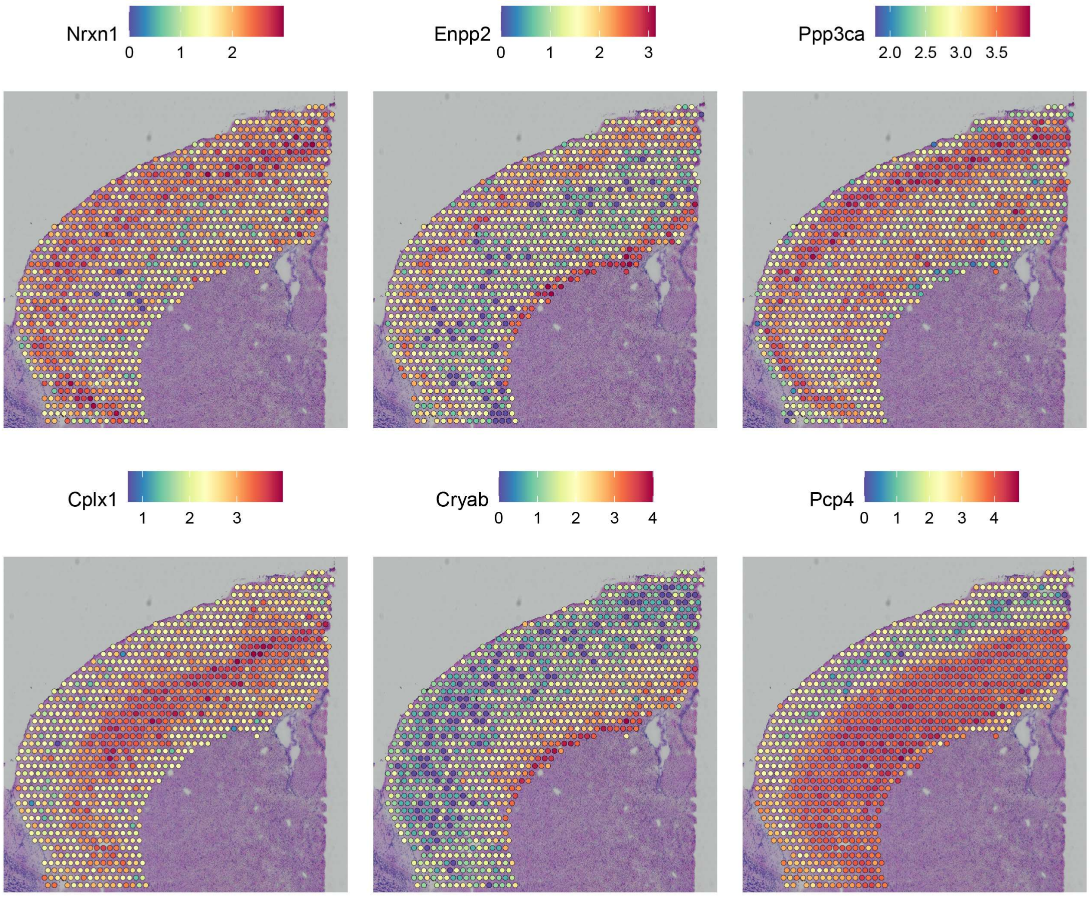

Figure S25. Expression of the top influential feature genes on the ST slice of the mouse cerebral cortex. Color scale from blue to red represents low to high normalized expression. To identify key feature genes critical for spatial mapping, we conducted a five-fold cross-validation. For each fold, we trained an independent Random Forest model and extracted the top 30 genes based on the MeanDecreaseGini metric. The robust set of key influential transcripts was defined as the consensus gene set derived from the intersection across all five folds. To validate the biological relevance of these identified genes, we visualized their spatial expression patterns on the ST data.

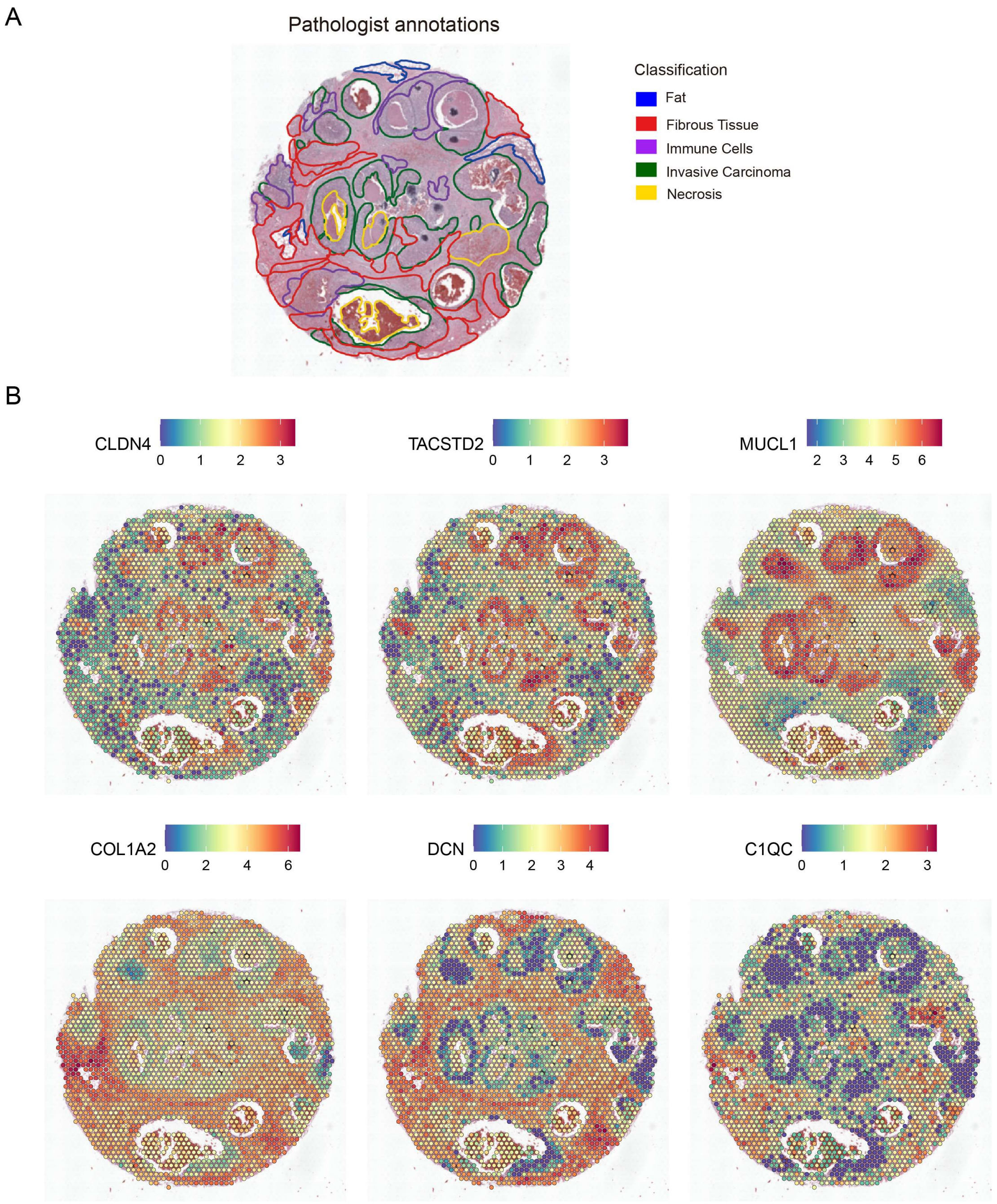

Figure S26. Expression of the top influential genes identifies key histological structures in human HER2+ breast cancer. (A) The pathological annotations of HER2+ breast cancer FFPE slice, including fat, fibrous tissue, immune cells, invasive carcinoma and necrosis. (B) Spatial expression patterns of the top influential feature genes identified by CellMap. Color scale from blue to red represents low to high normalized expression.

References

1. Vahid, M.R., Brown, E.L., Steen, C.B. *et al.* (2023) High-resolution alignment of single-cell and spatial transcriptomes with CytoSPACE. *Nature biotechnology*, **41**, 1543-1548.
2. Wei, R., He, S., Bai, S. *et al.* (2022) Spatial charting of single-cell transcriptomes in tissues. *Nature biotechnology*, **40**, 1190-1199.
3. Biancalani, T., Scalia, G., Buffoni, L. *et al.* (2021) Deep learning and alignment of spatially resolved single-cell transcriptomes with Tangram. *Nature methods*, **18**, 1352-1362.
4. Cable, D.M., Murray, E., Zou, L.S. *et al.* (2022) Robust decomposition of cell type mixtures in spatial transcriptomics. *Nature biotechnology*, **40**, 517-526.
5. Ma, Y. and Zhou, X. (2022) Spatially informed cell-type deconvolution for spatial transcriptomics. *Nature biotechnology*, **40**, 1349-1359.
6. Kleshchevnikov, V., Shmatko, A., Dann, E. *et al.* (2022) Cell2location maps fine-grained cell types in spatial transcriptomics. *Nature biotechnology*, **40**, 661-671.
7. Lopez, R., Li, B., Keren-Shaul, H. *et al.* (2022) DestVI identifies continuums of cell types in spatial transcriptomics data. *Nature biotechnology*, **40**, 1360-1369.
8. Moriel, N., Senel, E., Friedman, N. *et al.* (2021) NovoSpaRc: flexible spatial reconstruction of single-cell gene expression with optimal transport. *Nature protocols*, **16**, 4177-4200.
9. Zhou, Z., Zhong, Y., Zhang, Z. *et al.* (2023) Spatial transcriptomics deconvolution at single-cell resolution using Redeconve. *Nature communications*, **14**, 7930.
10. Dong, R. and Yuan, G.-C. (2021) SpatialDWLS: accurate deconvolution of spatial transcriptomic data. *Genome biology*, **22**, 145.
11. Elosua-Bayes, M., Nieto, P., Mereu, E. *et al.* (2021) SPOTlight: seeded NMF regression to deconvolute spatial transcriptomics spots with single-cell transcriptomes. *Nucleic acids research*, **49**, e50.
12. Andersson, A., Bergenstråhle, J., Asp, M. *et al.* (2020) Single-cell and spatial transcriptomics enables probabilistic inference of cell type topography. *Communications biology*, **3**, 565.
13. Stuart, T., Butler, A., Hoffman, P. *et al.* (2019) Comprehensive Integration of Single-Cell Data. *Cell*, **177**, 1888-1902.e1821.
14. Cang, Z. and Nie, Q. (2020) Inferring spatial and signaling relationships between cells from single cell transcriptomic data. *Nature communications*, **11**, 2084.
15. Danaher, P., Kim, Y., Nelson, B. *et al.* (2022) Advances in mixed cell deconvolution enable quantification of cell types in spatial transcriptomic data. *Nature communications*, **13**, 385.
